# Synthesis and Bioactivity of Ancorinoside B, a Marine Diglycosyl Tetramic Acid

**DOI:** 10.3390/md19100583

**Published:** 2021-10-19

**Authors:** Kevin J. Soliga, Sofia I. Bär, Natalie Oberhuber, Haoxuan Zeng, Hedda Schrey, Rainer Schobert

**Affiliations:** 1 Department of Chemistry, University Bayreuth, Universitaetsstr. 30, D-95440 Bayreuth, Germany; kevin.soliga@t-online.de (K.J.S.); Sofia.Baer@uni-bayreuth.de (S.I.B.); Natalie.Oberhuber@uni-bayreuth.de (N.O.); 2 Department of Microbial Drugs, Helmholtz Centre for Infection Research GmbH, Inhoffenstrasse 7, 38124 Braunschweig, Germany; haoxuan.zeng@helmholtz-hzi.de (H.Z.); hedda.schrey@helmholtz-hzi.de (H.S.)

**Keywords:** glycosyl tetramic acid, ancorinoside B, marine sponge metabolite, microbial biofilm inhibitor, MMP inhibitor

## Abstract

The sponge metabolite ancorinoside B was prepared for the first time in 16 steps and 4% yield. It features a β-d-galactopyranosyl-(1→4)-β-d-glucuronic acid tethered to a d-aspartic acid-derived tetramic acid. Key steps were the synthesis of a fully protected d-lactose derived thioglycoside, its attachment to a C_20_-aldehyde spacer, functionalization of the latter with a terminal *N*-(β-ketoacyl)-d-aspartate, and a basic Dieckmann cyclization to close the pyrrolidin-2,4-dione ring with concomitant global deprotection. Ancorinoside B exhibited multiple biological effects of medicinal interest. It inhibited the secretion of the cancer metastasis-relevant matrix metalloproteinases MMP-2 and MMP-9, and also the growth of *Staphylococcus aureus* biofilms by ca 87% when applied at concentrations as low as 0.5 µg/mL. This concentration is far below its MIC of ca 67 µg/mL and thus unlikely to induce bacterial resistance. It also led to a 67% dispersion of preformed *S. aureus* biofilms when applied at a concentration of ca 2 µg/mL. Ancorinoside B might thus be an interesting candidate for the control of the general hospital, catheter, or joint protheses infections.

## 1. Introduction

**Figure 1:**
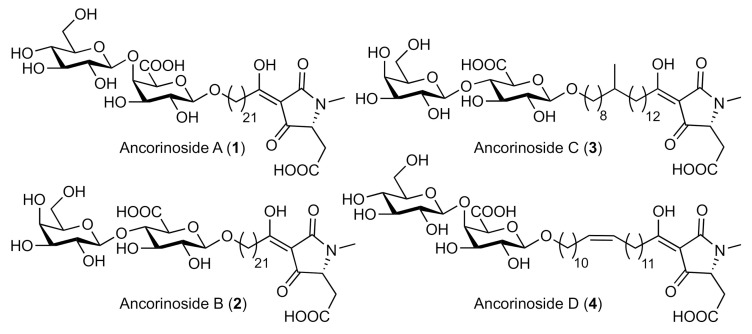
Structures of ancorinosides A–D (**1**–**4**).

Tetramic acids with glycosylated 3-acyl sidechains occur in nature as metabolites of bacteria, fungi/molds, and sponges. Their bioactivities span a broad spectrum, including antifungal, antibacterial, cytotoxic, and specific protein inhibitory effects [1,2,3,4]. They differ considerably in terms of structural complexity, culminating in compounds such as the aflastatins [5], which are fraught with functional groups and stereogenic centers. However, structural intricacy is not a prerequisite for biological activity. The ancorinosides A–D (**1**–**4**; Figure 1) were isolated from marine sponges *Ancorina* sp. and *Penares sollasi* by the groups of Ohta et al. [6] and Fusetani et al. [7]. They were found to inhibit membrane-type matrix metalloproteinases (MT1-MMPs) which are relevant for tumor growth and metastasis, and feature unadorned alkyl tethers and either a terminal β-d-glucopyranosyl-(1→4)-β-d-galacturonic acid (for **1** and **4**) or a β-d-galactopyranosyl-(1→4)-β-d-glucuronic acid residue (for **2** and **3**). Previously, we reported syntheses of ancorinosides A (**1**) [8] and D (**4**) [9], starting from d-glucose and d-galactose building blocks, and a d-aspartic acid ester. As this concept was not applicable to the disaccharide motif of ancorinosides B (**2**) and C (**3**), we now developed an alternative approach starting with inexpensive d-lactose.

## 2. Results and Discussion

### 2.1. Synthesis of Ancorinoside B (**2**)

**Scheme 1:**
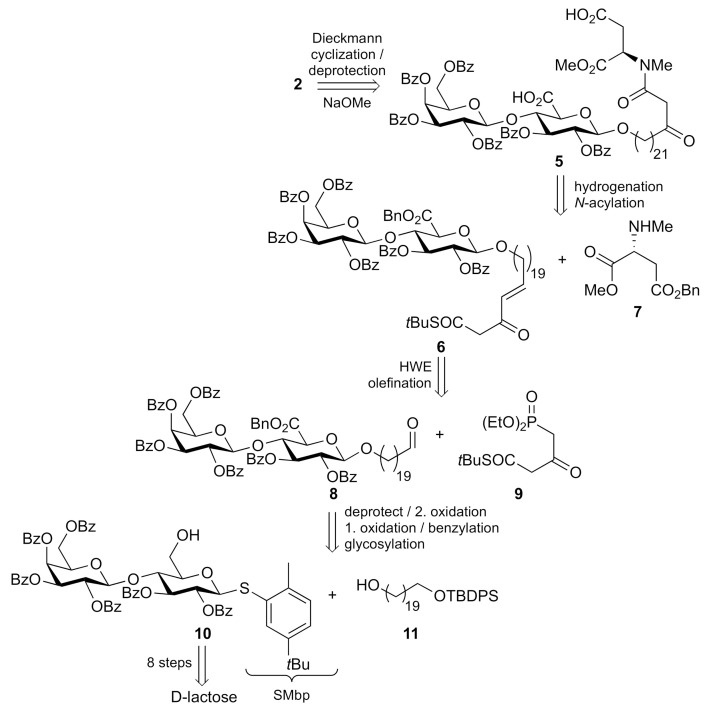
Retrosynthesis of ancorinoside B (**2**).

Our retrosynthetic approach is outlined in Scheme 1. The immediate precursor **5**, a β-ketoamide bearing two carboxylic acids, was to undergo a base-induced Dieckmann cyclization to give the 2,4-pyrrolidinone, with concomitant cleavage of the methyl ester and benzoate protecting groups.

Amide **5** should be prepared by an *N*-acylation [10] of *N*-methylated methyl d-aspartate **7** with unsaturated β-ketothioester **6**, both having their carboxylic acids protected as benzyl esters, which were subsequently hydrogenated together with the alkene. Thioester **6** was thought accessible via Horner-Wadsworth-Emmons (HWE) olefination between an aldehyde **8** and readily available [10] phosphonate **9**. While this sequence, so far, is reminiscent of the final steps of our route to ancorinoside A (**1**) [8], the synthesis of the required aldehyde **8**, and its precursor disaccharides had to be planned distinctly different. Aldehyde **8** was to originate from β-selective glycosylation between a lactose-derived thioglycoside donor **10**, fully benzoate protected except for the primary hydroxy group on the glucose moiety, and from a monoprotected 1,20-eicosanediol **11**, followed by oxidation affording a uronic acid, its esterification, and eventually a deprotection and oxidation of the terminal hydroxy group.

**Scheme 2:**
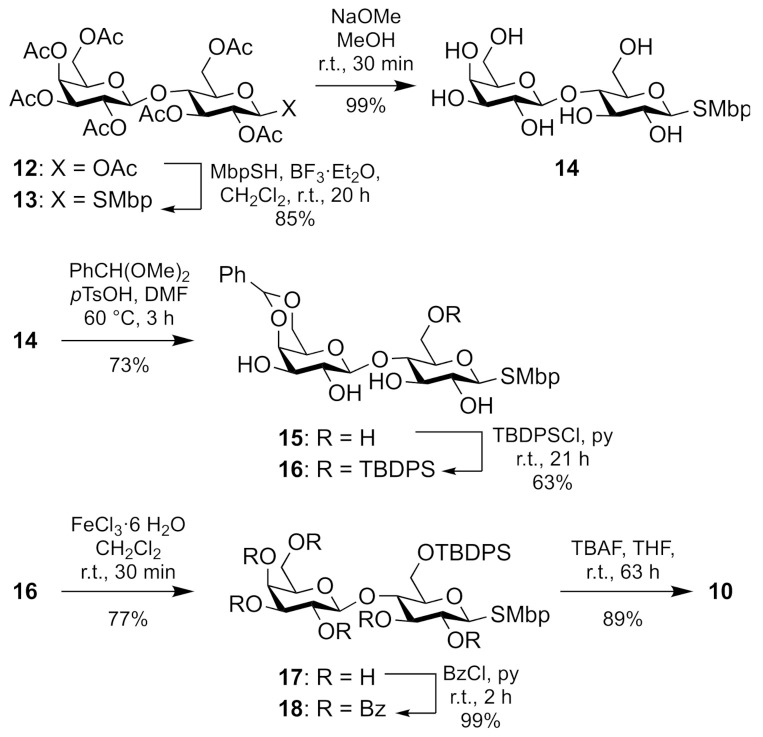
Seven-step synthesis of diglycoside donor **10**.

The diglycoside donor **10** was prepared from commercially available, or readily synthesizable peracetylated d-lactose **12** in seven steps and 26% yield (Scheme 2). Compound **12** was substituted with 2-methyl-5-*tert*-butyl-thiophenol (MbpSH) at C-1 to leave thioglycoside **13** in 85% yield. Saponification of all acetates gave unprotected disaccharide **14** which was selectively protected in the galactose unit as a 4,6-benzylidene acetal **15** in 73% yield. Protection of the remaining primary alcohol as a *tert*-butyldiphenylsilyl (TBDPS) ether afforded compound **16**. Removal of the 4,6-benzylidene acetal with ferric chloride furnished compound **17** which was per-benzoylated to give fully protected compound **18**. Selective cleavage of the silyl ether eventually gave the desired diglycoside donor **10**.

**Scheme 3:**
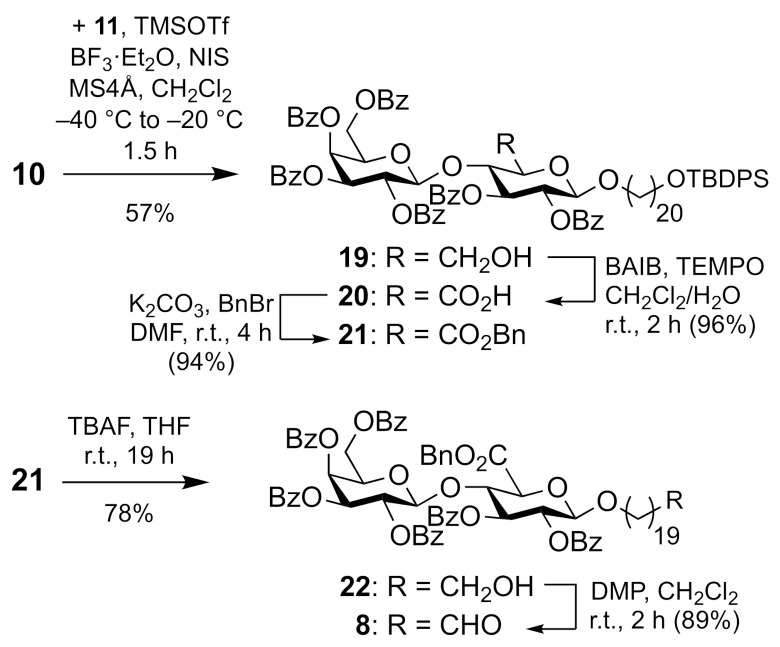
Synthesis of aldehyde **8**.

Donor **10** was then used to β-selectively glycosylate the monoprotected spacer diol **11** under standard conditions [11], affording tethered disaccharide **19** (Scheme 3). Alcohol **11** was prepared from eicosanedioic acid in two steps and 34% yield, analogously to the known TBS-protected congener [8]. Alcohol **19** was oxidized with bis(acetoxy)iodobenzene (BAIB)/TEMPO to uronic acid **20** in almost quantitative yield. Subsequent esterification gave benzyl ester **21** in 94% yield. Cleavage of the silyl ether with TBAF liberated 1-alkanol **22** which was oxidized to key aldehyde **8** with Dess-Martin periodinane (DMP).

**Scheme 4:**
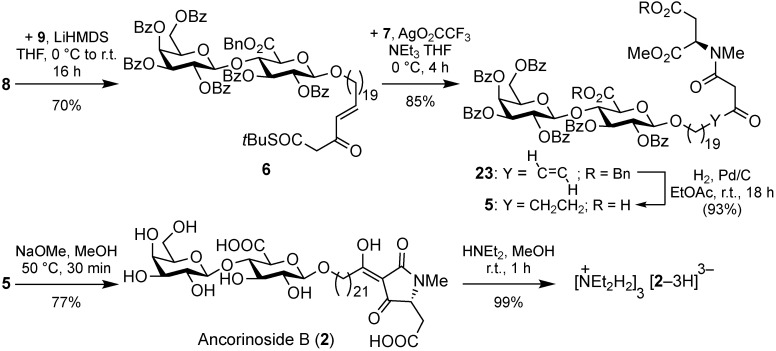
Synthesis of ancorinoside B (**2**) and its tris(diethylammonium) salt.

Aldehyde **8** was chain-lengthened by a HWE olefination with Ley’s phosphonate **9 [10]** to give β-ketothioester **6** which was submitted to a silver-mediated coupling reaction with d-aspartate **7** furnishing β-ketoamide **23** (Scheme 4). The catalytic hydrogenation of both the alkene and the benzyl esters of the latter afforded compound **5**, the immediate precursor to ancorinoside B (**2**). The latter was obtained upon a base-induced Dieckmann cyclization of the *N*-(β-ketoacyl) amino ester moiety of **5** with concomitant global deprotection of its diglycoside unit. The total yield over 16 steps was 4% (longest linear sequence). As Fusetani et al. [7] isolated and analyzed ancorinoside B as its tris(diethylammonium) salt, we also prepared this by treating target compound **2** with an excess of diethylamine in methanol. The ^1^H and ^13^C NMR data of our synthetic ammonium salt (NEt_2_H_2_)_3_[**2**–3H] are in good accordance with those published for the ammonium salt of the isolate (*cf*. Table S1 in the Supplementary Materials). While Fusetani et al. reported a specific optical rotation of [α]^24^_D_ +1.5 (*c* 0.1, MeOH) for the ammonium salt, our synthetic products showed specific optical rotations of [α]^24^_D_ +5.0 (*c* 0.1, MeOH) for the ammonium salt, and [α]^24^_D_ +3.2 (*c* 0.1, MeOH) for the neutral ancorinoside B (**2**).

### 2.2. Biological Activities of Ancorinoside B (**2**)

Synthetic ancorinoside B (**2**) was evaluated for antimicrobial (including antibiofilm) and cytotoxic activity against various bacteria, fungi, and cell lines. Its Minimum Inhibitory Concentration (MIC) values showed that it was only moderately to weakly active against four out of twelve species (Table 1). Against *Bacillus subtilis* with a MIC value of 16.6 μg/mL which is of the same order as the MIC = 4.2 µg/mL of oxytetracycline used as a positive control, against *Staphylococcus aureus* with MIC = 66.6 µg/mL (oxytetracycline: 0.4 µg/mL), against *Mucor hiemalis* with MIC = 66.6 μg/mL (positive control nystatin: 8.3 µg/mL), and against *Rhodotorula glutinis* with MIC = 66.6 µg/mL (nystatin: 2.1 µg/mL).

Ancorinoside B (**2**) also interfered with the formation and persistence of bacterial biofilms at concentrations far below its MIC and thus probably not inducing bacterial resistance via selection (Table 2). It inhibited the formation of biofilms of *Staphylococcus aureus* by 87% relative to untreated cultures at concentrations as low as 0.5 µg/mL, which is far superior to the known biofilm inhibitor microporenic acid A (MAA) [12], which led to a similar inhibition only at a high concentration of ca 31 µg/mL. Ancorinoside B (**2**) also led to the dispersion of preformed *S. aureus* biofilms to an extent of 67% when applied at a concentration of ca 2 µg/mL. In comparison, the positive control MAA, when applied at the maximum concentration of 250 µg/mL caused a dispersion of only ca 62%. Ancorinoside B (**2**) also dispersed 55% of preformed biofilms of *Candida albicans*, when applied at the highest concentration of 250 µg/mL. It is worthy of note that ancorinoside B (**2**) did not interfere in a similar manner with biofilms of *Pseudomonas aeruginosa*.

Synthetic ancorinoside B (**2**) was also tested for cytotoxicity against mouse fibroblast cells L929 and human endocervical adenocarcinoma KB3.1 cells, yet was found inactive. This bodes well for controllable toxicity in animals and humans.

**Figure 2:**
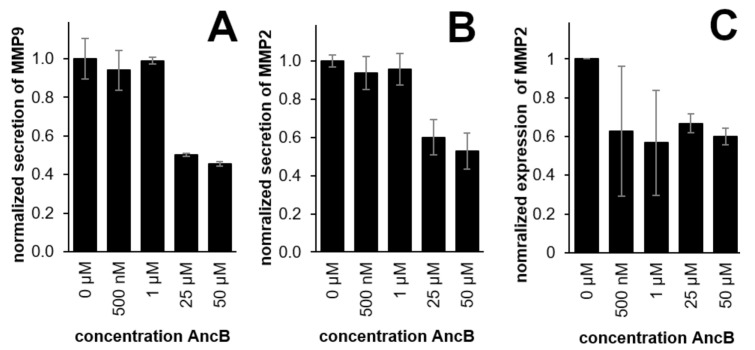
Effect of 500 nM to 50 µM of synthetic ancorinoside B (**2**; AncB) on the secretion of MMP-9 (**A**), the secretion of MMP-2 (**B**), and the expression of MMP-2 (**C**) in 518A2 melanoma cells; large error bars in **C** are due to blurry electrophoresis bands. DMSO (0 µM) served as a control.

Fusetani et al. [7] reported their isolated ancorinoside B (**2**) to inhibit the activity of purified membrane-type 1 matrix metalloproteinase and the downstream, tumor-relevant gelatinase A (MMP-2). We now examined whether synthetic ancorinoside B showed a similar effect on a cellular level by monitoring the expression and secretion of human cancer metastasis relevant MMP-2 and MMP-9 in treated 518A2 melanoma cells via gelatin zymography. In line with Fusetani et al. who reported an IC_50_ of 33 µg/mL (ca. 39 µM) for the inhibition of the activity of MMP-2 by their isolated ancorinoside B, we found that synthetic ancorinoside B reduced the secretion of human MMP-2 and MMP-9 by 518A2 melanoma cells in a concentration-dependent manner to ca 50% when applied at concentrations of 25–50 µM. It also reduced the expression of MMP-2 to ca 60% when applied at 50 µM (Figure 2 and Figure S1).

## 3. Materials and Methods

### 3.1. General Information

Infrared spectra were recorded on a PerkinElmer Spectrum 100 FT-IR spectrophotometer(PerkinElmer, Rodgau, Germany) with an ATR sampling unit. Optical rotations were measured at 589 nm (Na-D line) on a PerkinElmer 241 polarimeter; [α]_D_ values are quoted in 10^−1^ deg cm^2^ g^−1^. High-resolution mass spectra were recorded with a UPLC/Orbitrap MS system in ESI mode. NMR spectra were recorded with a Bruker Avance III HD 500 spectrometer (Bruker, Billerica, MA, USA; ^1^H NMR: 500 MHz and ^13^C NMR: 125 MHz). Chemical shifts are listed in parts per million relative to residual solvent peak as an internal standard, 7.26 ppm (proton) and 77.00 ppm (carbon) for CDCl_3_, 3.31 ppm (proton) and 49.15 ppm (carbon) for MeOD and 2.50 ppm (proton) and 39.51 ppm (carbon) for DMSO-d_6_. Coupling constants (*J*) are quoted in Hz. Multiplicities are quoted as: bs broad singlet, s singlet, d doublet, t triplet, q quartet, quin quintet, and m multiplet. Melting points were recorded on a Büchi Melting Point M-565 (Büchi Labortechnik, Flawil, Switzrland) and are uncorrected. All reagents were bought from Sigma-Aldrich (St. Louis, MO, USA) and TCI Chemicals (Zwijndrecht, Belgium) and were used without further purification. Anhydrous solvents were used as supplied, except THF and CH_2_Cl_2_ which were freshly distilled according to standard protocols. Reactions were routinely carried out under an argon atmosphere unless stated otherwise. All glassware was flame-dried prior to use. Analytical thin-layer chromatography (TLC) was carried out using Merck Kieselgel 60 F254 pre-coated aluminum-backed plates. Compounds were visualized with UV light (254 nm) and/or stained with ceric ammonium molybdate (CAM). Flash chromatography was performed at medium pressure using Macherey-Nagel (Macherey-Nagel, Düren, Germany) silica gel 60, pore size 40–63 μm with the eluent specified. Eicosan-1,20-diol, d-*N*-Me-Asp(OBn)-OMe (**7**) [8], *S*-*tert*-butyl 4-(diethoxyphosphono)-3-oxobutanethioate (**9**) [13] and β-d-lactose octaacetate (**12**) [14] were prepared according to literature procedures.

### 3.2. Compounds

(20-((*tert*-Butyldiphenylsilyl)oxy)eicosan-1-ol (**11**). A solution of eicosan-1,20-diol (12.2 g, 38.8 mmol) in pyridine (97 mL) was treated with TBDPSCl (1.10 mL, 42.7 mmol) and stirred for 24 h at 80 °C. H_2_O (150 mL) was added and the aqueous phase was extracted with CH_2_Cl_2_ (3 × 150 mL). The combined organic phases were dried over Na_2_SO_4_ and concentrated in vacuo. The residue was dissolved in toluene (3 × 100 mL) and concentrated in vacuo. The crude product was purified by flash chromatography (*n*-hexane/EtOAc 9:1) to give **11** (9.35 g, 16.9 mmol, 44%) as a colorless oil; *R_f_* = 0.26 (*n*-hexane/EtOAc 9:1); ^1^H NMR (CDCl_3_, 500 MHz) δ 7.66–7.70 (m, 4H), 7.35–7.44 (m, 6H), 3.73–3.78 (m, 1H), 3.62–3.68 (m, 4H), 1.84–1.88 (m, 1H), 1.52–1.61 (m, 4H), 1.22–1.39 (m, 30H), 1.05 (s, 9H); ^13^C{^1^H} NMR (CDCl_3_, 125 MHz) δ 135.6, 134.2, 129.4, 127.5, 64.0, 63.1, 32.8, 32.6, 29.69, 29.67, 29.66, 29.61, 29.59, 29.43, 29.38, 26.9, 25.8, 25.7, 25.6, 19.2; IR ν_max_ 3370, 2923, 2853, 1590, 1464, 1428, 1389, 1361, 1188, 1107, 1008, 938, 823, 738, 687, 700 cm^–1^; HRMS (ESI) *m/z* [M + H]^+^ calcd. for C_36_H_61_O_2_Si^+^, 553.44353; found 553.44366.

2′,3′,4′,6′-Tetra-*O*-acetyl-β-d-galactopyranosyl-(1→4)-(2,3,6-tri-*O*-acetyl-1-(*tert*-butyl-2-methylphenyl)thio-β-d-glucopyranoside (**13**). A solution of β-d-lactose octaacetate **12** (35.4 g, 52.1 mmol) in CH_2_Cl_2_ (130 mL) was treated with 5-*tert*-butyl-2-methylthiophenol (11.5 mL, 62.5 mmol) and BF_3_·Et_2_O (9.24 mL, 72.9 mmol), and stirred for 20 h at room temperature. 1M aqueous NaOH solution (200 mL) was added and stirring was continued for another 30 min. The aqueous phase was separated and extracted with CH_2_Cl_2_ (3 × 100 mL). The combined organic phases were dried over Na_2_SO_4_ and concentrated in vacuo. The crude product was purified by flash chromatography (*n*-hexane/EtOAc 6:4) to give 13 (35.2 g, 44.1 mmol, 85%) as a colorless foam of mp 84–85 °C; *R_f_* = 0.18 (*n*-hexane/EtOAc 7:3); [α]^24^_D_ −5.3 (*c* 0.9, CHCl_3_); ^1^H NMR (CDCl_3_, 500 MHz) δ 7.50 (d, *J* = 1.5, 1H), 7.24 (dd, *J* = 1.8, 7.9, 1H), 7.14 (d, *J* = 8.2, 1H), 5.34 (d, *J* = 2.4, 1H), 5.21 (t, *J* = 9.2, 1H), 5.09 (dd, *J* = 7.9, 10.3, 1H), 4.98 (t, *J* = 9.8, 1H), 4.94 (dd, *J* = 3.4, 10.4, 1H), 4.63 (d, *J* = 10.1, 1H), 4.47 (d, *J* = 7.9, 1H), 4.44 (d, *J* = 11.9, 1H), 4.03–4.16 (m, 3H), 3.86 (t, *J* = 6.6, 1H), 3.79 (t, *J* = 9.5, 1H), 3.56–3.64 (m, 1H), 2.35 (s, 3H), 2.14 (s, 3H), 2.09 (s, 3H), 2.09 (s, 3H), 2.04 (s, 6H), 2.02 (s, 3H), 1.95 (s, 3H), 1.31 (s, 9H); ^13^C{^1^H} NMR (CDCl_3_, 125 MHz) δ 170.3, 170.1, 170.0, 169.7, 169.6, 169.0, 149.7, 137.4, 131.4, 130.3, 130.0, 125.6, 101.1, 86.8, 76.5, 76.1, 73.9, 71.0, 70.7, 70.5, 69.0, 66.5, 62.4, 60.8, 34.4, 31.3, 20.85, 20.79, 20.77, 20.63, 20.60, 20.59, 20.5, 20.3; IR ν_max_ 2964, 1745, 1432, 1367, 1213, 1171, 1137, 1042, 954, 901, 827, 721 cm^–1^; HRMS (ESI) *m*/*z* [M + Na]^+^ calcd. for C_37_H_50_O_17_NaS^+^, 821.26609; found 821.26620.

β-d-Galactopyranosyl-(1→4)-1-(*tert*-butyl-2-methylphenyl)thio-β-d-glucopyranoside (**14**). A solution of **13** (22.4 g, 28.0 mmol) in MeOH (200 mL) was treated with NaOMe solution (3.83 mL, 25 wt% in MeOH) and stirred for 40 min at room temperature. The reaction mixture was acidified with DOWEX 50WX8-100^®^ resin, filtered, and the filtrate was concentrated in vacuo to give **14** (14.1 g, 27.9 mmol, 99%) as a colorless solid of mp 181 °C; *R_f_* = 0.09 (CH_2_Cl_2_/MeOH); [α]^24^_D_ −33.2 (*c* 0.4, DMSO); ^1^H NMR (DMSO-d_6_, 500 MHz) δ 7.47 (bs, 1H), 7.06–7.15 (m, 2H), 5.47 (d, *J* = 6.1, 1H), 5.10 (d, *J* = 4.0, 1H), 4.79–4.83 (m, 1H), 4.78 (s, 1H), 4.74 (d, *J* = 10.1, 1H), 4.61–4.68 (m, 2H), 4.53 (d, *J* = 4.6, 1H), 4.22 (d, *J* = 6.7, 1H), 3.70–3.78 (m, 1H), 3.63–3.68 (m, 1H), 3.59–3.63 (m, 1H), 3.48–3.56 (m, 2H), 3.41–3.48 (m, 3H), 3.37–3.41 (d, *J* = 9.2, 1H), 3.26–3.32 (m, 2H), 3.15–3.23 (m, 1H), 2.23 (s, 3H), 1.26 (s, 9H); ^13^C{^1^H} NMR (DMSO-d_6_, 125 MHz) δ 148.9, 133.9, 133.3, 129.4, 125.3, 122.7, 103.8, 85.9, 80.2, 78.7, 76.4, 75.5, 73.2, 72.2, 70.6, 68.1, 60.4, 60.3, 34.3, 31.1, 19.5; IR ν_max_ 3355, 2958, 2226, 2163, 1362, 1263, 1019, 873, 820, 783, 703 cm^–1^; HRMS (ESI) *m*/*z* [M + Na]^+^ calcd. for C_23_H_36_O_10_NaS^+^, 527.19214; found 527.19208.

4′,6′-Benzylidene-β-d-galactopyranosyl-(1→4)-1-(*tert*-butyl-2-methylphenyl)thio-β-d-glucopyranoside (**15**). A solution of **14** (1.00 g, 1.98 mmol) in DMF (10 mL) was treated with benzaldehyde dimethyl acetal (594 μL, 3.96 mmol) and *p* TsOH (37.7 mg, 198 μmol), and stirred for 1 h at 60 °C. NEt_3_ (100 μL) was added and the reaction mixture was concentrated in vacuo. The crude product was purified by flash chromatography (CH_2_Cl_2_/MeOH 97:3 → 94:6) to give **15** (851 mg, 1.44 mmol, 73%) as a colorless foam of mp 149 °C; *R_f_* = 0.27 (CH_2_Cl_2_/MeOH 95:5); [α]^24^_D_ −55.3 (*c* 1.6, CHCl_3_); ^1^H NMR (CDCl_3_, 500 MHz) δ 7.57 (d, *J* = 1.5), 7.44–7.50 (m, 2H), 7.28–7.38 (m, 3H), 7.21 (dd, *J* = 1.5, 7.6, 1H), 7.12 (d, *J* = 7.9), 5.45 (s, 1H), 4.68 (s, 1H), 4.49–4.56 (m, 2H), 4.45 (d, *J* = 8.2, 1H), 4.21 (d, *J* = 12.5, 1H), 4.06 (s, 1H), 3.90–4.01 (m, 2H), 3.77–3.87 (m, 2H), 3.70–3.77 (m, 1H), 3.59–3.67 (m, 2H), 3.55 (d, *J* = 7.9, 1H), 3.46 (dt, *J* = 2.1, 9.3, 1H), 3.43 (s, 1H), 3.37 (d, *J* = 9.2, 1H), 3.22–3.29 (m, 1H), 3.14–3.21 (m, 1H), 2.38 (s, 3H), 1.28 (s, 9H); ^13^C{^1^H} NMR (CDCl_3_, 125 MHz) δ 149.6, 137.2, 136.7, 131.7, 130.0, 129.6, 129.3, 128.4, 126.4, 125.0, 103.0, 101.4, 88.1, 78.6, 77.7, 76.5, 75.2, 72.31, 72.28, 69.9, 68.9, 67.0, 62.0, 34.4, 31.3, 20.4; IR ν_max_ 3405, 2871, 2345, 1488, 1453, 1363, 1264, 1218, 1165, 1076, 1024, 993, 900, 821, 734, 698 cm^–1^; HRMS (ESI) *m*/*z* [M + Na]^+^ calcd. for C_30_H_40_O_10_NaS^+^, 615.22344; found 615.22352.

4′,6′-Benzylidene-β-d-galactopyranosyl)-(1→4)-6-*O*-(*tert*-butyldiphenylsilyl)-1-(*tert*-butyl-2-methylphenyl)thio-β-d-glucopyranoside (**16**). A solution of **15** (7.65 g, 12.9 mmol) in pyridine (44 mL) was treated with TBDPSCl (9.10 mL, 35.0 mmol) and stirred for 21 h at room temperature. H_2_O (100 mL) was added, the aqueous phase was separated and extracted with CH_2_Cl_2_ (3 × 100 mL). The combined organic phases were dried over Na_2_SO_4_ and concentrated in vacuo. The residue was dissolved in toluene (3 × 40 mL) and concentrated in vacuo again. The crude product was purified by flash chromatography (*n*-hexane/EtOAc 45:55) to give 16 (6.75 g, 8.13 mmol, 63%) as a colorless foam of mp 113 °C; *R_f_* = 0.20 (*n*-hexane/EtOAc 45:55); [α]^24^_D_ −70.0 (*c* 1.2, CHCl_3_); ^1^H NMR (CDCl_3_, 500 MHz) δ 7.80 (dd, *J* = 6.7, 15.8, 4H), 7.56 (d, *J* = 1.2, 1H), 7.47–7.53 (m, 2H), 7.31–7.46 (m, 7H), 7.26 (t, *J* = 7.5, 2H), 7.21 (dd, *J* = 1.5, 7.9, 1H), 7.15 (d, *J* = 8.2, 1H), 5.52 (s, 1H), 4.60 (d, *J* = 9.8, 1H), 4.57 (d, *J* = 7.6, 1H), 4.42–4.55 (bs, 1H), 4.31 (d, *J* = 12.5, 1H), 4.12–4.20 (m, 2H), 4.02 (d, *J* = 11.9, 1H), 3.97 (d, *J* = 11.3, 1H), 3.93 (t, *J* = 9.3, 1H), 3.77 (t, *J* = 8.7, 1H), 3.73 (t, *J* = 8.7, 1H), 3.54–3.65 (m, 2H), 3.37–3.47 (m, 2H), 2.78–3.03 (m, 3H), 2.45 (s, 3H), 1.15 (s, 9H), 1.08 (s, 9H); ^13^C{^1^H} NMR (CDCl_3_, 125 MHz) δ 149.4, 137.3, 136.3, 135.9, 135.6, 133.6, 132.6, 132.5, 129.7, 129.64, 129.61, 129.3, 128.9, 128.2, 127.7, 127.6, 126.3, 124.7, 103.1, 101.2, 88.4, 79.0, 78.6, 75.9, 74.9, 72.76, 72.74, 71.3, 68.8, 66.8, 62.5, 34.3, 31.1, 26.8, 20.5, 19.4; IR ν_max_ 3426, 2957, 2858, 2171, 1979, 1733, 1590, 1488, 1460, 1428, 1392, 1362, 1263, 1158, 1084, 1026, 997, 901, 859, 821, 772, 739, 699, 669 cm^–1^; HRMS (ESI) *m*/*z* [M + Na]^+^ calcd. for C_46_H_58_O_10_NaSSi^+^, 853.34122; found 853.33926.

β-d-Galactopyranosyl-(1→4)-6-*O*-(*tert*-butyldiphenylsilyl)-1-(*tert*-butyl-2-methylphenyl)thio-β-d-glucopyranoside (**17**). A solution of **16** (20.4 g, 24.5 mmol) in CH_2_Cl_2_ (120 mL) was treated with FeCl_3_·6H_2_O (13.2 g, 49.0 mmol) and stirred for 2 h at room temperature. The reaction mixture was diluted with EtOAc (100 mL) and saturated aqueous NaHCO_3_ solution (150 mL) was added. The aqueous phase was separated and extracted with EtOAc (3 × 100 mL). The combined organic phases were dried over Na_2_SO_4_ and concentrated in vacuo. The crude product was purified by flash chromatography (CH_2_Cl_2_/MeOH 93:7) to give **17** (14.0 g, 18.8 mmol, 77%) as a colorless foam of mp 127 °C; *R_f_* = 0.32 (CH_2_Cl_2_/MeOH 9:1); [α]^24^_D_ −58.7 (*c* 1.0, CHCl_3_); ^1^H NMR (CDCl_3_, 500 MHz) δ 7.67–7.78 (m, 4H), 7.42 (s, 1H), 7.28–7.37 (m, 3H), 7.23 (d, *J* = 6.7, 1H), 7.17 (t, *J* = 6.5, 2H), 7.02 (d, *J* = 7.6, 1H), 6.99 (d, *J* = 7.3, 1H), 5.52 (bs, 1H), 4.90 (bs, 1H), 4.59–4.75 (m, 2H), 4.27–4.59 (m, 3H), 4.00–4.25 (m, 3H), 3.86–4.00 (m, 3H), 3.71–3.86 (m, 3H), 3.57–3.67 (m, 1H), 3.49 (s, 2H), 3.36 (s, 1H), 2.29 (s, 3H), 1.01 (s, 9H), 0.94 (s, 9H); ^13^C{^1^H} NMR (CDCl_3_, 125 MHz) δ 149.3, 135.8, 135.5, 133.5, 133.0, 132.6, 129.6, 129.5, 127.8, 127.6, 124.4, 102.8, 88.3, 78.9, 76.1, 74.8, 73.9, 72.6, 71.0, 69.5, 62.1, 61.9, 34.1, 31.0, 26.8, 20.4, 19.4; IR ν_max_ 3383, 2930, 2860, 2046, 1428, 1362, 1263, 1148, 1112, 1073, 1026, 895, 822, 784, 740, 701, 669 cm^–1^; HRMS (ESI) *m*/*z* [M + Na]^+^ calcd. for C_39_H_54_O_10_NaSSi^+^, 765.30992; found 765.30832.

2′,3′,4′,6′-Tetra-*O*-benzoyl-β-d-galactopyranosyl-(1→4)-2,3-di-*O*-benzoyl-6-*O*-*tert*-butyldiphenylsilyl-1-(*tert*-butyl-2-methylphenyl)thio-β-d-glucopyranoside (**18**). A solution of 17 (3.97 g, 5.34 mmol) in pyridine (14 mL) was treated with benzoyl chloride (7.44 mL, 64.1 mmol) and stirred for 18 h at room temperature. H_2_O (50 mL) was added, the aqueous phase was separated and extracted with CH_2_Cl_2_ (3 × 50 mL). The combined organic phases were dried over Na_2_SO_4_ and concentrated in vacuo. The residue was dissolved in toluene (3 × 20 mL) and concentrated in vacuo again. The crude product was purified by flash chromatography (*n*-hexane/EtOAc 8:2) to give 18 (7.28 g, 5.32 mmol, 99%) as a colorless foam of mp 107 °C; *R_f_* = 0.27 (*n*-hexane/EtOAc 8:2); [α]^24^_D_ +26.3 (*c* 1.7, CHCl_3_); ^1^H NMR (CDCl_3_, 500 MHz) δ 8.12 (d, *J* = 7.3, 2H), 8.01 (dd, *J* = 7.3, 15.3, 4H), 7.78–7.88 (m, 6H), 7.76 (d, *J* = 7.3, 2H), 7.68 (d, *J* = 7.3, 2H), 7.58–7.65 (m, 2H), 7.48–7.57 (m, 6H), 7.28–7.46 (m, 12H), 7.23 (t, *J* = 7.8, 2H), 7.19 (t, *J* = 7.8, 2H), 7.12 (q, *J* = 7.5, 3H), 7.05 (d, *J* = 7.9, 1H), 5.72–5.79 (m, 2H), 5.67 (dd, *J* = 8.1, 10.2, 1H), 5.60 (t, *J* = 9.8, 1H), 5.41 (dd, *J* = 3.2, 10.5, 1H), 5.21 (d, *J* = 7.9, 1H), 4.82 (d, *J* = 10.1, 1H), 4.65 (t, *J* = 9.8, 1H), 3.98–4.06 (m, 2H), 3.91 (q, *J* = 11.3, 2H), 3.62 (dd, *J* = 9.8, 13.4, 1H), 3.38 (d, *J* = 9.5, 1H), 2.24 (s, 3H), 1.08 (s, 9H), 1.04 (s, 9H); ^13^C{^1^H} NMR (CDCl_3_, 125 MHz) δ 165.8, 165.6, 165.4, 165.35, 165.3, 164.6, 149.5, 136.8, 136.0, 135.4, 133.6, 133.4, 133.25, 133.2, 133.1, 133.05, 132.9, 131.9, 130.3, 130.2, 130.0, 129.94, 129.89, 129.87, 129.82, 129.77, 129.75, 129.7, 129.55, 129.5, 128.9, 128.8, 128.75, 128.7, 128.5, 128.35, 128.25, 128.2, 128.0, 127.8, 125.1, 100.0, 88.0, 79.0, 74.1, 73.5, 71.8, 71.5, 71.1, 69.9, 68.0, 61.9, 61.3, 34.2, 31.0, 26.9, 20.4, 19.4; IR ν_max_ 2957, 2337, 1984, 1727, 1602, 1490, 1451, 1428, 1314, 1260, 1177, 1088, 1067, 1026, 938, 904, 822, 801, 744 cm^–1^; HRMS (ESI) *m*/*z* [M + Na]^+^ calcd. for C_81_H_78_O_16_NaSSi^+^, 1389.46720; found 1389.46721.

2′,3′,4′,6′-Tetra-*O*-benzoyl-β-d-galactopyranosyl-(1→4)-2,3-di-*O*-benzoyl-1-(*tert*-butyl-2-methylphenyl)thio-β-d-glucopyranoside (**10**). A solution of 18 (500 mg, 366 μmol) in THF (1.4 mL) was treated with TBAF solution (475 μL, 1M in THF) and stirred for 63 h at room temperature. A saturated aqueous NaHCO_3_ solution (20 mL) was added, the aqueous phase was separated and extracted with CH_2_Cl_2_ (3 × 20 mL). The combined organic phases were dried over Na_2_SO_4_ and concentrated in vacuo. The crude product was purified by flash chromatography (*n*-hexane/EtOAc 8:2 → 75:25) to give 10 (367 mg, 325 μmol, 89%) as a colorless foam of mp 115 °C; *R_f_* = 0.60 (*n*-hexane/EtOAc 7:3); [α]^24^_D_ +38.2 (*c* 1.4, CHCl_3_); ^1^H NMR (CDCl_3_, 500 MHz) δ 7.95–8.04 (m, 8H), 7.90 (d, *J* = 7.3, 2H), 7.74 (d, *J* = 7.0, 2H), 7.55–7.65 (m, 2H), 7.44–7.55 (m, 7H), 7.32–7.43 (m, 6H), 7.15–7.25 (m, 5H), 7.08 (d, *J* = 8.2, 1H), 5.74–5.81 (m, 2H), 5.70 (dd, *J* = 7.9, 10.4, 1H), 5.48–5.55 (m, 2H), 5.02 (d, *J* = 7.9, 1H), 4.87 (d, *J* = 10.1, 1H), 4.31 (t, *J* = 9.6, 1H), 4.08 (t, *J* = 6.9, 1H), 3.74–3.85 (m, 3H), 3.67 (dd, *J* = 7.3, 11.3, 1H), 3.46–3.52 (m, 1H), 2.23 (s, 3H), 1.23 (s, 9H); ^13^C{^1^H} NMR (CDCl_3_, 125 MHz) δ 165.6, 165.40, 165.39, 165.21, 165.19, 164.7, 149.6, 137.1, 133.4, 133.34, 133.29, 133.23, 133.19, 133.1, 131.8, 130.04, 130.02, 129.9, 129.8, 129.70, 129.67, 129.6, 129.4, 129.2, 129.0, 128.9, 128.6, 128.53, 128.51, 128.47, 128.3, 128.19, 128.18, 125.5, 100.9, 87.4, 78.9, 74.7, 74.1, 71.8, 71.1, 70.7, 70.0, 67.6, 60.9, 60.5, 34.3, 31.2, 20.2; IR ν_max_ 3515, 2961, 1981, 1726, 1602, 1585, 1491, 1451, 1315, 1260, 1177, 1089, 1068, 1026, 1001, 936, 853, 825, 801, 686 cm^–1^; HRMS (ESI) *m*/*z* [M + Na]^+^ calcd. for C_65_H_60_O_16_NaS^+^, 1151.34943; found 1151.35059.

2′,3′,4′,6′-Tetra-*O*-benzoyl-β-d-galactopyranosyl)-(1→4)-2,3-di-*O*-benzoyl-1-((20-*tert*-butyldiphenylsilyloxy)eicosyl-1-oxy)-β-d-glucopyranoside (**19**). A mixture of donor 10 (2.50 g, 2.21 mmol), acceptor **11** (1.47 g, 2.66 mmol) and molecular sieves (5.00 g, 4 Å) in CH_2_Cl_2_ (44 mL) was stirred for 2 h at room temperature. It was cooled to −45 °C and TMSOTf (80.0 μL, 442 μmol), BF_3_·Et_2_O (56.0 μL, 442 μmol) and NIS (1.49 g, 6.63 mmol) were added. The mixture was stirred for 1.5 h, allowing it to slowly warm to −20 °C, and filtered through Celite^®^. Saturated aqueous NaHCO_3_ solution (80 mL) was added, and the water layer was separated and extracted with CH_2_Cl_2_ (3 × 100 mL). The combined organic phases were dried over Na_2_SO_4_ and concentrated in vacuo. The crude product was purified by flash chromatography (*n*-hexane/EtOAc 8:2) to give 19 (1.88 g, 1.25 mmol, 57%) as a colorless foam of mp 79 °C; *R_f_* = 0.59 (*n*-hexane/EtOAc 7:3); [α]^24^_D_ +12.2 (*c* 0.7, CHCl_3_); ^1^H NMR (CDCl_3_, 500 MHz) δ 7.93–8.03 (m, 8H), 7.88–7.93 (m, 2H), 7.72–7.76 (m, 2H), 7.65–7.70 (m, 4H), 7.50–7.65 (m, 3H), 7.46–7.50 (m, 5H), 7.31–7.45 (m, 12H), 7.22 (t, *J* = 7.8, 2H), 7.16 (t, *J* = 7.9, 2H), 5.76–5.79 (m, 1H), 5.72–5.76 (m, 1H), 5.68–5.72 (m, 1H), 5.48 (dd, *J* = 3.4, 10.4, 1H), 5.39 (dd, *J* = 7.9, 10.1, 1H), 4.99 (d, *J* = 7.9, 1H), 4.65 (d, *J* = 7.9, 1H), 4.25 (t, *J* = 9.6, 1H), 4.07 (t, *J* = 7.0, 1H), 3.74–3.86 (m, 4H), 3.62–3.68 (m, 3H), 3.48 (dt, *J* = 2.1, 9.8, 1H), 3.42 (dt, *J* = 6.7, 9.8, 1H), 1.92 (dd, *J* = 4.6, 9.5, 1H), 1.51–1.59 (m, 2H), 1.39–1.51 (m, 2H), 1.30–1.39 (m, 2H), 1.06–1.29 (m, 30H), 1.05 (s, 9H); ^13^C{^1^H} NMR (CDCl_3_, 125 MHz) δ 165.6, 165.5, 165.4, 165.2, 165.1, 164.7, 135.6, 134.2, 133.5, 133.4, 133.3, 133.2, 133.1, 130.0, 129.8, 129.74, 129.72, 129.68, 129.65, 129.44, 129.43, 129.39, 129.01, 128.9, 128.7, 128.6, 128.54, 128.50, 128.3, 128.23, 128.18, 127.5, 101.3, 100.9, 74.94, 74.93, 72.8, 71.79, 71.76, 71.1, 70.5, 70.1, 67.6, 64.0, 61.0, 60.3, 32.6, 29.71, 29.67, 29.65, 29.6, 29.5, 29.43, 29.37, 29.35, 29.2, 26.8, 25.75, 25.73, 19.2; IR ν_max_ 3071, 2927, 2854, 1727, 1602, 1585, 1492, 1451, 1428, 1315, 1261, 1177, 1092, 1068, 1027, 1000, 936, 854, 824, 802, 742, 704, 686 cm^–1^; HRMS (ESI) *m*/*z* [M + Na]^+^ calcd. for C_90_H_104_O_18_NaSi^+^, 1523.68841; found 1523.68790.

2′,3′,4′,6′-Tetra-*O*-benzoyl-β-d-galactopyranosyl)-(1→4)-2,3-di-*O*-benzoyl-1-((20-*tert*-butyldiphenylsilyloxy)eicosyl-1-oxy)-β-d-glucuronylpyranoside (**20**). A solution of **19** (137 mg, 91.2 μmol) in CH_2_Cl_2_ (608 μL) and H_2_O (304 μL) was treated with BAIB (73.5 mg, 228 μmol) and TEMPO (5.70 mg, 36.5 μL). The reaction mixture was stirred for 2 h at room temperature. A 10% aqueous Na_2_S_2_O_3_ solution (10 mL) was added, the aqueous phase was separated and extracted with CH_2_Cl_2_ (3 × 20 mL). The combined organic phases were dried over Na_2_SO_4_ and concentrated in vacuo. The crude product was purified by flash chromatography (*n*-hexane/EtOAc 1:1 → 3:7) to give 20 (132 mg, 87.1 μmol, 96%) as a colorless foam of mp 80 °C; *R_f_* = 0.21 (*n*-hexane/EtOAc/HCO_2_H 3:7:0.1); [α]^24^_D_ −1.4 (*c* 0.6, CHCl_3_); ^1^H NMR (CDCl_3_, 500 MHz) δ 8.60–8.63 (m, 1H), 7.89–8.02 (m, 10H), 7.70–7.74 (m, 2H), 7.65–7.69 (m, 4H), 7.54–7.65 (m, 2H), 7.43–7.52 (m, 6H), 7.31–7.43 (m, 11H), 7.20 (t, *J* = 7.8, 2H), 7.14 (t, *J* = 7.8, 2H), 5.74–5.80 (m, 2H), 5.63 (dd, *J* = 7.9, 10.4, 1H), 5.51–5.57 (m, 1H), 5.45 (dd, *J* = 7.6, 9.5, 1H), 5.22 (d, *J* = 7.6, 1H), 4.74 (d, *J* = 7.6, 1H), 4.49 (t, *J* = 9.0, 1H), 4.08–4.16 (m, 2H), 3.85 (dt, *J* = 6.3, 9.8, 1H), 3.67–3.74 (m, 2H), 3.65 (t, *J* = 6.6, 2H), 3.45 (dt, *J* = 6.9, 9.8, 1H), 1.51–1.59 (m, 2H), 1.29–1.51 (m, 6H), 1.06–1.29 (m, 28H), 1.04 (s, 9H); ^13^C{^1^H} NMR (CDCl_3_, 125 MHz) δ 165.7, 165.6, 165.4, 165.3, 165.13, 165.10, 135.6, 134.2, 133.5, 133.3, 133.2, 133.10, 133.07, 133.0, 130.0, 129.74, 129.69, 129.5, 129.44, 129.36, 129.0, 128.7, 128.5, 128.3, 128.21, 128.20, 128.16, 127.5, 124.6, 101.4, 100.5, 76.4, 74.5, 72.4, 71.8, 71.6, 71.1, 70.5, 70.2, 67.9, 64.0, 61.2, 32.6, 29.71, 29.67, 29.66, 29.64, 29.61, 29.5, 29.45, 29.4, 29.3, 26.8, 25.75, 25.7, 19.2; IR ν_max_ 2926, 2854, 1727, 1602, 1585, 1451, 1428, 1315, 1262, 1177, 1091, 1068, 1026, 1000, 936, 854, 823, 802, 742, 704, 686 cm^–1^; HRMS (ESI) *m*/*z* [M + H]^+^ calcd. for C_90_H_103_O_9_Si^+^, 1515.68573; found 1515.68235.

2′,3′,4′,6′-Tetra-*O*-benzoyl-β-d-galactopyranosyl)-(1→4)-2,3-di-*O*-benzoyl-5-benzoxycarbonyl-1-((20-*tert*-butyldiphenylsilyloxy)eicosyl-1-oxy)-β-d-glucopyranoside (**21**). A mixture of **20** (214 mg, 141 μmol), K_2_CO_3_ (23.4 mg, 169 μmol) and benzyl bromide (21.7 μL, 183 μmol) in DMF (1.4 mL) was stirred for 2 h at room temperature. Saturated aqueous NH_4_Cl solution (10 mL) was added, the aqueous phase was separated and extracted with EtOAc (3 × 20 mL). The combined organic phases were dried over Na_2_SO_4_ and concentrated in vacuo. The crude product was purified by flash chromatography (*n*-hexane/EtOAc 85:15) to give **21** (213 mg, 133 μmol, 94%) as a colorless foam of mp 61 °C; *R_f_* = 0.31 (*n*-hexane/EtOAc 8:2); [α]^24^_D_ +2.2 (*c* 0.8, CHCl_3_); ^1^H NMR (CDCl_3_, 500 MHz) δ 8.07 (d, *J* = 8.2, 2H), 7.95–8.03 (m, 6H), 7.91 (d, *J* = 7.9, 2H), 7.74–7.78 (m, 2H), 7.68–7.72 (m, 4H), 7.59–7.65 (m, 2H), 7.46–7.56 (m, 6H), 7.30–7.46 (m, 17H), 7.23 (t, *J* = 7.9, 2H), 7.14 (t, *J* = 7.6, 2H), 5.77 (t, *J* = 9.5, 1H), 5.69 (d, *J* = 3.4, 1H), 5.55–5.61 (m, 1H), 5.43–5.49 (m, 1H), 5.37 (d, *J* = 12.2, 1H), 5.31 (dd, *J* = 3.4, 10.4, 1H), 4.69–4.75 (m, 3H), 4.45 (t, *J* = 9.3, 1H), 4.13 (dd, *J* = 0.8, 9.7, 1H), 3.83 (dt, *J* = 6.3, 9.7, 1H), 3.62–3.76 (m, 5H), 3.47 (dt, *J* = 6.7, 9.7, 1H), 1.54–1.62 (m, 2H), 1.42–1.54 (m, 2H), 1.33–1.41 (m, 2H), 1.09–1.33 (m, 30H), 1.07 (s, 9H); ^13^C{^1^H} NMR (CDCl_3_, 125 MHz) δ 166.9, 165.6, 165.4, 165.24, 165.19, 165.0, 164.9, 135.5, 134.9, 134.1, 133.4, 133.3, 133.2, 133.1, 133.0, 129.9, 129.8, 129.69, 129.66, 129.63, 129.59, 129.41, 129.40, 129.3, 128.9, 128.83, 128.81, 128.76, 128.7, 128.6, 128.5, 128.3, 128.2, 128.1, 127.5, 101.5, 100.3, 76.0, 74.6, 72.3, 71.6, 71.5, 71.0, 70.5, 69.8, 67.6, 67.2, 63.9, 61.1, 32.5, 29.7, 29.64, 29.62, 29.60, 29.57, 29.5, 29.4, 29.3, 29.23, 29.20, 26.8, 25.71, 25.67, 19.2; IR ν_max_ 3070, 2925, 2854, 1728, 1602, 1585, 1492, 1451, 1428, 1315, 1260, 1177, 1090, 1067, 1026, 937, 854, 822, 801, 742, 704, 686 cm^–1^; HRMS (ESI) *m*/*z* [M + Na]^+^ calcd. for C_97_H_108_O_19_NaSi^+^, 1627.71463; found 1627.71130.

2′,3′,4′,6′-Tetra-*O*-benzoyl-β-d-galactopyranosyl)-(1→4)-2,3-di-*O*-benzoyl-5-benzoxycarbonyl-1-((20-hydroxyeicosyl-1-oxy)-β-d-glucopyranoside (**22**). A solution of **21** (209 mg, 130 μmol) in THF (1.1 mL) was treated with TBAF solution (156 μL, 1M in THF) and stirred for 19 h at room temperature. Saturated aqueous NaHCO_3_ solution (10 mL) was added, the aqueous phase was separated and extracted with CH_2_Cl_2_ (3 × 20 mL). The combined organic phases were dried over Na_2_SO_4_ and concentrated in vacuo. The crude product was purified by flash chromatography (*n*-hexane/EtOAc 9:1 → 7:3) to give **22** (139 mg, 102 μmol, 78%) as a colorless foam of mp 66 °C; *R_f_* = 0.29 (*n*-hexane/EtOAc 7:3); [α]^24^_D_ +2.5 (*c* 0.4, CHCl_3_); ^1^H NMR (CDCl_3_, 500 MHz) δ 8.02–8.05 (m, 2H), 7.92–8.00 (m, 6H), 7.85–7.90 (m, 2H), 7.70–7.75 (m, 2H), 7.58–7.64 (m, 2H), 7.44–7.54 (m, 6H), 7.27–7.44 (m, 11H), 7.22 (t, *J* = 7.6, 2H), 7.12 (t, *J* = 7.6, 2H), 5.73 (t, *J* = 9.5, 1H), 5.65 (d, *J* = 3.4, 1H), 5.54 (dd, *J* = 7.6, 10.4, 1H), 5.42 (dd, *J* = 7.6, 9.8, 1H), 5.33 (d, *J* = 11.9, 1H), 5.27 (dd, *J* = 3.4, 10.4, 1H), 4.66–4.71 (m, 3H), 4.41 (t, *J* = 9.3, 1H), 4.09 (d, *J* = 9.8, 1H), 3.80 (dt, *J* = 6.4, 9.8, 1H), 3.69 (t, *J* = 6.7, 1H), 3.59–3.67 (m, 4H), 3.43 (dt, *J* = 6.7, 9.8, 1H), 1.52–1.60 (m, 2H), 1.40–1.52 (m, 2H), 0.98–1.39 (m, 32H); ^13^C{^1^H} NMR (CDCl_3_, 125 MHz) δ 166.9, 165.6, 165.4, 165.3, 165.2, 165.1, 164.9, 134.9, 133.4, 133.3, 133.2, 133.1, 133.05, 133.0, 129.8, 129.75, 129.7, 129.65, 129.6, 129.45, 129.4, 129.3, 128.9, 128.85, 128.8, 128.75, 128.7, 128.6, 128.5, 128.3, 128.2, 128.1, 101.5, 100.3, 76.0, 74.6, 72.3, 71.6, 71.5, 71.0, 70.5, 69.8, 67.6, 67.3, 63.0, 61.1, 32.8, 29.7, 29.64, 29.63, 29.61, 29.60, 29.58, 29.56, 29.5, 29.42, 29.41, 29.3, 29.2, 25.7; IR ν_max_ 2926, 2854, 2254, 1728, 1602, 1585, 1493, 1452, 1315, 1263, 1177, 1092, 1068, 1027, 1001, 908, 856, 802, 730, 706 cm^–1^; HRMS (ESI) *m*/*z* [M + H]^+^ calcd. for C_81_H_91_O_19_^+^, 1367.61491; found 1367.61305.

2′,3′,4′,6′-Tetra-*O*-benzoyl-β-d-galactopyranosyl)-(1→4)-2,3-di-*O*-benzoyl-5-benzoxycarbonyl-1-((20-oxoeicosyl-1-oxy)-β-d-glucopyranoside (**8**). A solution of **22** (1.00 g, 731 μmol) in CH_2_Cl_2_ (14.6 mL) was treated with DMP (467 mg, 1.10 mmol) and the resulting mixture was stirred for 2 h at room temperature. A 10% aqueous Na_2_S_2_O_3_ solution (50 mL) was added, the aqueous phase was separated and extracted with CH_2_Cl_2_ (3 × 50 mL). The combined organic phases were dried over Na_2_SO_4_ and concentrated in vacuo. The crude product was purified by flash chromatography (*n*-hexane/EtOAc 82:18) to give **8** (890 mg, 652 μmol, 89%) as a colorless foam of mp 58 °C; *R_f_* = 0.29 (*n*-hexane/EtOAc 8:2); [α]^24^_D_ −5.8 (*c* 0.3, CHCl_3_); ^1^H NMR (CDCl_3_, 500 MHz) δ 9.76 (t, *J* = 2.0, 1H), 8.02–8.05 (m, 2H), 7.92–8.00 (m, 6H), 7.85–7.90 (m, 2H), 7.70–7.75 (m, 2H), 7.58–7.64 (m, 2H), 7.46–7.56 (m, 6H), 7.27–7.46 (m, 11H), 7.22 (t, *J* = 7.6, 2H), 7.12 (t, *J* = 7.6, 2H), 5.73 (t, *J* = 9.3, 1H), 5.65 (d, *J* = 3.7, 1H), 5.54 (dd, *J* = 7.9, 10.4, 1H), 5.42 (dd, *J* = 7.6, 9.8, 1H), 5.33 (d, *J* = 11.9, 1H), 5.27 (dd, *J* = 3.4, 10.4, 1H), 4.66–4.71 (m, 3H), 4.41 (t, *J* = 9.3, 1H), 4.09 (d, *J* = 9.5, 1H), 3.80 (dt, *J* = 6.4, 9.8, 1H), 3.67–3.72 (m, 1H), 3.59–3.66 (m, 2H), 3.43 (dt, *J* = 6.7, 9.8, 1H), 2.42 (dt, *J* = 1.8, 7.3, 2H), 1.62 (q, *J* = 7.2, 2H), 1.39–1.50 (m, 2H), 0.98–1.35 (m, 32H); ^13^C{^1^H} NMR (CDCl_3_, 125 MHz) δ 203.3, 166.9, 165.6, 165.4, 165.3, 165.2, 165.1, 165.0, 134.9, 133.5, 133.4, 133.2, 133.15, 133.0, 130.0, 129.8, 129.75, 129.70, 129.68, 129.6, 129.46, 129.44, 129.3, 128.89, 128.87, 128.85, 128.80, 128.7, 128.6, 128.5, 128.3, 128.24, 128.15, 100.6, 100.3, 76.0, 74.6, 72.3, 71.6, 71.5, 71.0, 70.6, 69.8, 67.6, 67.3, 61.1, 43.9, 33.4, 29.69, 29.67, 29.66, 29.64, 29.58, 29.54, 29.45, 29.42, 29.35, 29.28, 29.25, 29.23, 29.15, 29.1, 25.7, 24.7; IR ν_max_ 3064, 2924, 2853, 1726, 1602, 1585, 1493, 1451, 1315, 1261, 1177, 1091, 1067, 1026, 1001, 911, 855, 802, 746, 687 cm^–1^; HRMS (ESI) *m*/*z* [M + Na]^+^ calcd. for C_81_H_88_O_19_Na^+^, 1387.58120; found 1387.58412.

2′,3′,4′,6′-Tetra-*O*-benzoyl-β-d-galactopyranosyl)-(1→4)-2,3-di-*O*-benzoyl-5-benzoxycarbonyl-1-(22-oxo-24-(*S*-*tert*-butylthiocarbonyl)-eicosa-20-enyl-1-oxy)-β-d-glucopyranoside (**6**). To a solution of phosphonate **9** (34.1 mg, 110 μmol) in THF (1.35 mL) at 0 °C was dropwise added a LiHMDS solution (220 μL, 1M in THF) and the resulting mixture was stirred for 30 min at room temperature, cooled to 0 °C, and treated with a solution of **8** (100 mg, 73.2 μmol) in THF (1.35 mL). After stirring for another 16 h at room temperature, a saturated aqueous NH_4_Cl solution (20 mL) was added. The aqueous layer was separated and extracted with Et_2_O (3 × 20 mL). The combined organic phases were dried over Na_2_SO_4_ and concentrated in vacuo. The crude product was purified by flash chromatography (*n*-hexane/EtOAc 85:15) to give 6 (78.0 mg, 51.3 μmol, 70%) as a colorless foam of mp 56 °C and as 1:1.2 mixture of keto/enol tautomers (as to NMR); *R_f_* = 0.45 (*n*-hexane/EtOAc 8:2); [α]^24^_D_ +1.2 (*c* 0.6, CHCl_3_); ^1^H NMR (CDCl_3_, 500 MHz) of the *keto tautomer*: δ 8.01–8.06 (m, 2H), 7.91–8.00 (m, 6H), 7.84–7.89 (m, 2H), 7.70–7.75 (m, 2H), 7.58–7.65 (m, 2H), 7.45–7.54 (m, 6H), 7.27–7.43 (m, 11H), 7.22 (t, *J* = 7.8, 2H), 7.12 (t, *J* = 7.8, 2H), 6.91 (dt, *J* = 7.0, 15.9, 1H), 6.15 (dt, *J* = 1.4, 15.9, 1H), 5.73 (t, *J* = 9.5, 1H), 5.65 (d, *J* = 3.7, 1H), 5.53 (dd, *J* = 7.9, 10.4, 1H), 5.42 (dd, *J* = 7.5, 9.6, 1H), 5.33 (d, *J* = 12.2, 1H), 5.27 (dd, *J* = 3.4, 10.4, 1H), 4.65–4.70 (m, 3H), 4.40 (t, *J* = 9.5, 1H), 4.09 (d, *J* = 9.8, 1H), 3.80 (dt, *J* = 6.3, 9.8, 1H), 3.70 (bs, 2H), 3.68 (d, *J* = 6.7, 1H), 3.61–3.65 (m, 2H), 3.43 (dt, *J* = 6.7, 9.8, 1H), 2.23 (q, *J* = 6.7, 1H), 2.17 (q, *J* = 6.7, 1H), 1.51 (s, 4.5H), 1.47 (s, 5.5H), 1.40–1.45 (m, 2H), 1.05–1.35 (m, 32H); ^1^H NMR (CDCl_3_, 500 MHz) of the *enol tautomer*: δ 12.62 (d, *J* = 1.5, 1H), 8.01–8.06 (m, 2H), 7.91–8.00 (m, 6H), 7.84–7.89 (m, 2H), 7.70–7.75 (m, 2H), 7.58–7.65 (m, 2H), 7.45–7.54 (m, 6H), 7.27–7.43 (m, 11H), 7.22 (t, *J* = 7.8, 2H), 7.12 (t, *J* = 7.8, 2H), 6.70 (dt, *J* = 7.0, 15.6, 1H), 5.73 (t, *J* = 9.5, 1H), 5.68 (s, 1H), 5.65 (d, *J* = 3.7, 1H), 5.53 (dd, *J* = 7.9, 10.4, 1H), 5.42 (dd, *J* = 7.5, 9.6, 1H), 5.33 (d, *J* = 12.2, 1H), 5.31 (s, 1H), 5.27 (dd, *J* = 3.4, 10.4, 1H), 4.65–4.70 (m, 3H), 4.40 (t, *J* = 9.5, 1H), 4.09 (d, *J* = 9.8, 1H), 3.80 (dt, *J* = 6.3, 9.8, 1H), 3.68 (d, *J* = 6.7, 1H), 3.61–3.65 (m, 2H), 3.43 (dt, *J* = 6.7, 9.8, 1H), 2.23 (q, *J* = 6.7, 1H), 2.17 (q, *J* = 6.7, 1H), 1.51 (s, 4.5H), 1.47 (s, 5.5H), 1.40–1.45 (m, 2H), 1.05–1.35 (m, 32H); ^13^C{^1^H} NMR (CDCl_3_, 125 MHz) δ 196.4, 192.7, 191.8, 166.9, 166.7, 165.6, 165.4, 165.3, 165.2, 165.1, 165.0, 150.8, 142.8, 134.9, 133.5, 133.4, 133.2, 133.11, 133.07, 130.1, 130.0, 129.8, 129.72, 129.69, 129.66, 129.6, 129.5, 129.44, 129.42, 129.3, 128.87, 128.86, 128.83, 128.77, 128.7, 128.6, 128.5, 128.31, 128.29, 128.2, 128.1, 124.0, 101.5, 100.3, 100.2, 76.0, 74.6, 72.3, 71.6, 71.5, 71.0, 70.6, 69.8, 67.6, 67.3, 61.1, 56.1, 48.9, 48.2, 32.7, 32.6, 30.1, 29.69, 29.67, 29.65, 29.62, 29.59, 29.53, 29.51, 29.49, 29.43, 29.40, 29.36, 29.26, 29.23, 29.15, 29.1, 28.4, 27.9, 25.7; IR ν_max_ 3446, 2925, 2854, 2357, 1729, 1655, 1602, 1585, 1452, 1365, 1316, 1261, 1177, 1091, 1069, 1026, 907, 858, 801, 729, 706 cm^–1^; HRMS (ESI) *m*/*z* [M + Na]^+^ calcd. for C_89_H_100_O_20_NaS^+^, 1543.64209; found 1543.63997.

4-Benzyl 1-methyl *N*-(*E*)-24-[((2′′,3′′,4′′,6′′-tetra-*O*-benzoyl-β-d-galactopyranosyl)-(1→4)-2′,3′-di-*O*-benzoyl-5′-benzoxycarbonyl-β-d-glucopyranosid-1′-yloxy))-tetraeicos-4-enoyl]-*N*-methyl-d-aspartate (**23**). A mixture of **6** (500 mg, 329 μmol), **7** (99.0 mg, 394 μmol) and NEt_3_ (91.6 μL, 657 μmol) in THF (16 mL) was treated with AgO_2_CCF_3_ (87.0 mg, 394 μmol) at 0 °C and stirred for 3 h at 0 °C. Saturated aqueous NH_4_Cl solution (30 mL) was added, the aqueous phase was separated and extracted with Et_2_O (3 × 30 mL). The combined organic phases were dried over Na_2_SO_4_ and concentrated in vacuo. The crude product was purified by flash chromatography (*n*-hexane/EtOAc 7:3) to give **23** (468 mg, 278 μmol, 85%) as a colorless foam of mp 63 °C and as a 1:3 mixture of keto/enol tautomers; *R_f_* = 0.26 (*n*-hexane/EtOAc 7:3); [α]^24^_D_ +14.4 (*c* 0.7, CHCl_3_); ^1^H NMR (CDCl_3_, 500 MHz) of the *keto tautomer*: δ 8.02–8.06 (m, 2H), 7.92–8.00 (m, 6H), 7.85–7.90 (m, 2H), 7.71–7.75 (m, 2H), 7.58–7.64 (m, 2H), 7.44–7.54 (m, 6H), 7.27–7.43 (m, 16H), 7.22 (t, *J* = 7.8, 2H), 7.12 (t, *J* = 7.9, 2H), 6.91–6.99 (m, 1H), 6.12–6.18 (m, 1H), 5.73 (t, *J* = 9.5, 1H), 5.65 (d, *J* = 3.4, 1H), 5.54 (dd, *J* = 7.8, 10.2, 1H), 5.42 (dd, *J* = 7.5, 9.6, 1H), 5.33 (d, *J* = 12.2, 1H), 5.27 (dd, *J* = 3.4, 10.4, 1H), 5.12–5.15 (m, 2H), 4.90 (dd, *J* = 6.3, 7.8, 1H), 4.66–4.71 (m, 3H), 4.41 (t, *J* = 9.3, 1H), 4.09 (d, *J* = 9.8, 1H), 3.80 (dt, *J* = 6.4, 9.8, 1H), 3.68–3.71 (m, 4H), 3.60–3.65 (m, 4H), 3.43 (dt, *J* = 6.7, 9.8, 1H), 3.20 (dt, *J* = 5.6, 16.5, 1H), 3.02 (s, 3H), 2.89 (ddd, *J* = 1.5, 8.5, 16.5, 1H), 2.14–2.25 (m, 2H), 1.58–1.63 (m, 2H), 1.40–1.49 (m, 4H), 1.05–1.35 (m, 30H); ^1^H NMR (CDCl_3_, 500 MHz) of the *enol tautomer*: δ 14.00 (d, *J* = 0.9, 1H), 8.02–8.06 (m, 2H), 7.92–8.00 (m, 6H), 7.85–7.90 (m, 2H), 7.71–7.75 (m, 2H), 7.58–7.64 (m, 2H), 7.44–7.54 (m, 6H), 7.27–7.43 (m, 16H), 7.22 (t, *J* = 7.8, 2H), 7.12 (t, *J* = 7.9, 2H), 6.65 (dt, *J* = 7.4, 15.3, 1H), 5.79 (dd, *J* = 1.1, 15.4, 1H), 5.73 (t, *J* = 9.5, 1H), 5.65 (d, *J* = 3.4, 1H), 5.54 (dd, *J* = 7.8, 10.2, 1H), 5.42 (dd, *J* = 7.5, 9.6, 1H), 5.33 (d, *J* = 12.2, 1H), 5.27 (dd, *J* = 3.4, 10.4, 1H), 5.12–5.15 (m, 2H), 5.07 (s, 1H), 4.98–5.04 (m, 1H), 4.66–4.71 (m, 3H), 4.41 (t, *J* = 9.3, 1H), 4.09 (d, *J* = 9.8, 1H), 3.80 (dt, *J* = 6.4, 9.8, 1H), 3.68–3.71 (m, 4H), 3.60–3.65 (m, 2H), 3.43 (dt, *J* = 6.7, 9.8, 1H), 3.20 (dt, *J* = 5.6, 16.5, 1H), 2.99 (s, 3H), 2.89 (ddd, *J* = 1.5, 8.5, 16.5, 1H), 2.14–2.25 (m, 2H), 1.58–1.63 (m, 2H), 1.40–1.49 (m, 4H), 1.05–1.35 (m, 30H); ^13^C{^1^H} NMR (CDCl_3_, 125 MHz) δ 192.9, 172.5, 170.7, 170.5, 170.1, 168.7, 167.5, 166.9, 165.6, 165.4, 165.3, 165.2, 165.1, 164.9, 150.5, 140.6, 134.9, 133.5, 133.4, 133.19, 133.18, 133.11, 133.06, 130.0, 129.8, 129.73, 129.70, 129.67, 129.6, 129.5, 129.4, 129.3, 129.2, 128.89, 128.86, 128.84, 128.77, 128.7, 128.62, 128.55, 128.5, 128.3, 128.2, 128.1, 125.1, 101.5, 100.3, 87.7, 76.0, 74.6, 72.3, 71.6, 71.5, 71.0, 70.6, 69.8, 67.6, 67.3, 66.7, 61.1, 56.9, 56.1, 52.6, 52.5, 47.4, 36.0, 34.8, 34.6, 34.2, 32.61, 32.57, 29.71, 29.68, 29.66, 29.64, 29.57, 29.54, 29.46, 29.45, 29.38, 29.27, 29.24, 29.22, 29.21, 28.6, 28.0, 25.7, 23.8; IR ν_max_ 2925, 2854, 1730, 1656, 1601, 1492, 1452, 1374, 1315, 1264, 1177, 1093, 1069, 1027, 910, 852, 803, 708 cm^–1^; HRMS (ESI) *m*/*z* [M + H]^+^ calcd. for C_98_H_108_O_24_N^+^, 1682.72558; found 1682.72416.

4-Benzyl 1-methyl *N*-(*E*)-24-[((2′′,3′′,4′′,6′′-tetra-*O*-benzoyl-β-d-galactopyranosyl)-(1→4)-2′,3′-di-*O*-benzoyl-5′-carboxyl-β-d-glucopyranosid-1′-yloxy))-tetraeicosanoyl]-*N*-methyl-d-aspartate (**5**). A solution of **23** (240 mg, 143 μmol) in EtOAc (7 mL) was treated with 10% Pd/C (120 mg, 50 wt%). The resulting mixture was stirred under a hydrogen atmosphere for 19 h at room temperature and then filtered through Celite^®^ to give **5** (200 mg, 133 μmol, 93%) as a colorless foam of mp 105 °C and as a 1:4 mixture of keto and enol tautomers; *R_f_* = 0.19 (CH_2_Cl_2_/MeOH/HCO_2_H 95:5:0.1); [α]^24^_D_ +12.3 (*c* 0.6, CHCl_3_); ^1^H NMR (CDCl_3_, 500 MHz) of the *keto tautomer*: δ 7.93–8.02 (m, 8H), 7.91 (d, *J* = 7.6, 2H), 7.71 (d, *J* = 7.6, 2H), 7.53–7.65 (m, 2H), 7.43–7.51 (m, 5H), 7.28–7.43 (m, 7H), 7.18 (t, *J* = 7.8, 2H), 7.13 (t, *J* = 7.8, 2H), 5.73–5.81 (m, 2H), 5.57–5.69 (m, 2H), 5.41–5.49 (m, 1H), 5.19 (d, *J* = 7.3, 1H), 4.88–5.02 (m, 1H), 4.74 (d, *J* = 7.3, 1H), 4.47 (t, *J* = 9.2, 1H), 4.20 (t, *J* = 6.3, 1H), 4.11 (d, *J* = 9.5, 1H), 3.84 (dt, *J* = 6.4, 9.5, 1H), 3.78–3.83 (m, 2H), 3.71–3.78 (m, 3H), 3.63–3.71 (m, 2H), 3.45 (dt, *J* = 6.7, 9.5, 1H), 3.23 (dd, *J* = 6.1, 7.1, 1H), 2.91 (dd, *J* = 7.9, 17.1, 1H), 2.80–2.87 (s, 3H), 2.48–2.58 (m, 2H), 1.51–1.61 (m, 2H), 1.39–1.51 (m, 2H), 1.05–1.35 (m, 34H); ^1^H NMR (CDCl_3_, 500 MHz) of the *enol tautomer*: δ 7.93–8.02 (m, 8H), 7.91 (d, *J* = 7.6, 2H), 7.71 (d, *J* = 7.6, 2H), 7.53–7.65 (m, 2H), 7.43–7.51 (m, 5H), 7.28–7.43 (m, 7H), 7.18 (t, *J* = 7.8, 2H), 7.13 (t, *J* = 7.8, 2H), 5.73–5.81 (m, 2H), 5.57–5.69 (m, 2H), 5.41–5.49 (m, 1H), 5.19 (d, *J* = 7.3, 1H), 4.88–5.02 (m, 1H), 4.74 (d, *J* = 7.3, 1H), 4.47 (t, *J* = 9.2, 1H), 4.20 (t, *J* = 6.3, 1H), 4.11 (d, *J* = 9.5, 1H), 3.84 (dt, *J* = 6.4, 9.5, 1H), 3.71–3.78 (m, 3H), 3.63–3.71 (m, 2H), 3.60 (s, 1H), 3.45 (dt, *J* = 6.7, 9.5, 1H), 3.23 (dd, *J* = 6.1, 7.1, 1H), 3.00–3.08 (m, 3H), 2.91 (dd, *J* = 7.9, 17.1, 1H), 2.48–2.58 (m, 2H), 1.51–1.61 (m, 2H), 1.39–1.51 (m, 2H), 1.05–1.35 (m, 34H); ^13^C{^1^H} NMR (CDCl_3_, 125 MHz) δ 204.9, 204.2, 179.6, 174.9, 172.3, 170.9, 170.6, 169.8, 169.6, 168.1, 165.7, 165.6, 165.4, 165.3, 165.2, 165.1, 133.4, 133.23, 133.20, 133.12, 133.10, 129.9, 129.7, 129.64, 129.62, 129.5, 129.4, 129.23, 129.20, 128.9, 128.6, 128.5, 128.26, 128.25, 128.2, 128.1, 101.4, 100.3, 76.1, 74.0, 72.3, 71.7, 71.4, 71.0, 70.5, 70.3, 67.9, 61.2, 60.5, 57.3, 56.7, 53.0, 52.7, 49.1, 48.9, 43.2, 43.0, 35.9, 33.8, 29.61, 29.58, 29.54, 29.48, 29.43, 29.38, 29.35, 29.32, 29.28, 29.2, 28.97, 28.95, 25.6, 23.4, 21.0, 14.1; IR ν_max_ 2925, 2853, 1726, 1602, 1585, 1492, 1451, 1315, 1263, 1177, 1092, 1068, 1026, 1000, 936, 854, 803, 706, 686 cm^–1^; HRMS (ESI) *m*/*z* [M + H]^+^ calcd. for C_84_H_98_O_24_N^+^, 1504.64733; found 1504.64872.

Ancorinoside B (**2**). A solution of **5** (129 mg, 85.7 μmol) in MeOH (8.6 mL) was treated with NaOMe solution (117 μL, 25 wt% in MeOH) and stirred for 30 min at 50 °C. The reaction mixture was cooled to room temperature, acidified with DOWEX 50WX8-100^®^ resin and filtered. The solution was washed with heptane (25 mL) and concentrated in vacuo. The residue was recrystallized from MeOH to give **2** (56.0 mg, 66.0 μmol, 77%) as a beige solid of mp 101 °C; [α]^24^_D_ +3.2 (*c* 0.1, MeOH); ^1^H NMR (CDCl_3_, 500 MHz) δ 4.37 (d, *J* = 7.3, 1H), 4.35 (d, *J* = 7.6, 1H), 4.04–4.08 (m, 1H), 3.97 (d, *J* = 9.8, 1H), 3.85 (dt, *J* = 6.7, 9.5, 1H), 3.75–3.81 (m, 3H), 3.69 (dd, *J* = 4.6, 11.3, 1H), 3.54–3.59 (m, 3H), 3.52 (d, *J* = 7.3, 1H), 3.47 (dd, *J* = 3.1, 9.8, 1H), 3.26–3.29 (m, 1H), 2.96 (s, 3H), 2.91 (dd, *J* = 4.7, 17.0), 2.78–2.87 (m, 3H), 1.64–1.70 (m, 2H), 1.57–1.64 (m, 2H), 1.24–1.44 (m, 34H); ^13^C{^1^H} NMR (CDCl_3_, 125 MHz) δ 178.2, 173.2, 172.1, 104.9, 104.7, 81.4, 77.3, 76.2, 75.2, 74.8, 74.5, 72.6, 71.4, 70.4, 68.3, 62.6, 34.6, 33.4, 30.91, 30.89, 30.85, 30.8, 30.72, 30.68, 30.5, 30.4, 27.4, 27.21, 27.19, 27.0; IR ν_max_ 3349, 2920, 2851, 2204, 2033, 1725, 1628, 1495, 1470, 1425, 1249, 1162, 1072, 1033, 951, 914, 770, 718, 635 cm^–1^; HRMS (ESI) *m*/*z* [M − H]^−^ calcd. for C_41_H_68_O_17_N^–^, 846.44818; found 846.44967.

Ancorinoside B tris(diethylammonium) salt. A solution of **2** (15.0 mg, 17.7 μmol) in MeOH (885 μL) was treated with HNEt_2_ (11 μL, 106 μmol) and stirred for 1 h at room temperature. The reaction mixture was concentrated in vacuo to give the ammonium salt (18.7 mg, 17.5 μmol, 99%) as a brown solid of mp 57 °C; [α]^24^_D_ +5.0 (*c* 0.1, MeOH); ^1^H NMR (CDCl_3_, 500 MHz) δ 4.35 (d, *J* = 7.3, 1H), 4.30 (d, *J* = 7.9, 1H), 3.90–3.95 (m, 1H), 3.82–3.89 (m, 1H), 3.77–3.79 (m, 1H), 3.73–3.77 (m, 1H), 3.69–3.73 (m, 1H), 3.66–3.69 (m, 1H), 3.59–3.62 (m, 1H), 3.56–3.59 (m, 1H), 3.54–3.56 (m, 1H), 3.52–3.54 (m, 1H), 3.49–3.52 (m, 1H), 3.48 (dd, *J* = 3.5, 9.9, 1H), 3.27–3.29 (m, 1H), 3.02 (q, *J* = 7.0, 12H), 2.91 (s, 3H), 2.80–2.84 (m, 1H), 2.76–2.80 (m, 1H), 2.70–2.76 (m, 1H), 2.13 (dd, *J* = 9.2, 15.3), 1.58–1.66 (m, 2H), 1.51–1.58 (m, 2H), 1.24–1.43 (m, 54H); ^13^C{^1^H} NMR (CDCl_3_, 125 MHz) δ 197.8, 196.7, 179.9, 176.5, 176.1, 105.6, 104.3, 102.4, 83.6, 77.4, 77.3, 76.8, 75.1, 74.7, 72.8, 71.2, 70.4, 64.7, 62.6, 43.6, 41.7, 41.1, 31.2, 31.0, 30.95, 30.90, 30.8, 30.7, 30.6, 27.8, 27.6, 27.3, 27.2, 11.9; IR ν_max_ 3375, 2920, 2851, 2510, 2323, 2049, 1591, 1456, 1394, 1258, 1160, 1053, 784, 672 cm^–1^; HRMS (ESI) *m*/*z* [M − H]^−^ calcd. for C_53_H_103_O_17_N_4_^–^, 1067.73127; found 1067.73446.

### 3.3. Antimicrobial Assay

Minimum Inhibitory Concentrations (MIC) of ancorinoside B (**2**) were ascertained by serial dilution assays as reported previously [15,16] employing the following microorganisms as provided by the DSMZ-German Collection of Microorganisms and Cell Cultures GmbH, (Braunschweig, Germany) or the ATCC-American Type Culture Collection (Manassas, VA, USA): *Pichia anomala*, *Schizosaccharomyces pombe*, *Mucor hiemalis*, *Candida albicans*, and *Rhodotorula glutinis* for fungal microorganisms; *Bacillus subtilis*, *Staphyloccocus aureus* and *Mycobacterium smegmatis* for Gram-positive bacteria, *Acinetobacter baumannii*, *Chromobacterium violaceum*, *Escherichia coli* and *Pseudomonas aeruginosa* for Gram-negative bacteria. For accession numbers *cf* Table 1.

### 3.4. Inhibition of Biofilm Formation

*Staphylococcus aureus* DSM 1104 samples were taken from a –20 °C stock and incubated overnight in 25 mL CASO (casein-peptone soymeal-peptone) medium at 37 °C while shaking (100 rpm). The OD_600_ of the culture solution was adjusted to match the turbidity of a 0.001 McFarland standard. 150 µL of CASO with 4% glucose broth was added as well as serially diluted compounds (250–0.13 µg/mL) and incubated in 96 well microtiter plates (TPP tissue culture ref. no. 92196) at 37 °C for 18 h. Any resulting biofilm inhibition was evaluated via staining with 0.1% crystal violet (Thermo Fisher, Waltham, MA, USA) following established protocols [12,17] In brief, the supernatant was discarded, the biofilm stained at room temperature with 0.1% crystal violet for 15 min and washed thrice with PBS (phosphate-buffered saline) buffer. The dye in the biofilm was extracted with 30% acetic acid, and the absorbance was quantified with a plate reader (Synergy 2, BioTek, Santa Clara, CA, USA) at 550 nm. DMSO (2.5%) served as a negative control and microporenic acid A [12] (250–0.13 µg/mL) as a positive control. All experiments were run in duplicates with two repetitions. SD of two repeats with duplicates each were 10% or less.

*P. aeruginosa* (PA 14) DSM 19882 was grown in 25 mL LB medium (Luria-Bertani Broth) in a 250 mL flask at 37 °C, shaking at 100 rpm for 18 h. The OD_600_ of the culture solution was adjusted to 0.1 McFarland standard in M63 medium, supplemented with magnesium sulfate, glucose, and casamino acids as previously described [18]. The compounds were added to 150 μL bacterial solution at various concentrations (250–2 μg/mL), then the solution was added to U-bottom 96-well plates (Falcon non-tissue plate with U-bottom ref. no. 351177). The plates were incubated at 37 °C at 150 rpm for 24 h and biofilms were established at the air-liquid interface. The plates were rinsed once with PBS buffer, the biofilms were stained by 150 μL 0.1% CV at room temperature for 15 min and then rinsed twice with PBS buffer. The absorbance was quantified with a plate reader (Synergy 2, BioTek, Santa Clara, CA, USA) at 550 nm using ethanol (95%). DMSO (2.5%) and myxovalargin A (250–2 μg/mL) were used as negative and positive controls.

### 3.5. Biofilm Dispersion Assay

A cell suspension of *Staphylococcus aureus* strain DSM 1104 was adjusted to match the turbidity of a 0.001 McFarland standard and incubated in 96 well tissue microtiter plates for 18 h in CASO plus 4% glucose broth. The supernatant was removed from the wells which were washed with 150 µL PBS buffer. 150 µL of fresh medium (CASO with 4% glucose broth) was added together with serially diluted compounds (250–0.13 µg/mL). The plates were incubated for a further 24 h at 37 °C. Staining of the preformed biofilm and controls was done as described for the biofilm inhibition [12,17]. All experiments were run in duplicates with two repetitions (SD ≤ 10%).

*Candida albicans* DSM 11225 was cultured in 25 mL YPED (Yeast extract Peptone Dextrose) medium in a 250 mL flask at 30 °C in a shaker (100 rpm) for 18 h. The OD_600_ of the suspension was adjusted to 0.05 McFarland standard in RPMI 1640 medium. 150 μL solution was added to 96 well non-tissue microtiter plates (Falcon non-tissue plate ref. no. 351172) at 37 °C at 150 rpm. After 90 min the supernatant was removed and the plates were washed twice with PBS buffer. The test compounds were serially diluted in 150 μL of fresh medium (RPMI 1640) to various concentrations (250–2 μg/mL) and added to the wells. DMSO (2.5%) and farnesol (250–2 μg/mL) served as negative and positive controls. The plates were incubated at 37 °C at 150 rpm for 24 h, the supernatant was discarded, and the biofilms were stained by 150 μL 0.1% CV at room temperature for 25 min after having been washed once with PBS buffer. The plates were washed four times with PBS buffer. The biofilms were dissolved in 150 μL ethanol (95%) and the absorbance was quantified with a plate reader (Synergy 2, BioTek, Santa Clara, CA, USA) at 610 nm. All experiments were run in duplicates with two repetitions (SD ≤ 10%).

### 3.6. Cytotoxicity Assay

Cytotoxic effects on human endocervical adenocarcinoma KB-3-1 (ACC 158) cells and mouse fibroblasts L929 (ACC 2) upon treatment with ancorinoside B (**2**) or epothilone B as a positive control were determined by standard MTT assays [19].

### 3.7. Human Cancer Cell MMP Assay (Gelatin Zymography)

*Cell lines, culture conditions, and stock solutions.* 518A2 human melanoma cells (Department of Radiotherapy and Radiobiology, University Hospital Vienna, Vienna, Austria) were grown in Dulbecco’s Modified Eagle Medium (DMEM; Pan Biotec, Aidenbach, Germany) supplemented with 10% (*v*/*v*) fetal bovine serum (FBS; Biochrom, Berlin, Germany) and 1% (*v*/*v*) Antibiotic-Antimycotic solution (Gibco, Waltham, MA, USA). Cells were incubated at 37 °C, 5% CO_2_, 95% humidified atmosphere, and were serially passaged following trypsinization by using 0.05% trypsin/0.02% EDTA (*w*/*v*; Biochrom, GmbH, Berlin, Germany). Mycoplasma contamination was frequently monitored, and only mycoplasma-free cultures were used. 10 mM stock solutions of test compounds were prepared in DMSO and stored at −23 °C.

*Assessment of MMP-2 and MMP-9 activity by gelatin zymography* [20]. The influence of test compounds on expression respectively secretion of matrix metalloproteases (MMP) of type-2 and -9 in 518A2 melanoma cells was assessed by gelatin zymography. Cells were seeded with 3 mL per well in 6 well plates with a cell density of 0.1 × 10^6^ cells per mL, followed by an incubation period of 24 h at standard cell culture conditions. The medium was aspirated and replaced by 1.5 mL DMEM and 5 µL per well aprotinin solution of a 10 mg/mL stock solution was added. 15 µL per well of 100-fold concentrated test compound dilutions were added, resulting in final concentrations of 500 nM, 1 µM, 25 µM, and 50 µM, as well as the corresponding amount of DMSO as a negative control. Treated cells were incubated for a further 24 h under standard cell culture conditions. Harvesting started with transferring the supernatant of each well into a fresh 2 mL micro reaction tube, followed by rinsing each well with 500 µL of phosphate-buffered saline (PBS). Then 500 µL of cell lysis buffer (0.1 M Tris-Cl, 0.2% Triton X 100 ad ddH_2_O, pH 7.4, immediately before use: addition of 10 µL aprotinin solution (10 mg/mL stock per 3 mL buffer) were added, and cells were detached using a cell scraper. Cell lysates were also transferred to fresh 2 mL micro reaction tubes, vortexed for at least 10 s, and incubated on ice for a further 15 min. Supernatant and cell lysate samples were centrifuged (20 min, 30,000× *g*, 4 °C). The supernatant of each tube was transferred into a fresh tube. The protein concentration of each sample was determined using a standard Bradford assay. Samples were prepared to contain 80 µg protein, non-reducing 5× protein sample buffer (10% sodium dodecyl sulfate (SDS), 0.5 M Tris-Cl, 1:1 glycerol, bromophenol blue) was added, and samples were loaded on a polyacrylamide gel, composed of 4% collecting gel and 10% separating gel containing 1 mg/mL gelatin. Electrophoresis was conducted for 30 min with 80 V and for 2–3 h with 100 V. The gels were washed 10 min each in washing buffer I (50 mM Tris-Cl, 2% Triton X 100, ad ddH_2_O, pH 7.4) and washing buffer II (50 mM Tris-Cl, ad ddH_2_O, pH 7.4), followed for incubation in substrate buffer (50 mM Tris-Cl, 1% Triton X 100, 5 mM CaCl_2_, ad ddH_2_O, pH 7.4) for 16 h at 37 °C. The gels were stained for 30 min in Coomassie gel staining solution (500 mg Coomassie brilliant blue, 454 mL MeOH, 454 mL ddH_2_O, 100 mL acetic acid glacial) and destained for 1–2 h in destaining solution (500 mL MeOH, 100 mL acetic acid glacial, 1.4 L ddH_2_O) until light bands became visible. Gels were documented and densitometrically evaluated using AzureSpot analysis software. All experiments were run in duplicates with two repetitions.

## 4. Conclusions

The first synthesis of the marine sponge metabolite ancorinoside B (**2**) proceeded with an overall yield of 4% over 16 steps in the longest linear reaction sequence. Its modular character should also allow for the synthesis of other, including non-natural, oligoglycosidic 3-acyltetramic acids with variance in the sugars, the tether length, and functionalization, as well as in the amino acid constituting the tetramate moiety. This is of medicinal interest, as Fusetani et al. had already observed that slight structural changes in 3-acyltetramic acids can lead to significantly different inhibitory effects on the activity of cellular matrix metalloproteinases which are essential enzymes in the tumoral metastasis cascade. The remarkably strong antibiofilm effects of ancorinoside B (**2**) at concentrations not toxic to the constituent microorganisms, and its reducing effect on the secretion and expression of matrix metalloproteinases 2 and 9 make it a promising candidate for application as a pleiotropic agent in a medicinal context, e.g., for the control of the general hospital, catheter, or joint protheses infections.

## Figures and Tables

**Table 1:** Antimicrobial activity of synthetic ancorinoside B (**2**).

Tested Organisms	Strain No.	MIC [µg/mL]	
		Ancorinoside B (2)	Reference ^1^
Bacteria			
*Bacillus subtilis*	DSM 10	16.6	4.2 ^a^
*Staphylococcus aureus*	DSM 346	66.6	0.4 ^a^
*Mycobacterium smegmatis*	ATCC 700084	inactive	1.7 ^b^
*Acinetobacter baumannii*	DSM 30008	inactive	0.5 ^c^
*Chromobacterium violaceum*	DSM 30191	inactive	0.8 ^a^
*Escherichia coli*	DSM 1116	inactive	3.3 ^a^
*Pseudomonas aeruginosa*	PA 14	inactive	0.4 ^d^
Fungi			
*Mucor hiemalis*	DSM 2656	66.6	8.3 ^e^
*Pichia anomala*	DSM 6766	inactive	8.3 ^e^
*Rhodotorula glutinis*	DSM 10134	66.6	2.1 ^e^
*Candida albicans*	DSM 1665	inactive	8.3 ^e^
*Schizosaccharomyces pombe*	DSM 70572	inactive	4.2 ^e^

^1^ References: ^a^ oxytetracycline, ^b^ kanamycin, ^c^ ciprobay, ^d^ gentamicin, ^e^ nystatin.

**Table 2:** Inhibition of biofilm formation of *S. aureus* and dispersion of preformed biofilms of *S. aureus* and *C. albicans* by ancorinoside B (**2**) at various concentrations.

Tested Organisms	Strain No.	Biofilm Inhibition [% ± SD]	Biofilm Dispersion [% ± SD]
		Ancorinoside B (2)	
*Staphylococcus aureus*	DSM 1104	87 ± 3 (0.5 µg/mL) ^a^	79 ± 4 (62.5 µg/mL) ^b^ 67 ± 6 (2 µg/mL) ^b^
		72 ± 4 (0.3 µg/mL) ^a^	
		22 ± 4 (0.13 µg/mL) ^a^	
*Candida albicans*	DSM 11225	–	55 ± 10 (250 µg/mL) ^c^

References [%]: ^a^ microporenic acid A (MAA): 86 (31.3 µg/mL), 77 (7.8 µg/mL), 45 (3.9 µg/mL); ^b^ MAA: 62 (250 µg/mL), 40 (62.5 µg/mL), 48 (31.3 µg/mL); ^c^ farnesol: 81 (250 µg/mL), 70 (31.3 µg/mL), 38 (15.6 µg/mL); SD: standard deviation; –: not tested.

## Data Availability

The datasets used and/or analyzed during the current study are available from the corresponding author upon reasonable request.

## Supplementary Materials

The following are available online at https://www.mdpi.com/article/10.3390/md19100583/s1, Figure S1: Images of gelatin gels, Table S1: NMR-Comparison of isolated and synthetic ancorinosid B tris(diethylammonium) salt [NEt_2_H_2_]_3_^+^ [**2**-3H]^3^.

## References

[1] Royles B.J.L. (1995). Naturally occurring tetramic acids: Structure, isolation, and synthesis. Chem. Rev..

[2] Ghisalberti E.L., Rahman A.-u. (2003). Studies in Natural Products Chemistry.

[3] Gossauer A., Herz W., Falk H., Kirby G.W. (2003). Monopyrrolic natural compounds including tetramic acid derivatives. Progress in the Chemistry of Organic Natural Products.

[4] Schobert R., Schlenk A. (2008). Tetramic and tetronic acids: An update on new derivatives and biological aspects. Bioorg. Med. Chem..

[5] Sakuda S., Matsumori N., Furihata K., Nagasawa H. (2007). Assignment of the absolute configuration of blasticidin A and revision of that of aflastatin A. Tetrahedron Lett..

[6] Ohta S., Ohta E., Ikegami S. (1997). Ancorinoside A: A novel tetramic acid glycoside from the marine sponge *Ancorina sp.* which specifically inhibits the blastulation of starfish embryos. J. Org. Chem..

[7] Fujita M., Nakao Y., Matsunaga S., Seiki M., Itoh Y., van Soest R.W.M., Fusetani N. (2001). Ancorinosides B-D, inhibitors of membrane type 1 matrix metalloproteinase (MT1-MMP), from the marine sponge *Penares sollasi* Thiele. Tetrahedron.

[8] Petermichl M., Schobert R. (2017). Total synthesis of the diglycosidic tetramic acid ancorinoside A. Chem. Eur. J..

[9] Petermichl M., Steinert C., Schobert R. (2019). A synthetic route to the MT1-MMP inhibitor Ancorinoside D. Synthesis.

[10] Ley S.V., Smith S.C., Woodward P.R. (1992). Further reactions of t-butyl 3-oxobutanthioate and t-butyl 4-diethyl-phosphono-3-oxobutanthioate: Carbonyl coupling reactions, amination, use in the preparation of 3-acyltetramic acids and application to the total synthesis of fuligorubin A. Tetrahedron.

[11] Zhang Z., Magnusson G. (1996). Synthesis of double-chain bis-sulfone neoglycolipids of the 2-, 3-, and 6-deoxyglobotrioses. J. Org. Chem..

[12] Chepkirui C., Yuyama K., Wanga L., Decock C., Matasyoh J., Abraham W.R., Stadler M. (2018). Microporenic acids A-G, biofilm inhibitors and antimicrobial agents from the basidiomycete *Microporus* sp. J. Nat. Prod..

[13] Ley S.V., Woodward P.R. (1987). Preparation of t-butyl 4-diethylphosphono-3-oxobutanethioate and use in the synthesis of (*E*)-4-alkenyl-3-oxoesters and macrolides. Tetrahedron Lett..

[14] Xu P., Yang J.Y., Kováč P. (2012). Oberservations on the preparation of β-lactose octaacetate. J. Carbohydr. Chem..

[15] Becker K., Wessel A.C., Luangsaard J.J., Stadler M. (2020). Viridistratins A−C, antimicrobial and cytotoxic benzo [j] fluoranthenes from stromata of *Annulohypoxylon viridistratum* (Hypoxylaceae, Ascomycota). Biomolecules.

[16] Sandargo B., Michehl M., Praditya D., Steinmann E., Stadler M., Surup F. (2019). Antiviral meroterpenoid rhodatin and sesquiterpenoids rhodocoranes A–E from the Wrinkled Peach Mushroom, *Rhodotus palmatus*. Org. Lett..

[17] Schriefer M.G., Schrey H., Zeng H., Stadler M., Schobert R. (2021). Synthesis of the fungal macrolide berkeleylactone A and its inhibition of microbial biofilm formation. Org. Biomol. Chem..

[18] O’Toole G.A. (2011). Microtiter dish biofilm formation assay. J. Vis. Exp..

[19] Mosmann T. (1983). Rapid colorimetric assay for cellular growth and survival: Application to proliferation and cytotoxicity assays. J. Immunol. Methods.

[20] Toth M., Sohail A., Fridman R., Dwek M., Brooks S., Schumacher U. (2012). Assessment of gelatinases (MMP-2 and MMP-9) by gelatin zymography. Metastasis Research Protocols.

