# Supplementary material for: Synthesis and Bioactivity of Ancorinoside B, a Marine Diglycosyl Tetramic Acid
Source: Mar Drugs. 2021 Oct 19;19(10):583. doi: 10.3390/md19100583 (PMC8541288; doi:10.3390/md19100583)
Supplement: Supplementary file 1 [file marinedrugs-19-00583-s001.zip › marinedrugs-1431391-supplementary.pdf]

Supporting information

Synthesis and Bioactivity of Ancorinoside B, a Marine Diglycosyl Tetramic Acid

Kevin J. Soliga, Sofia I. Bär, Natalie Oberhuber, Haoxuan Zeng, Hedda Schrey and Rainer Schobert*

Rainer.Schobert@uni-bayreuth.de

Table of content **Page**

Table S1: NMR-Comparison of isolated and synthetic ancorinoside B tris(diethylammonium) salt $[NEt_2H_2]_3^+$ [**2**-3H]$^{3-}$ S2

NMR spectra and HPLC chromatograms S3-S28

HRMS spectra and chromatograms of key compounds **2**, **8**, **6**, and **5** S29

Figure S1: images of gelatin gels S33

NMR-Comparison of isolated and synthetic Ancorinoside B tris(diethylammonium) salt

Table S1. Comparison of ^{13}C and ^{1}H NMR shifts of isolated and synthetic Ancorinoside B tris(diethylammonium) salt

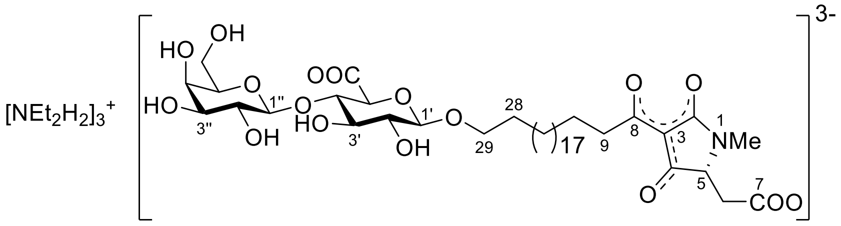

	natural $[NEt_2H_2]^+_3[\mathbf{2}\text{-}3H]^{3-}$ in CD_3OD[7]		synthetic $[NEt_2H_2]^+_3[\mathbf{2}\text{-}3H]^{3-}$ in CD_3OD	
Pos.	δ_C	δ_H (mult., *J* [Hz])	δ_C	δ_H (mult., *J* [Hz])
2	175.7		176.1	
3	102.3		102.4	
4	195.7		196.7	
5	63.6	3.88 (dd, 8.1, 4.2)	64.7	3.90-3.95 (m)
6	39.1	2.78 (dd, 16.2, 4.2),<br>2.37 (dd, 16.2, 8.1)	41.7	2.76-2.80 (m),<br>2.13 (dd, 15.3, 9.2)
7	177.1		179.9	
8	197.0		197.8	
9	40.5	2.78 (m),<br>2.73 (m)	41.1	2.80-2.84 (m),<br>2.70-2.76 (m)
10	27.3	1.55 (quin, 7.3)	27.6	1.51-1.58 (m)
11	30.8	1.32 (m)	31.0	1.24-1.43 (m)
12-26	30.8	1.27 (m)	31.0	1.24-1.43 (m)
27	27.2	1.36 (m)	27.3	1.24-1.43 (m)
28	30.8	1.60 (quin, 7.3)	30.9	1.58-1.66 (m)
29	71.0	3.88 (m),<br>3.51 (m)	71.2	3.82-3.89 (m),<br>3.52-3.54 (m)
*N*Me	27.4	2.88 (s)	27.8	2.91 (s)
1'	104.2	4.29 (d, 7.7)	104.3	4.30 (d, 7.9)
2'	74.6	3.28 (t, 8.1)	74.7	3.27-3.29 (m)
3'	76.7	3.50 (t, 8.9)	76.8	3.49-3.52 (m)
4'	83.5	3.59 (t, 9.2)	83.6	3.59-3.62 (m)
5'	77.2	3.72 (d, 9.6)	77.3	3.69-3.73 (m)
6'	176.2		176.5	
1''	105.5	4.35 (d, 7.7)	105.6	4.35 (d, 7.3)
2''	72.7	3.53 (dd, 10.0, 7.7)	72.8	3.54-3.56 (m)
3''	75.0	3.47 (dd, 10.0, 3.8)	75.1	3.48 (dd, 9.9, 3.5)
4''	70.3	3.78 (d, 3.8)	70.4	3.77-3.79 (m)
5''	77.2	3.56 (m)	77.4	3.56-3.59 (m)
6''	62.5	3.76 (dd, 11.5, 7.3),<br>3.68 (d, 11.5, 4.6)	62.6	3.73-3.77 (m)<br>3.66-3.69 (m)
$3HN(CH_2CH_3)_2$ (C*H*$_2$)	-[a]	-[a]	43.6	3.02 (q, 7.0)
$3HN(CH_2CH_3)_2$ (C*H*$_3$)	-[a]	-[a]	11.9	1.24-1.43 (m)

[a] The literature does not state the shifts of the diethylammonium groups

[7] Fujita, M.; Nakao, Y.; Matsunaga, S.; Seiki, M.; Itoh, Y.; van Soest, R. W. M.; Fusetani, N. Ancorinosides B-D, inhibitors of membrane type 1 matrix metalloproteinase (MT1-MMP), from the marine sponge *Penares sollasi* Thiele. *Tetrahedron* **2001**, *57*, 1229-1234.

NMR spectra

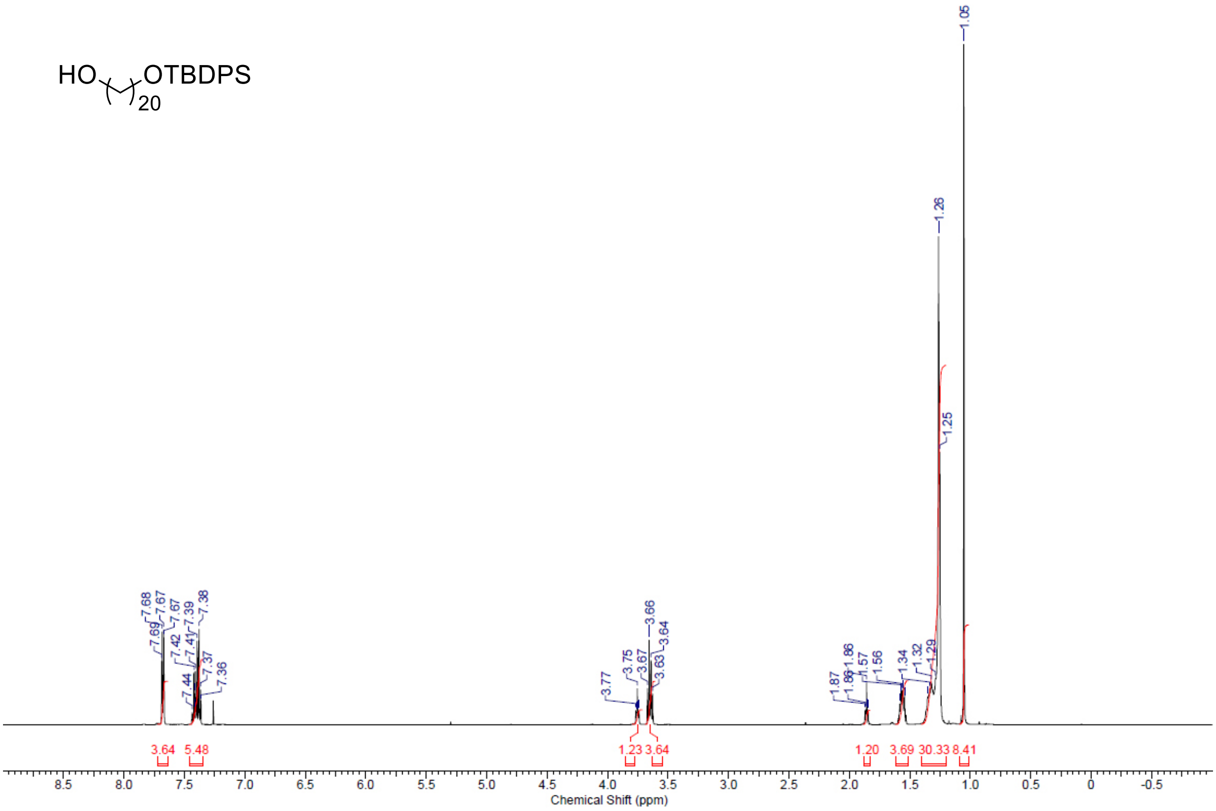

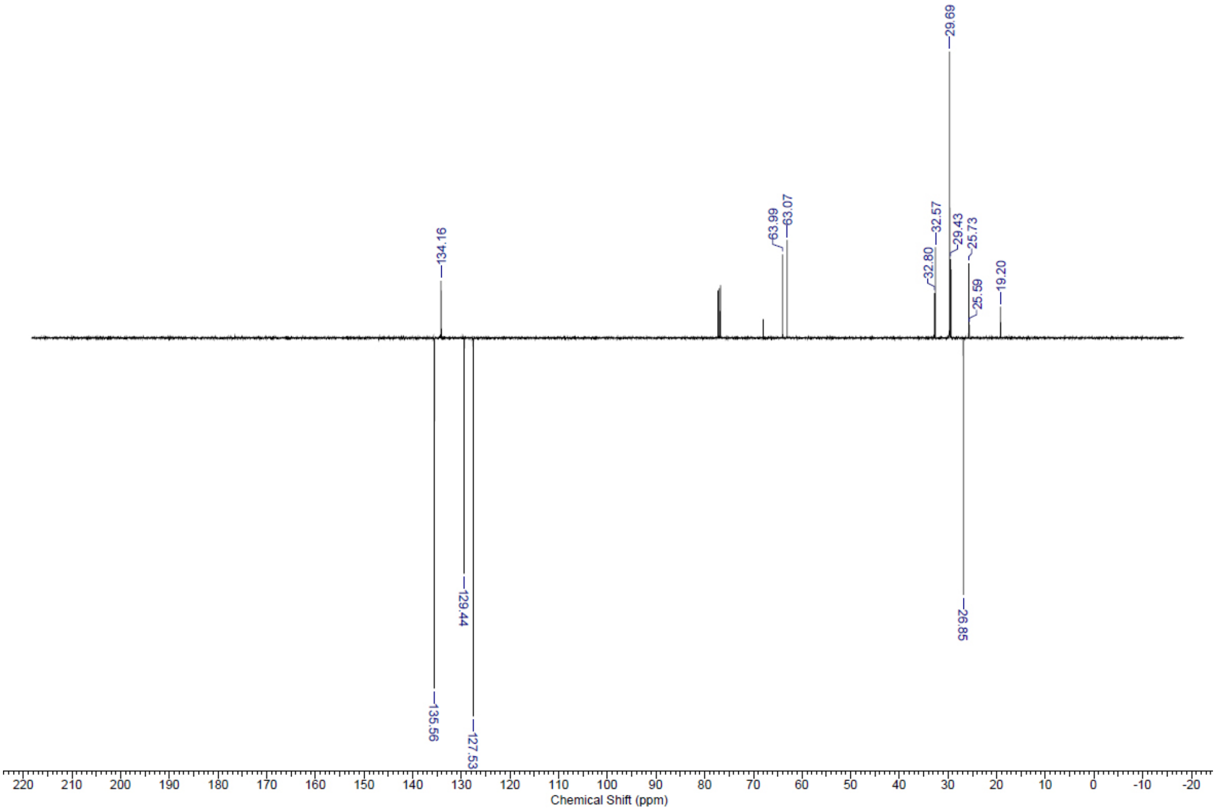

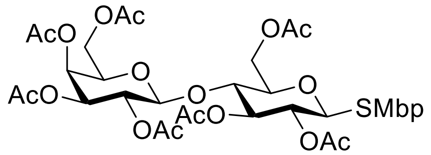
AcO
OAc
O
AcO
O
AcO
OAc
O
SMbp
OAc
OAc

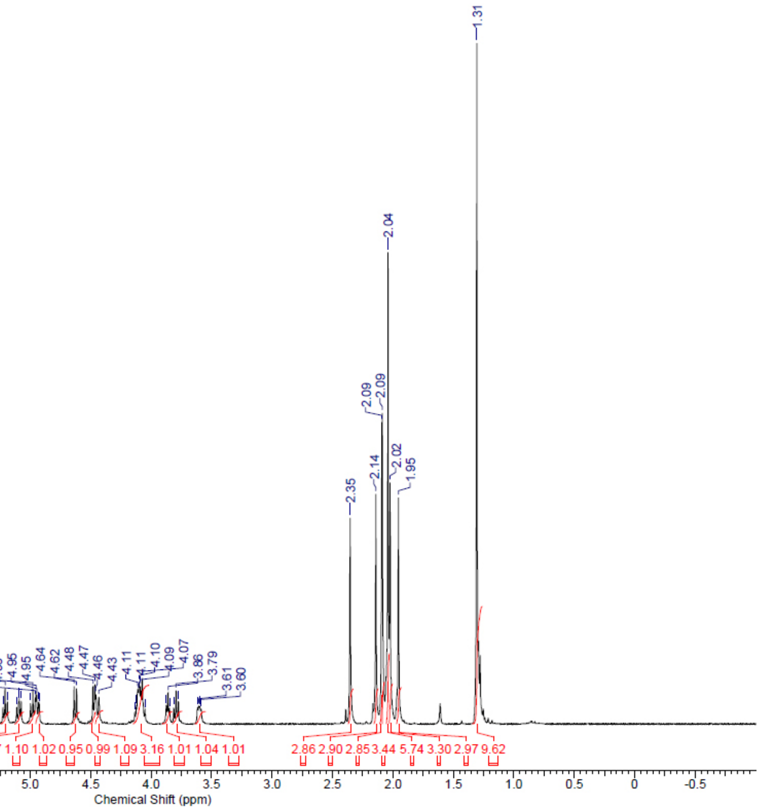
Chemical Shift (ppm)

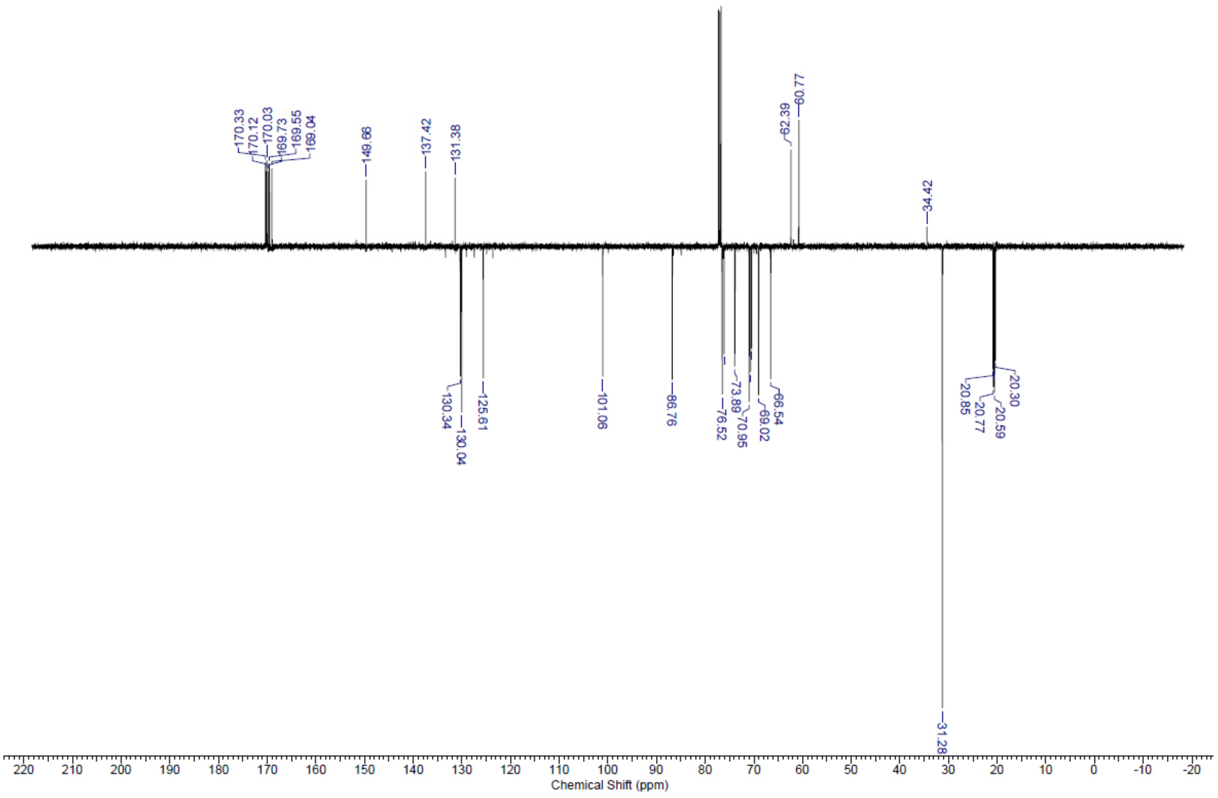
Chemical Shift (ppm)

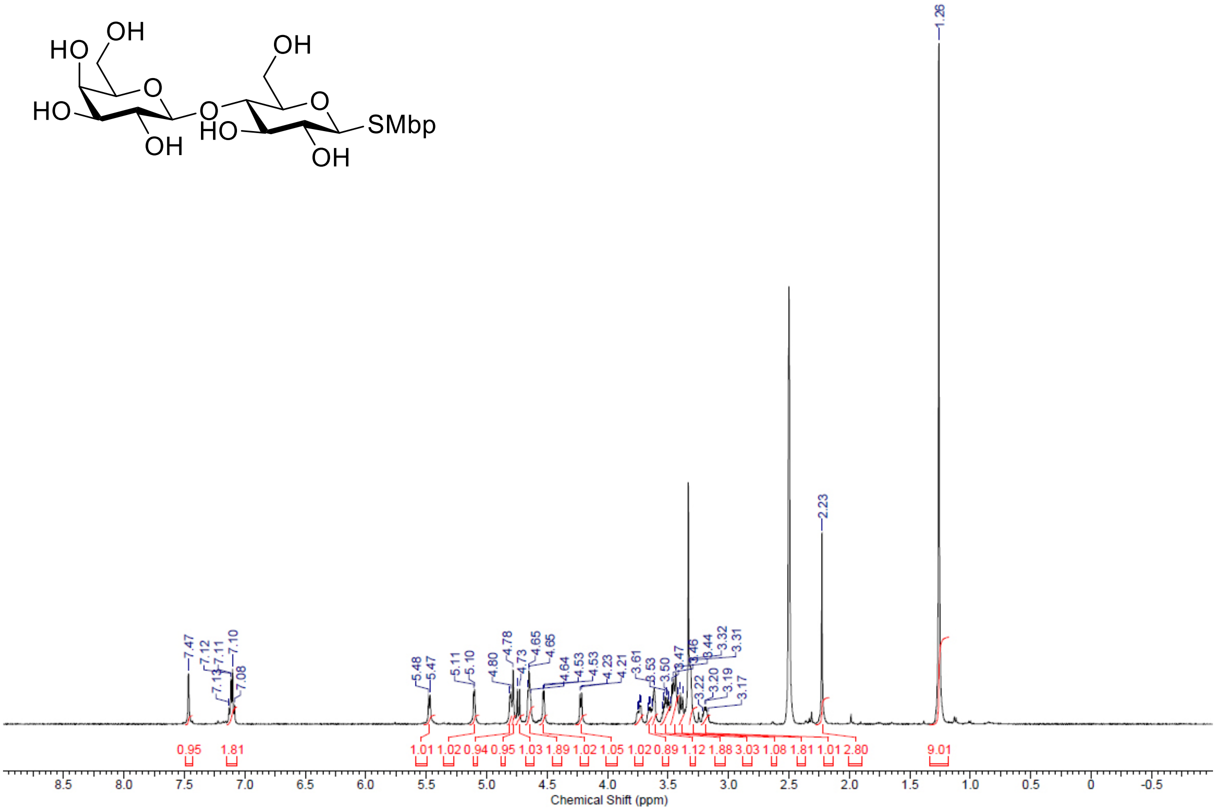
HO
OH
O
HO
OH
O
OH
O
HO
OH
SMbp
7.47
7.12
7.11
7.10
7.13
7.08
5.48
5.47
5.11
5.10
4.80
4.78
4.73
4.65
4.65
4.64
4.53
4.53
4.23
4.21
3.61
3.53
3.50
3.47
3.46
3.44
3.32
3.31
3.22
3.20
3.19
3.17
2.23
1.26
0.95
1.81
1.01
1.02
0.94
0.95
1.03
1.89
1.02
1.05
1.02
0.89
1.12
1.88
3.03
1.08
1.81
1.01
2.80
9.01
Chemical Shift (ppm)

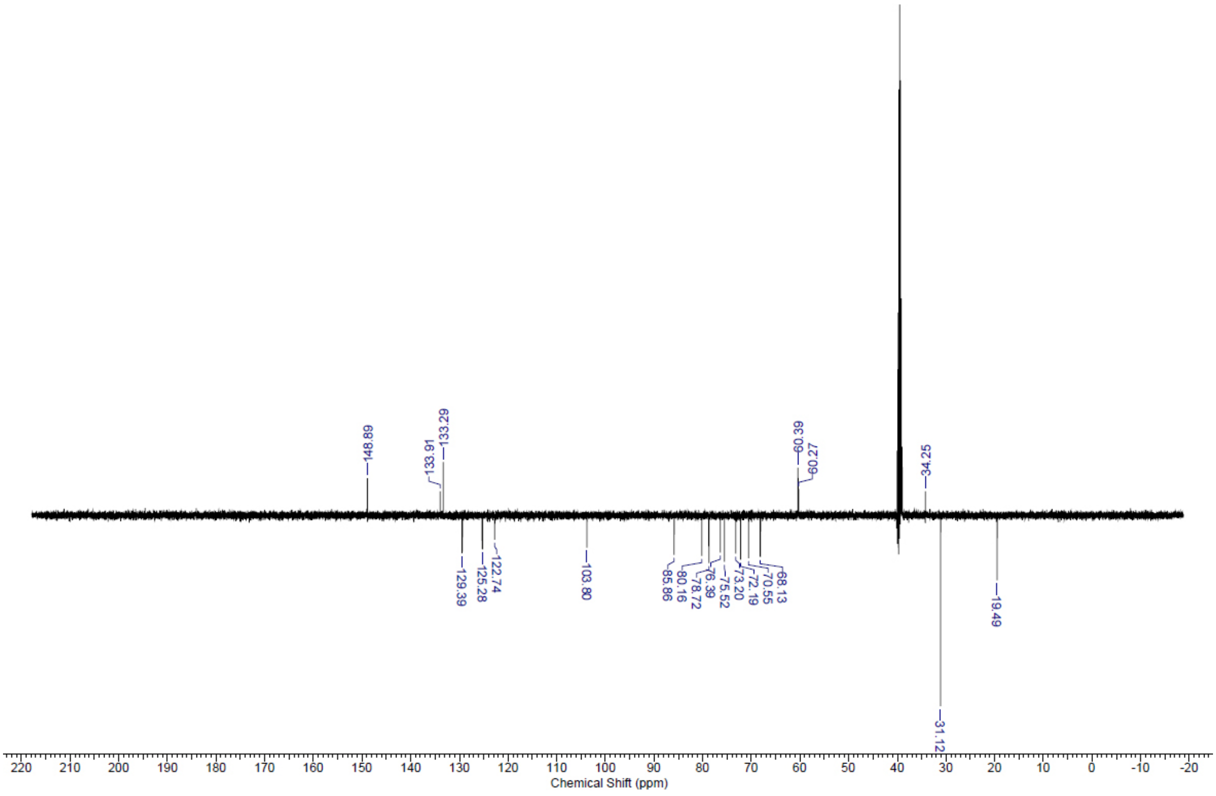
148.89
133.91
133.29
129.39
125.28
122.74
103.80
85.86
80.16
78.72
76.39
75.52
73.20
72.19
70.55
68.13
60.39
60.27
34.25
31.12
19.49
Chemical Shift (ppm)

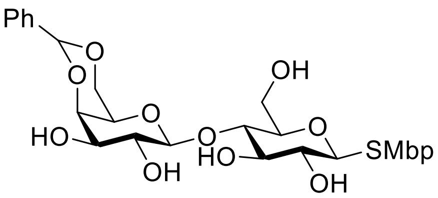
Ph
O
O
O
HO
OH
O
OH
O
HO
OH
SMbp

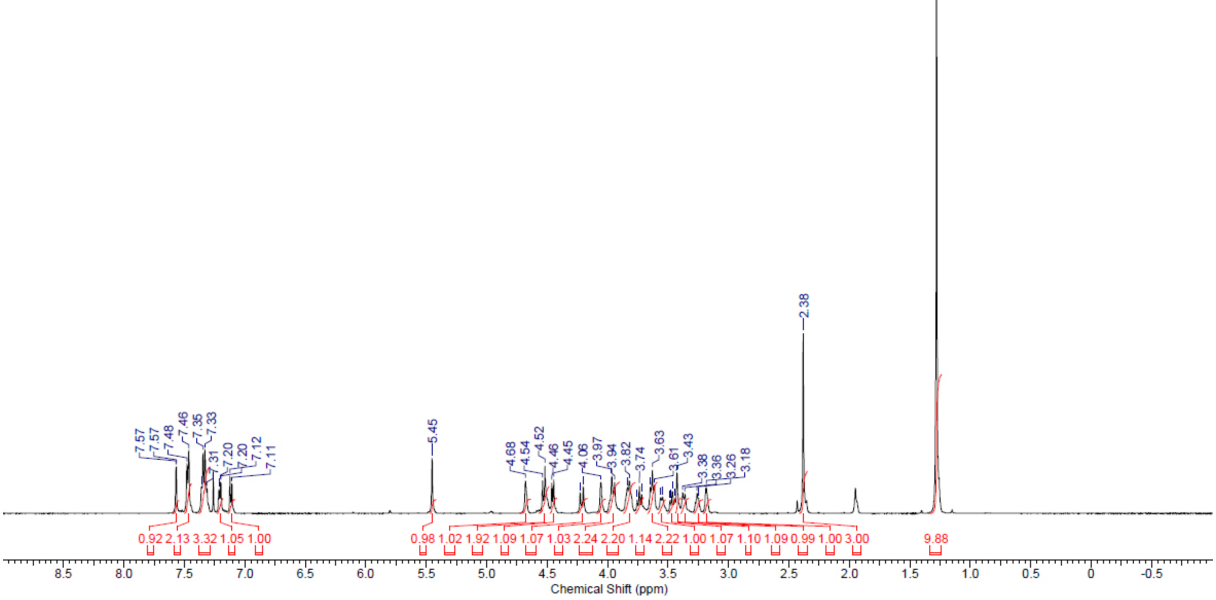
1.28
2.38
5.45
7.57 7.57 7.48 7.46 7.35 7.33 7.31 7.20 7.20 7.12 7.11
4.68 4.54 4.52 4.46 4.45 4.06 3.97 3.94 3.82 3.74 3.63 3.61 3.43 3.38 3.36 3.26 3.18
0.92 2.13 3.32 1.05 1.00 0.98 1.02 1.92 1.09 1.07 1.03 2.24 2.20 1.14 2.22 1.00 1.07 1.10 1.09 0.99 1.00 3.00 9.88
Chemical Shift (ppm)

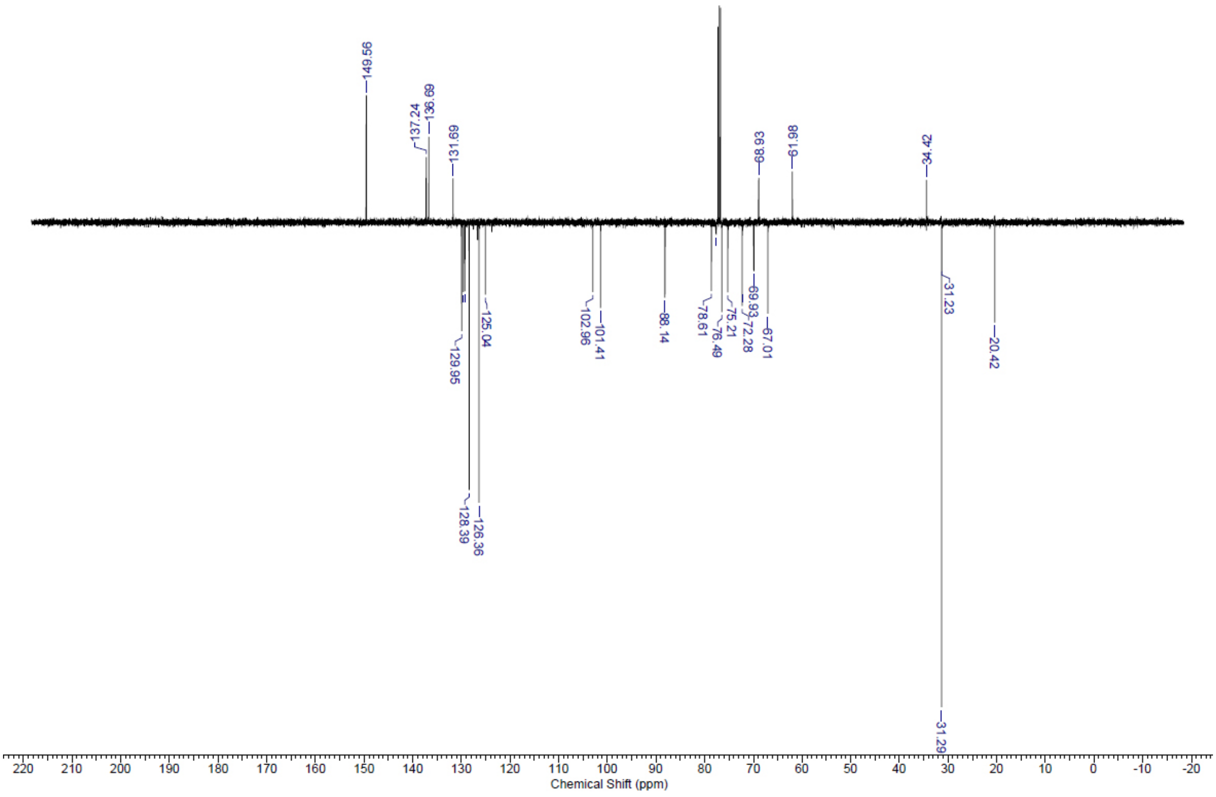
149.56
137.24
136.69
131.69
68.93
61.98
34.42
129.95
125.04
102.96
101.41
88.14
78.61
76.49
75.21
72.28
69.93
67.01
31.23
20.42
128.39
126.36
31.29
Chemical Shift (ppm)

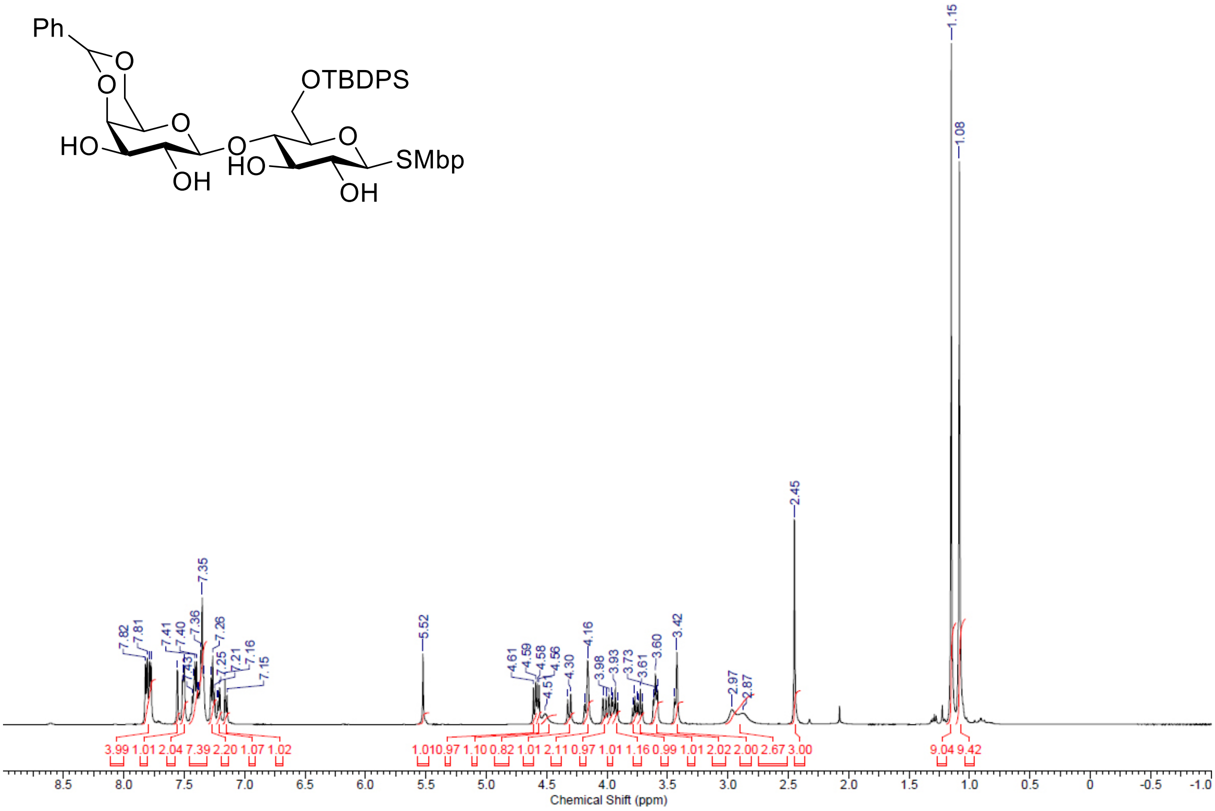
Ph
O
O
O
OTBDPS
HO
O
O
HO
SMbp
OH
OH
Chemical Shift (ppm)

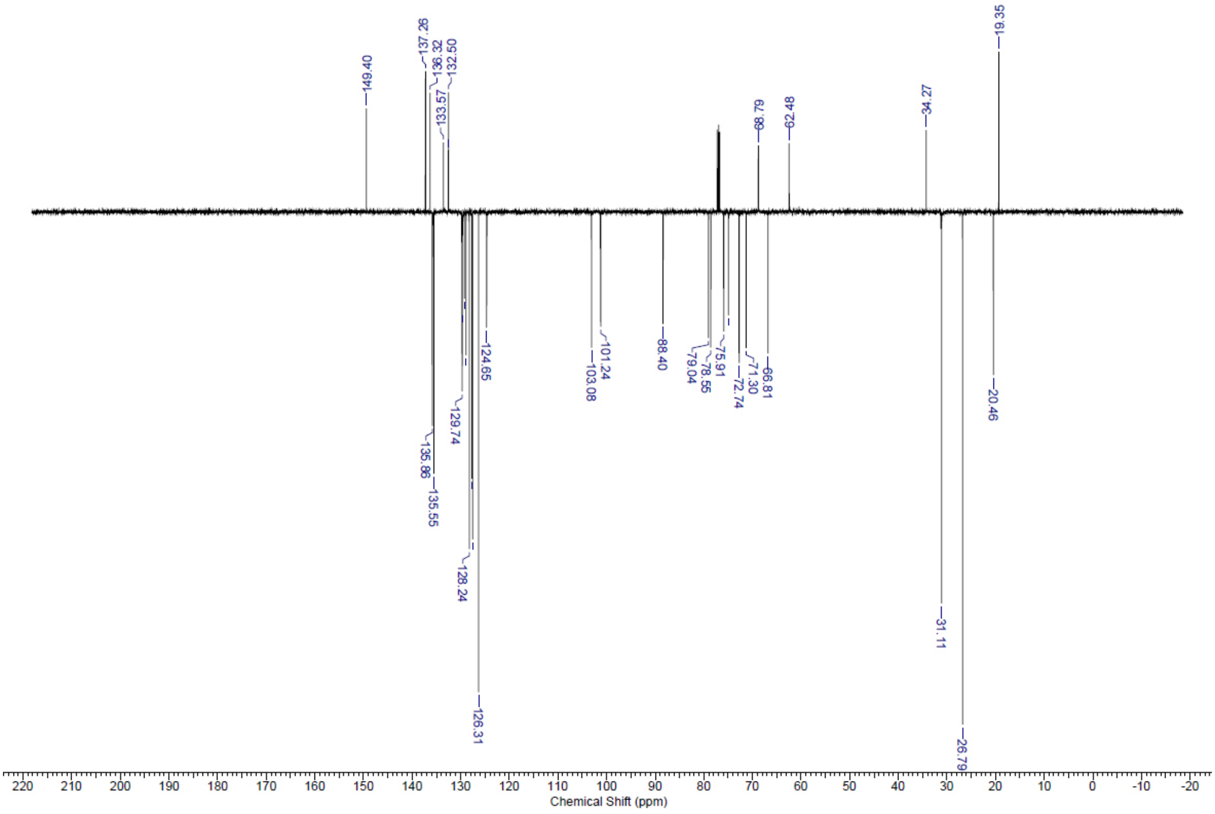
Chemical Shift (ppm)

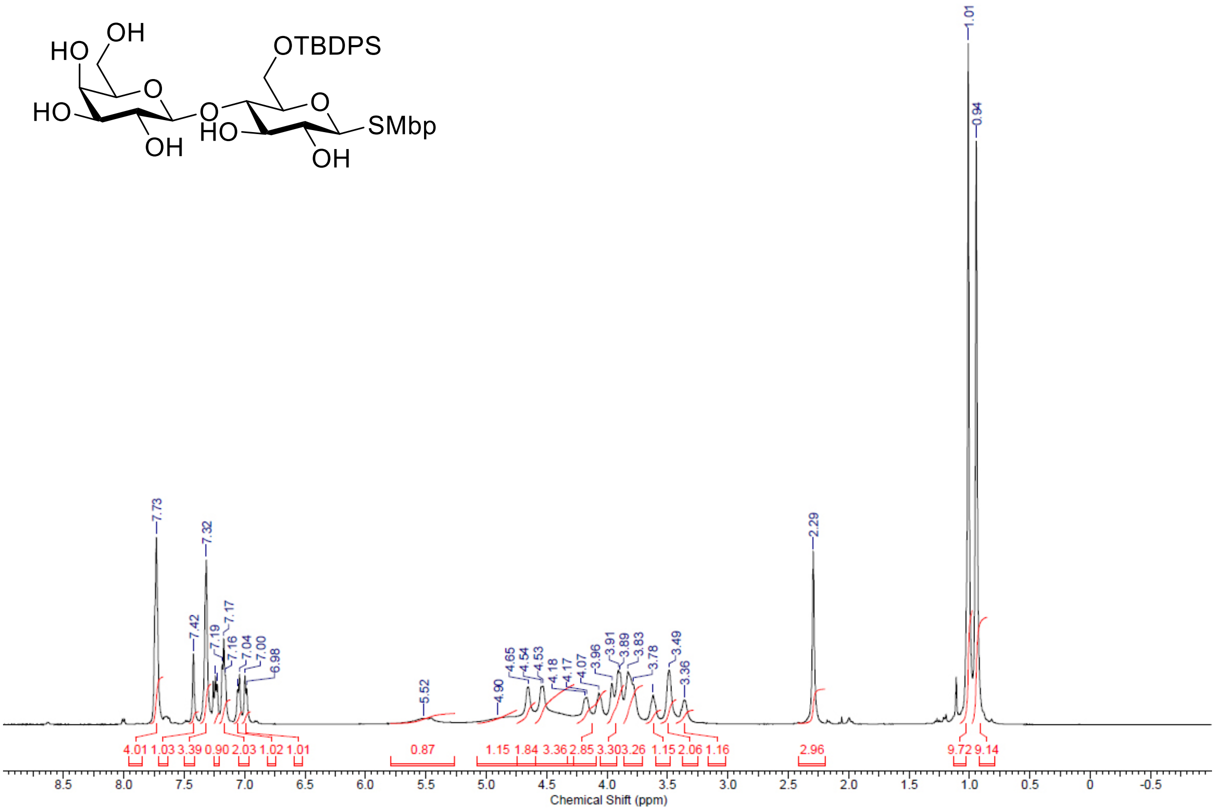
OH
HO
OTBDPS
O
HO
O
O
HO
SMbp
OH
OH
7.73
7.42
7.32
7.19
7.17
7.16
7.04
7.00
6.98
5.52
4.90
4.65
4.54
4.53
4.18
4.17
4.07
3.96
3.91
3.89
3.83
3.78
3.49
3.36
2.29
1.01
0.94
4.01 1.03 3.39 0.90 2.03 1.02 1.01
0.87
1.15 1.84 3.36 2.85 3.30 3.26 1.15 2.06 1.16
2.96
9.72 9.14
Chemical Shift (ppm)

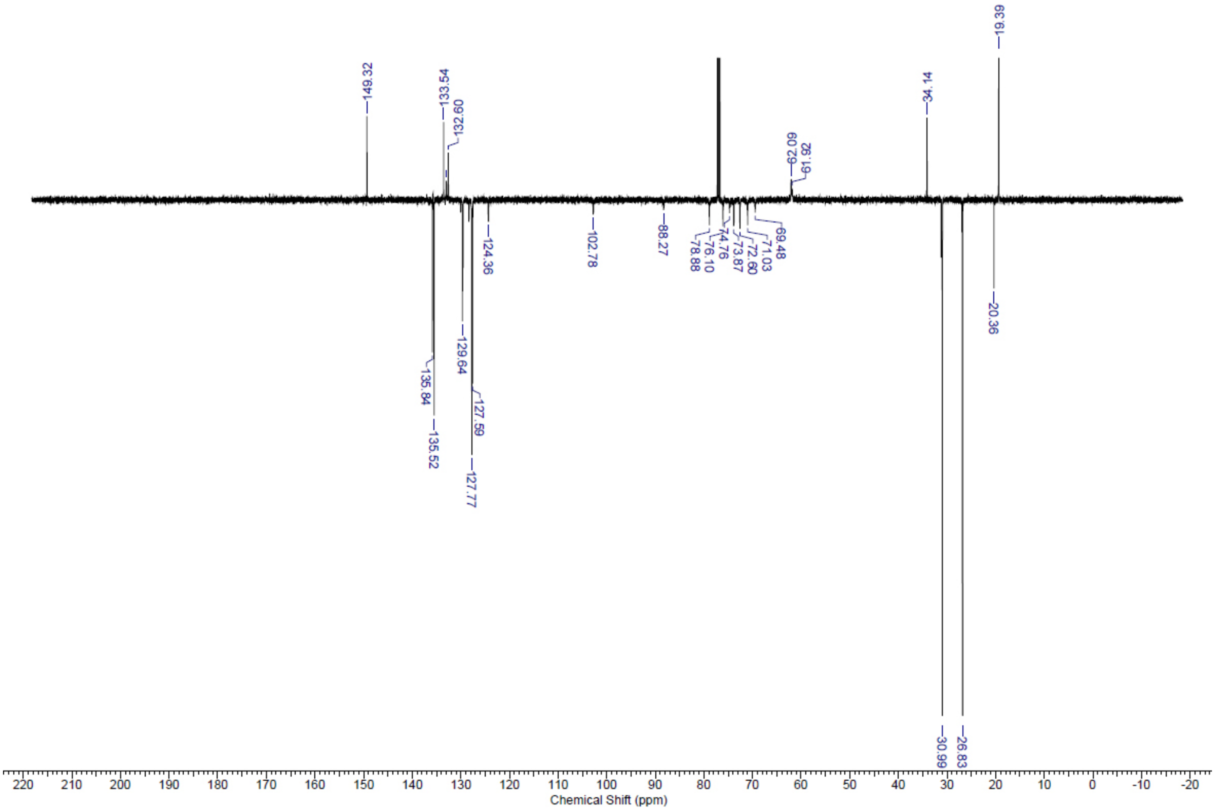
149.32
133.54
132.60
124.36
102.78
88.27
78.88
76.10
74.76
73.87
72.60
71.03
69.48
62.09
61.92
34.14
19.39
135.84
135.52
129.64
127.59
127.77
20.36
30.99
26.83
Chemical Shift (ppm)

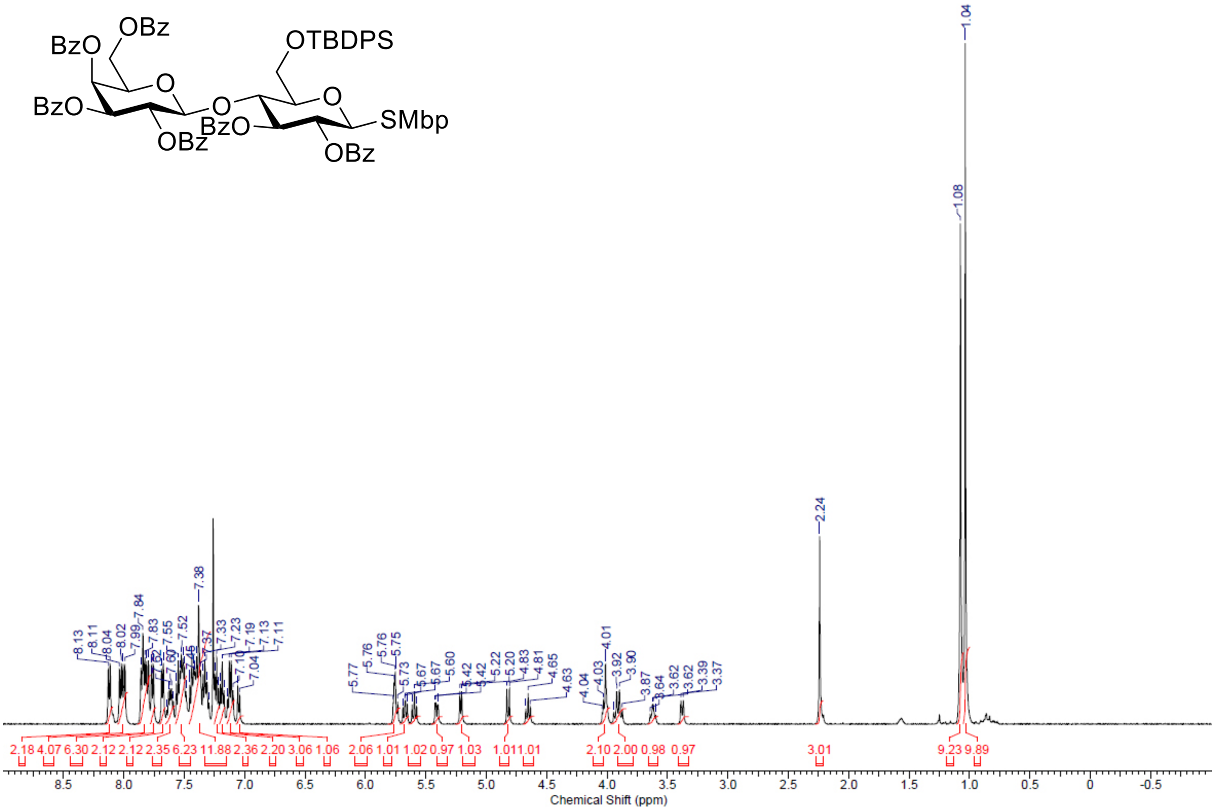
OBz
BzO
O
BzO
OBz
O
BzO
OTBDPS
O
SMbp
OBz
1.04
1.08
2.24
Chemical Shift (ppm)

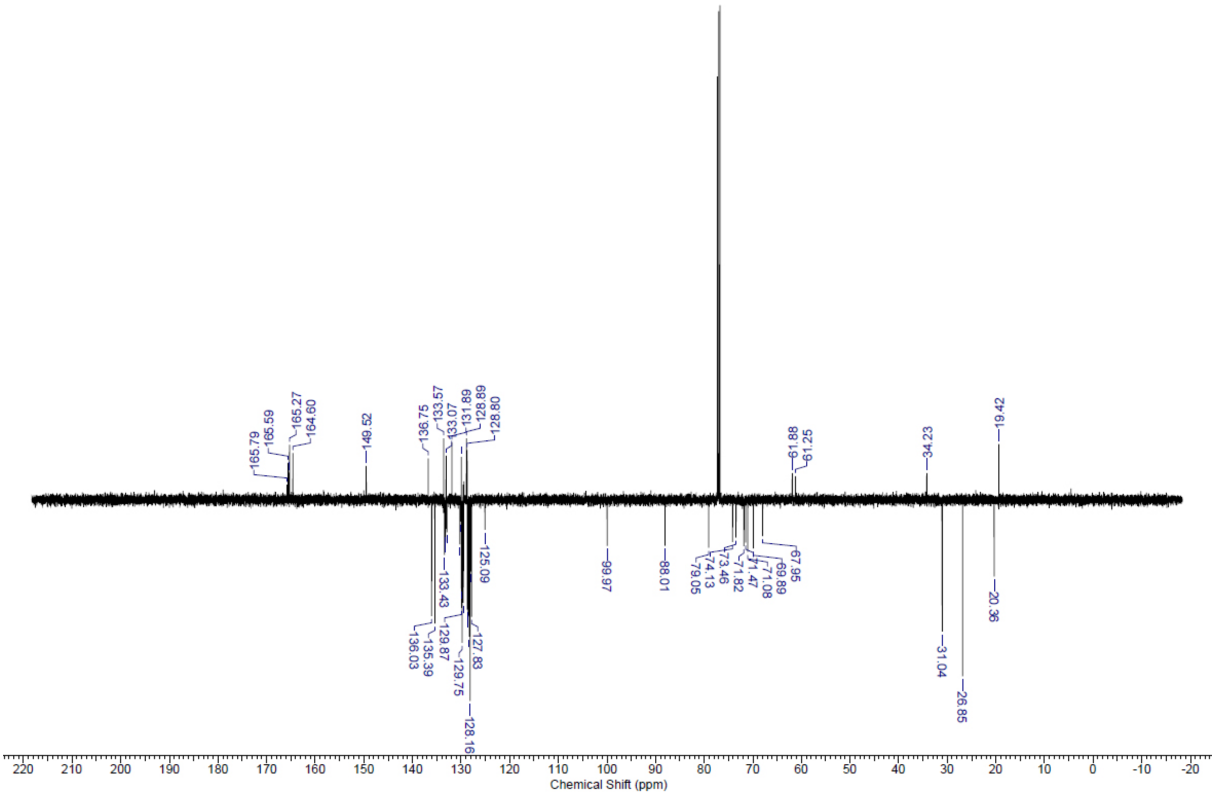
165.79
165.59
165.27
164.60
149.52
136.75
133.57
133.07
131.89
128.89
128.80
61.88
61.25
34.23
19.42
125.09
99.97
88.01
79.05
74.13
73.46
71.82
71.47
71.08
69.89
67.95
133.43
136.03
135.39
129.87
129.75
127.83
128.16
31.04
26.85
20.36
Chemical Shift (ppm)

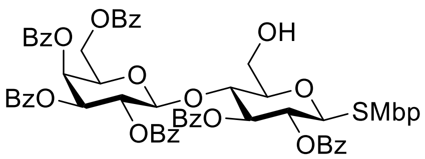
BzO
OBz
O
OH
BzO
O
O
BzO
SMbp
OBz
OBz

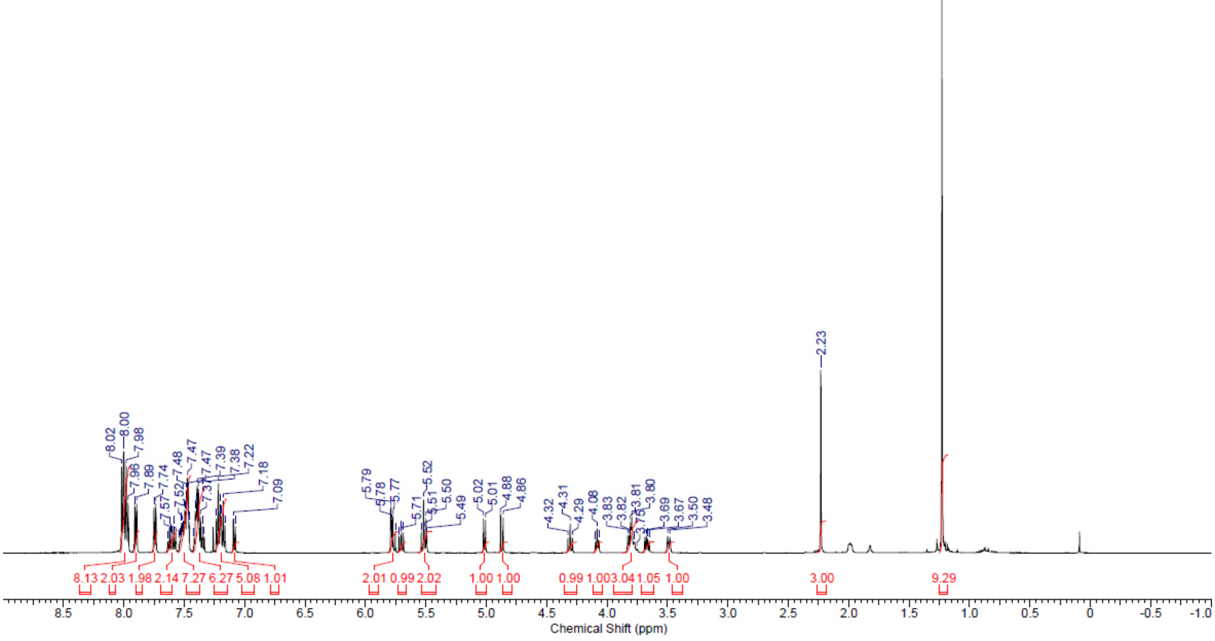
Chemical Shift (ppm)

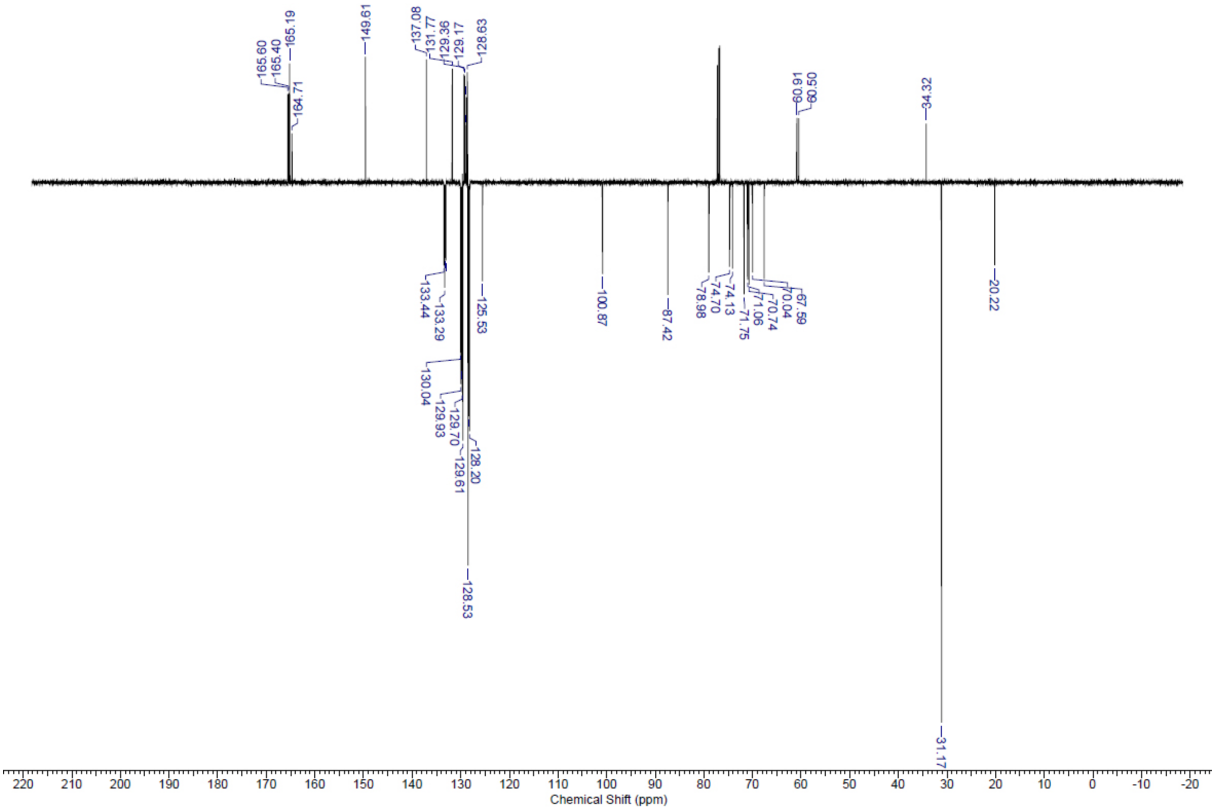
Chemical Shift (ppm)

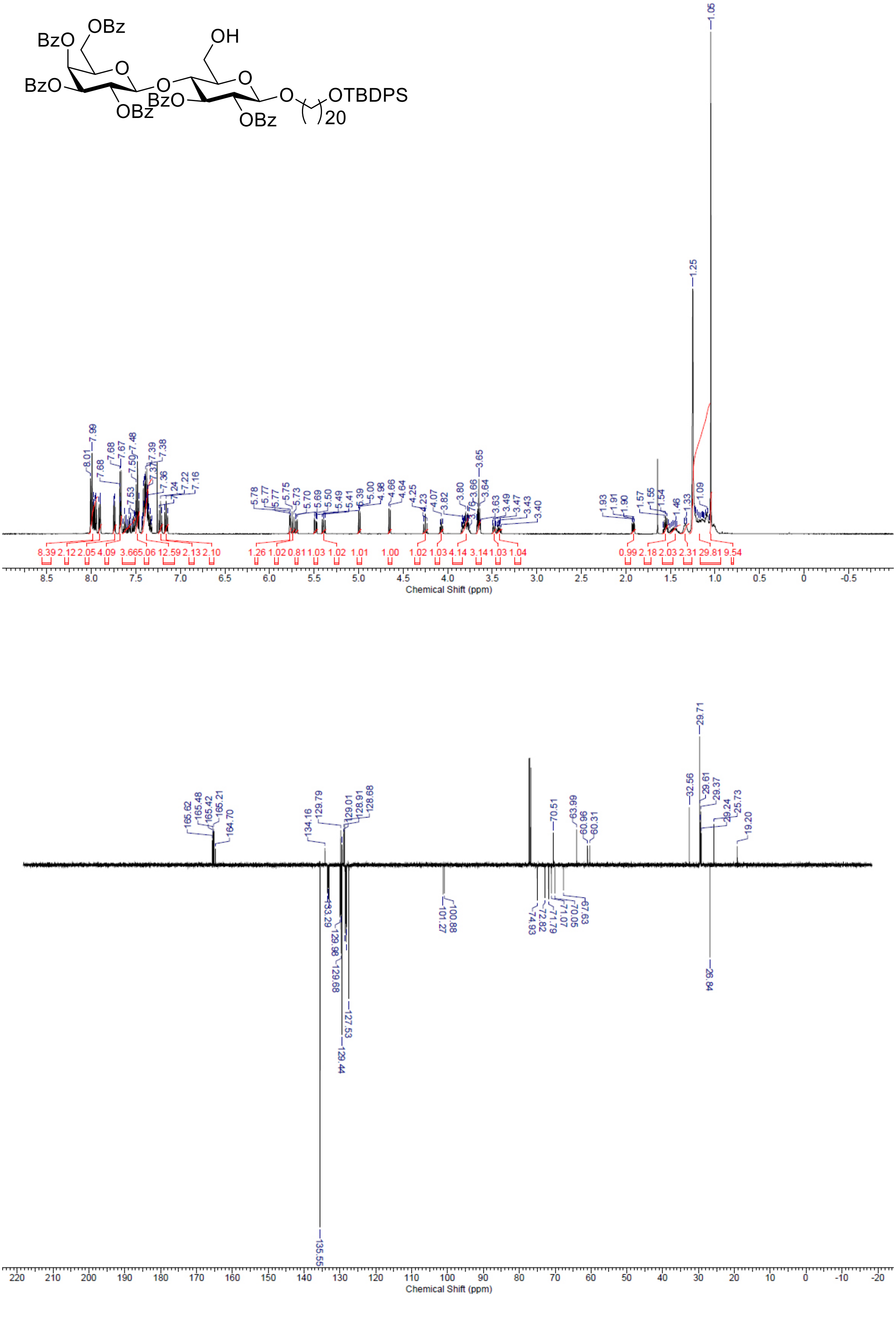

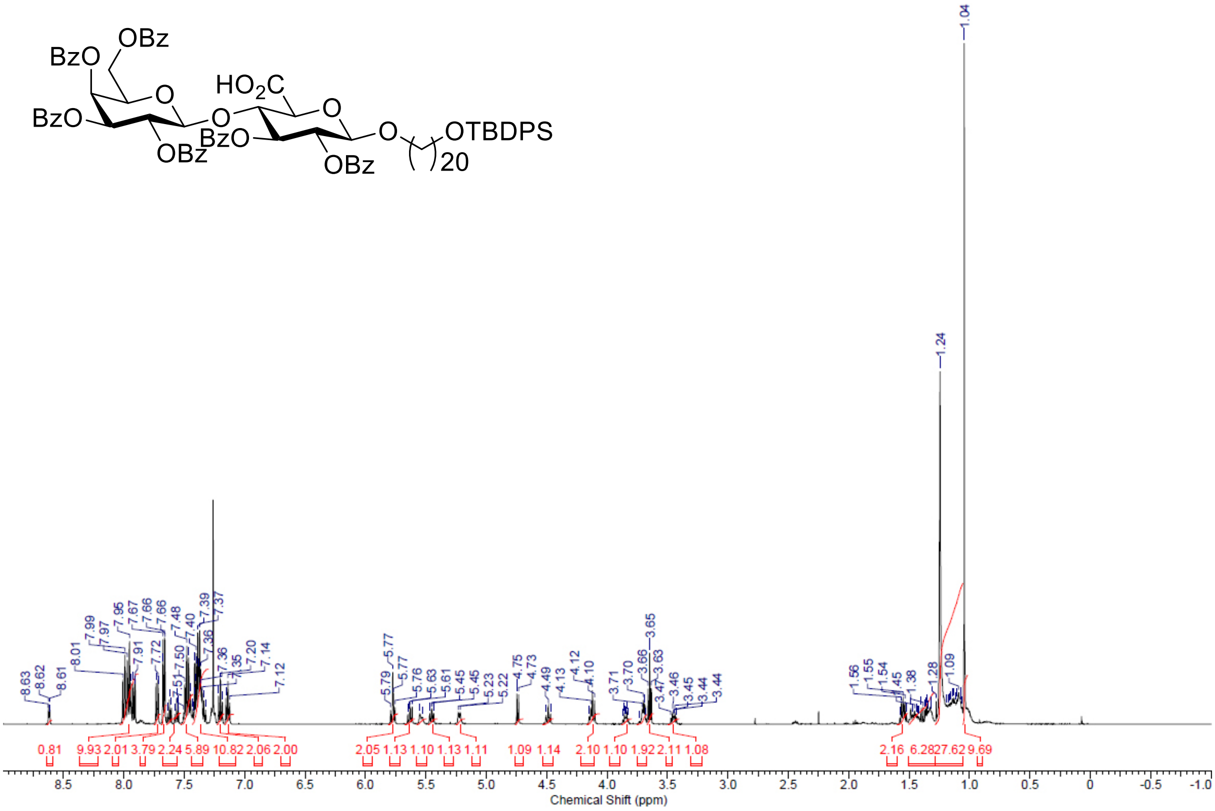
BzO
OBz
BzO
O
OBz
O
HO2C
BzO
O
OBz
O
OTBDPS
20
Chemical Shift (ppm)

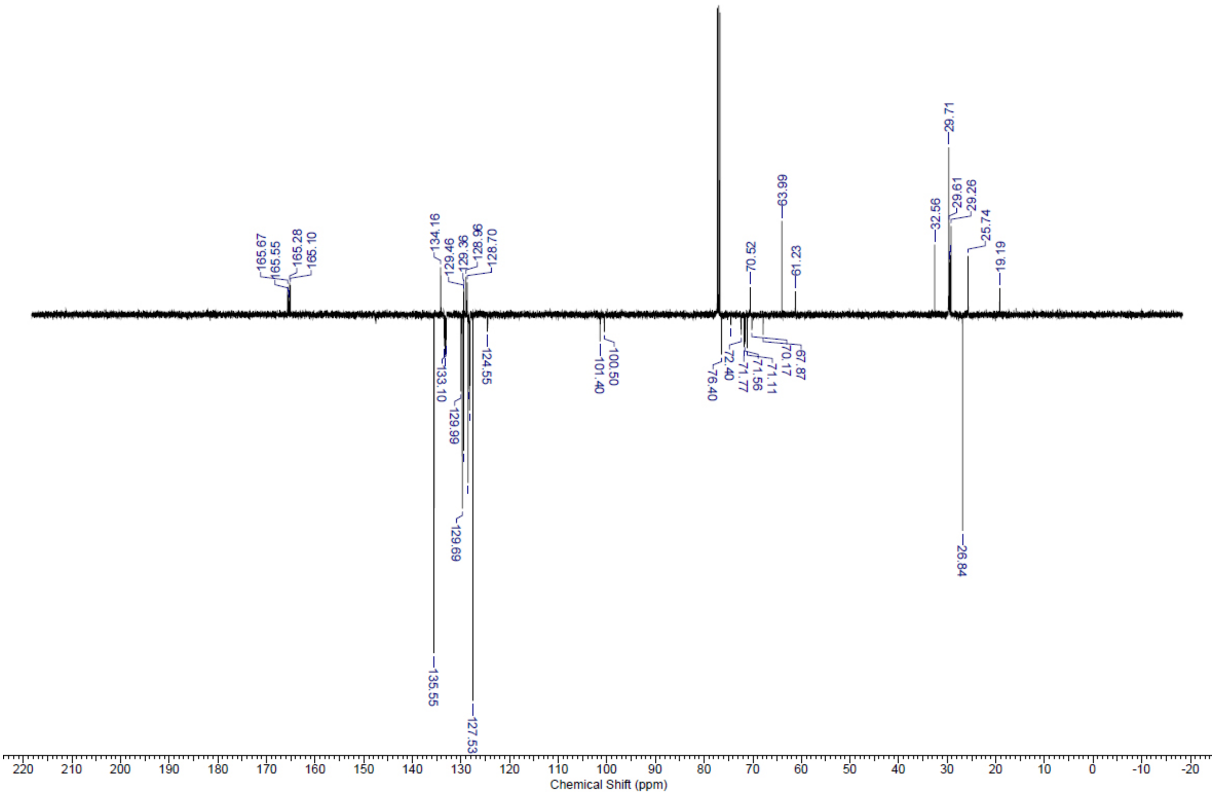
Chemical Shift (ppm)

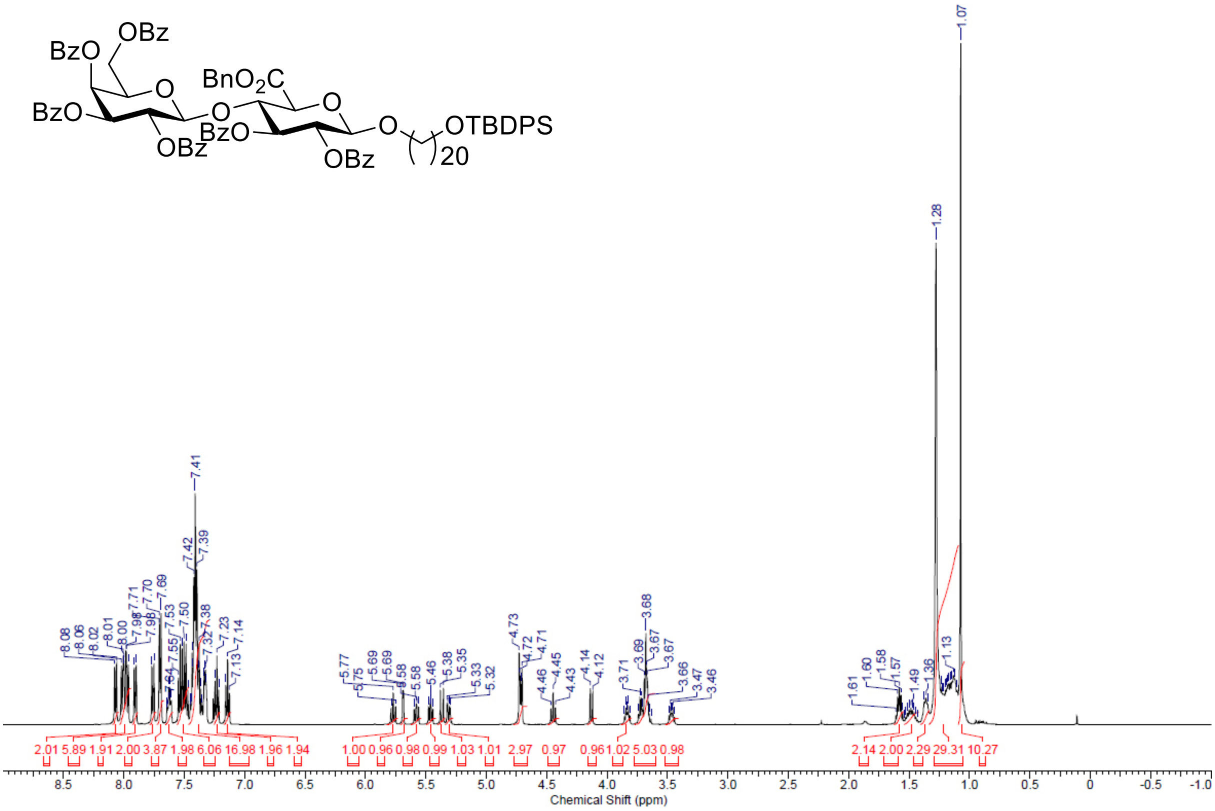
BzO
OBz
BzO
OBz
BnO2C
BzO
OBz
OTBDPS
20
Chemical Shift (ppm)

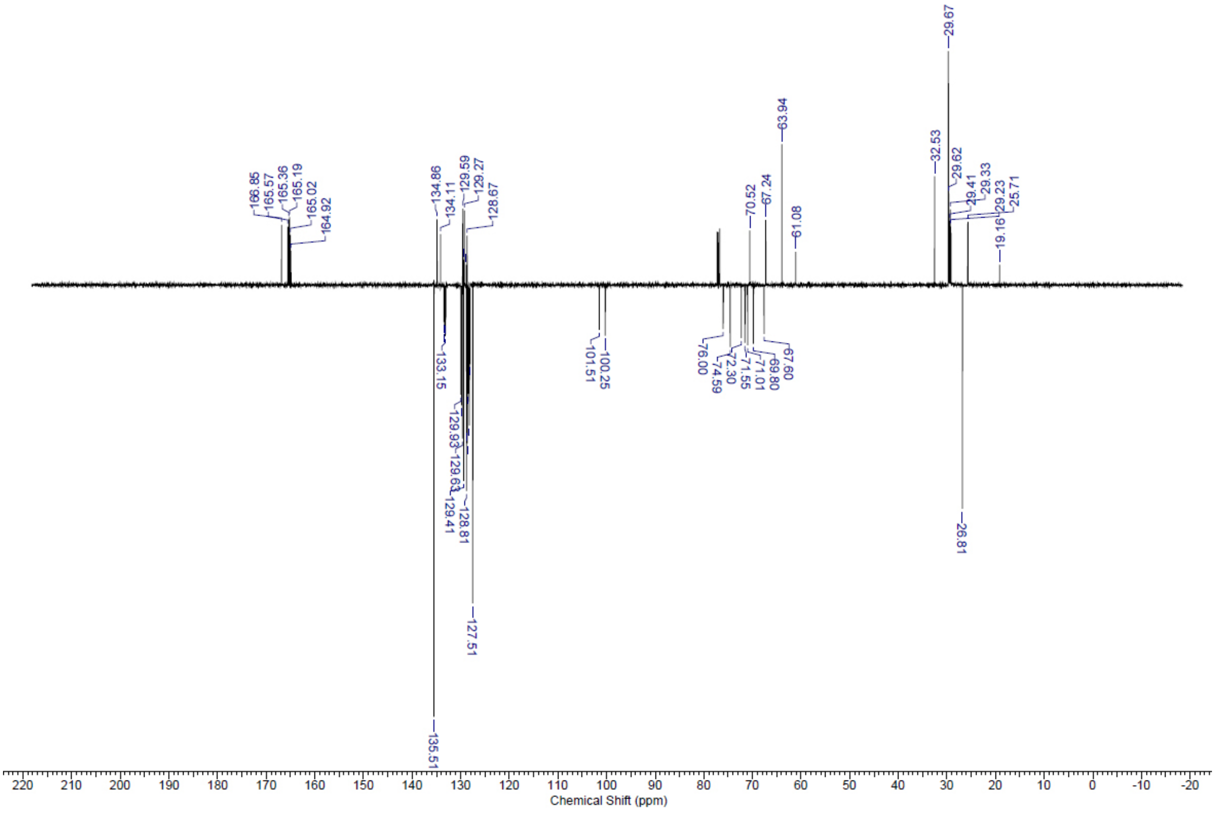
Chemical Shift (ppm)

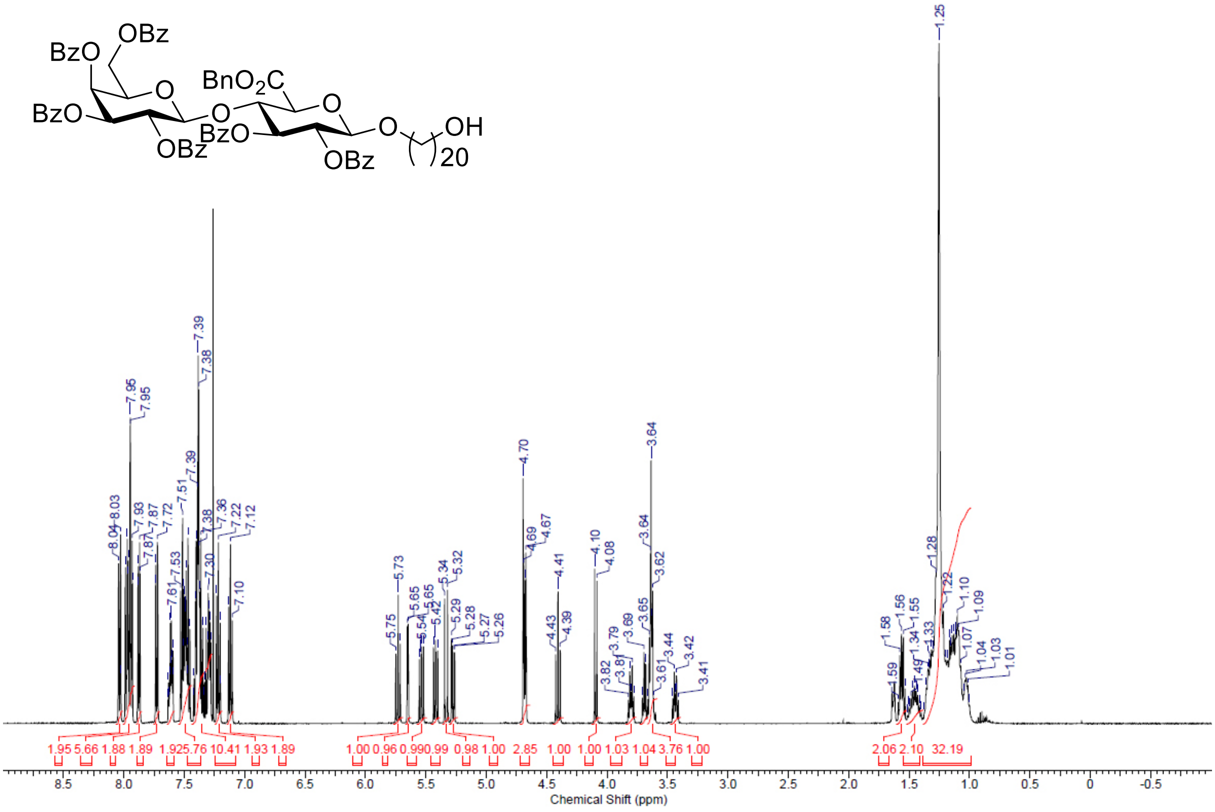
BzO
OBz
BzO
O
OBz
BnO2C
O
BzO
O
OBz
O
OH
20
Chemical Shift (ppm)

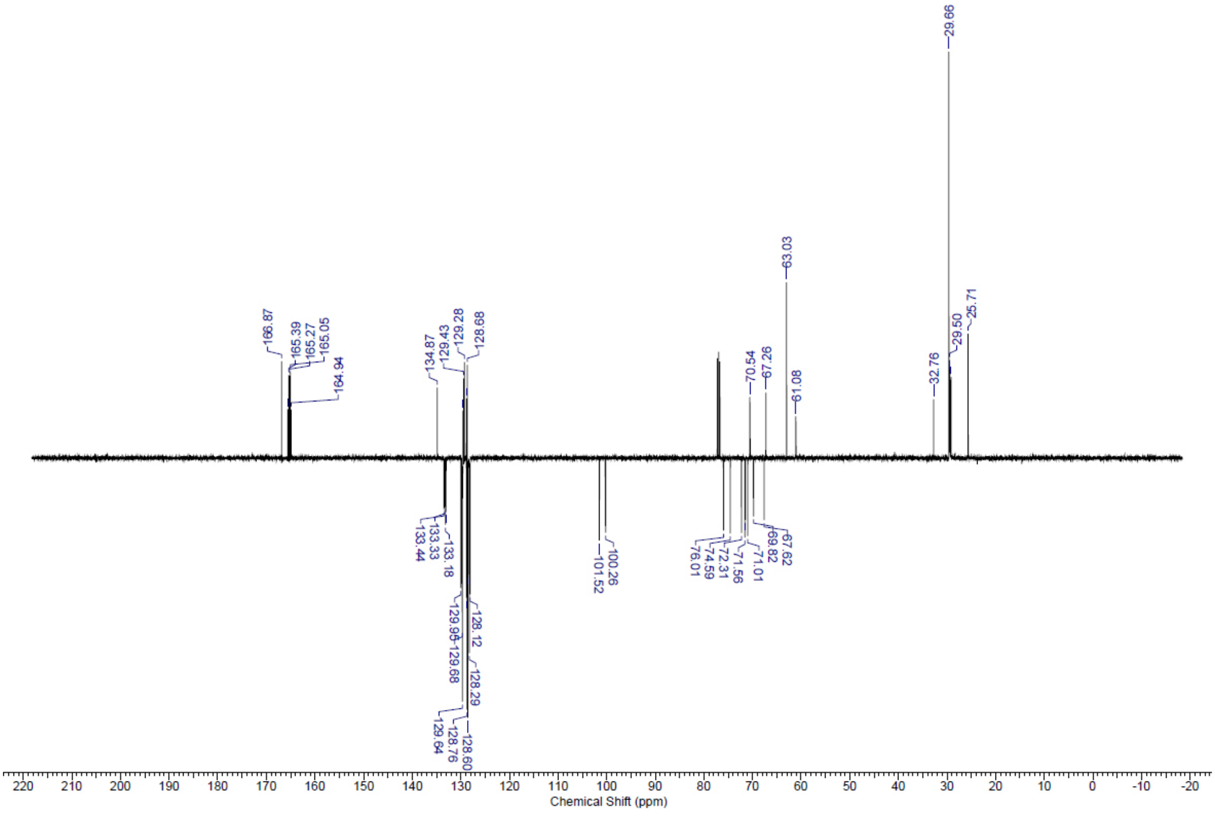
Chemical Shift (ppm)

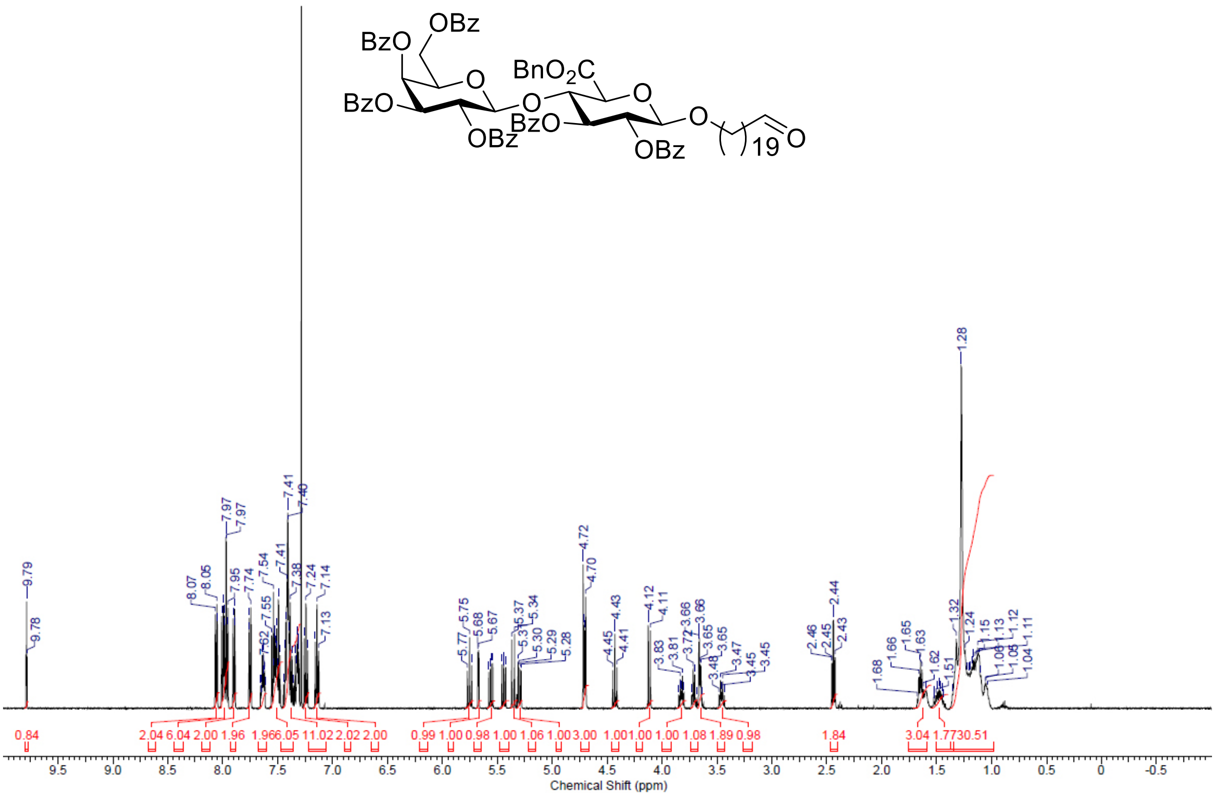

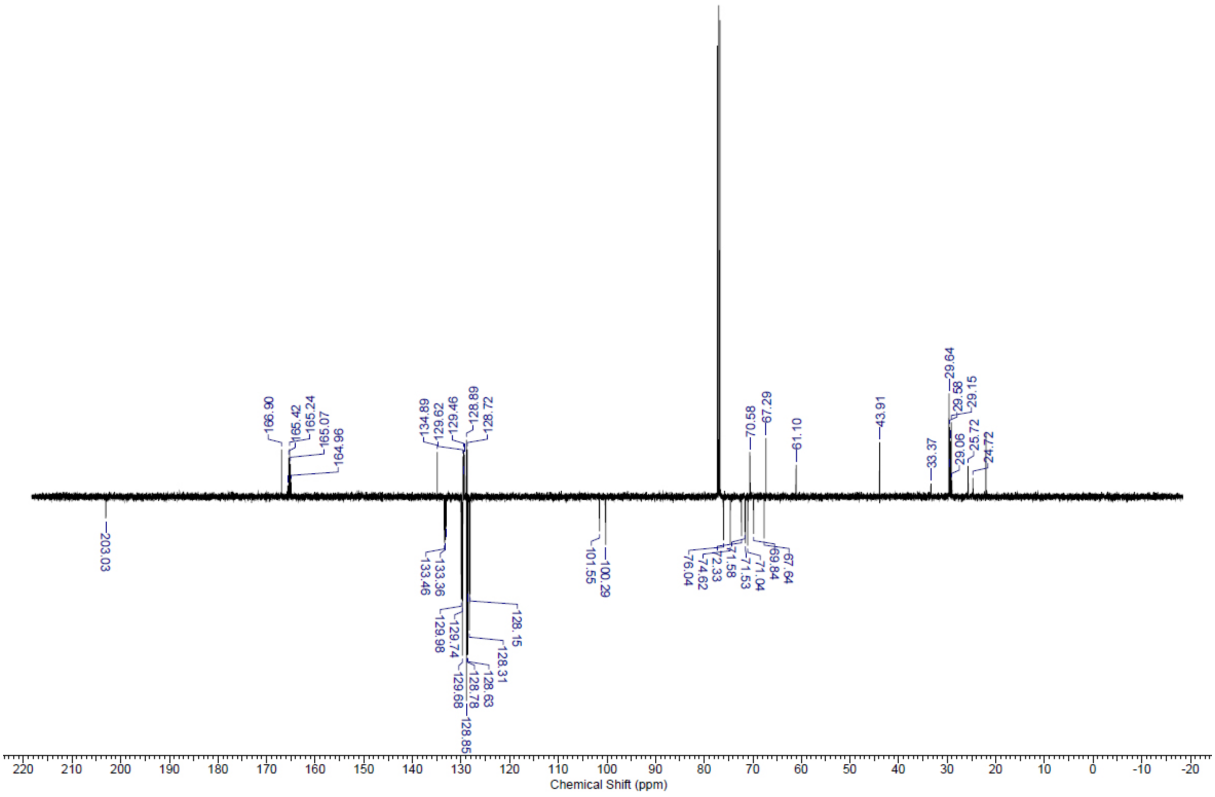

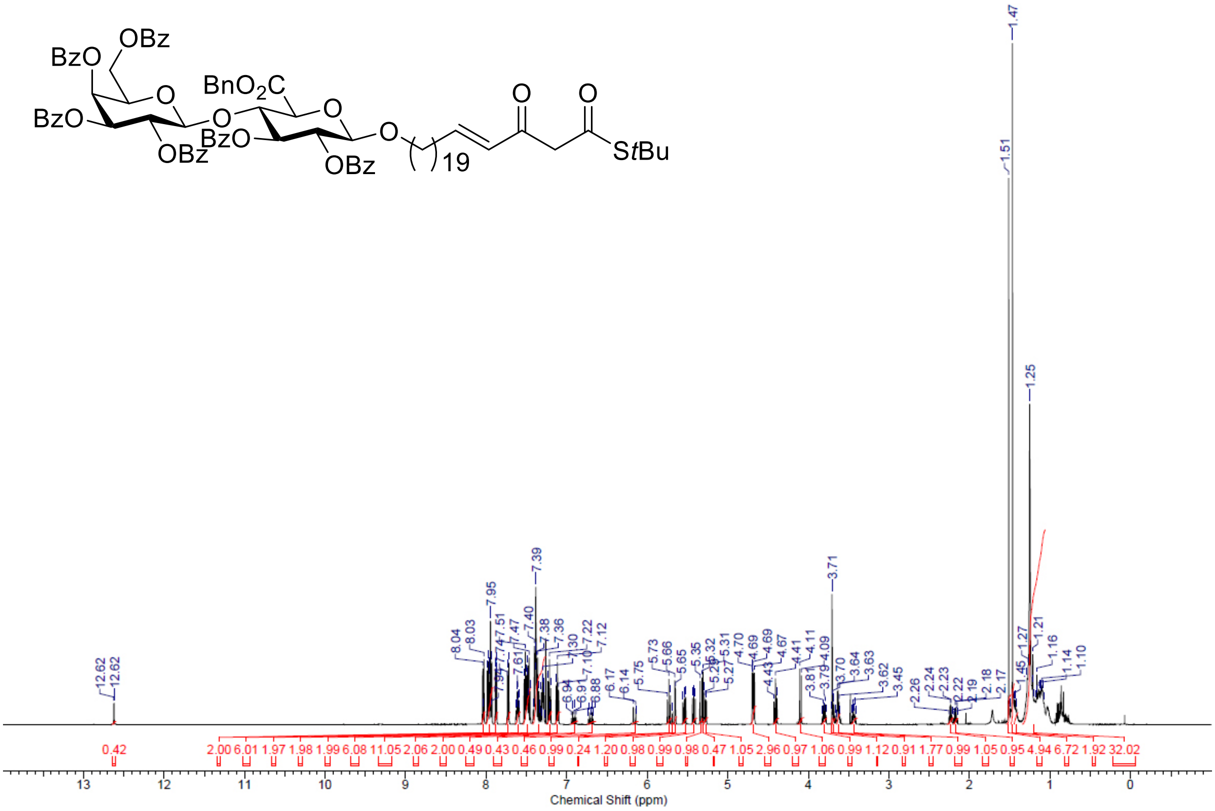

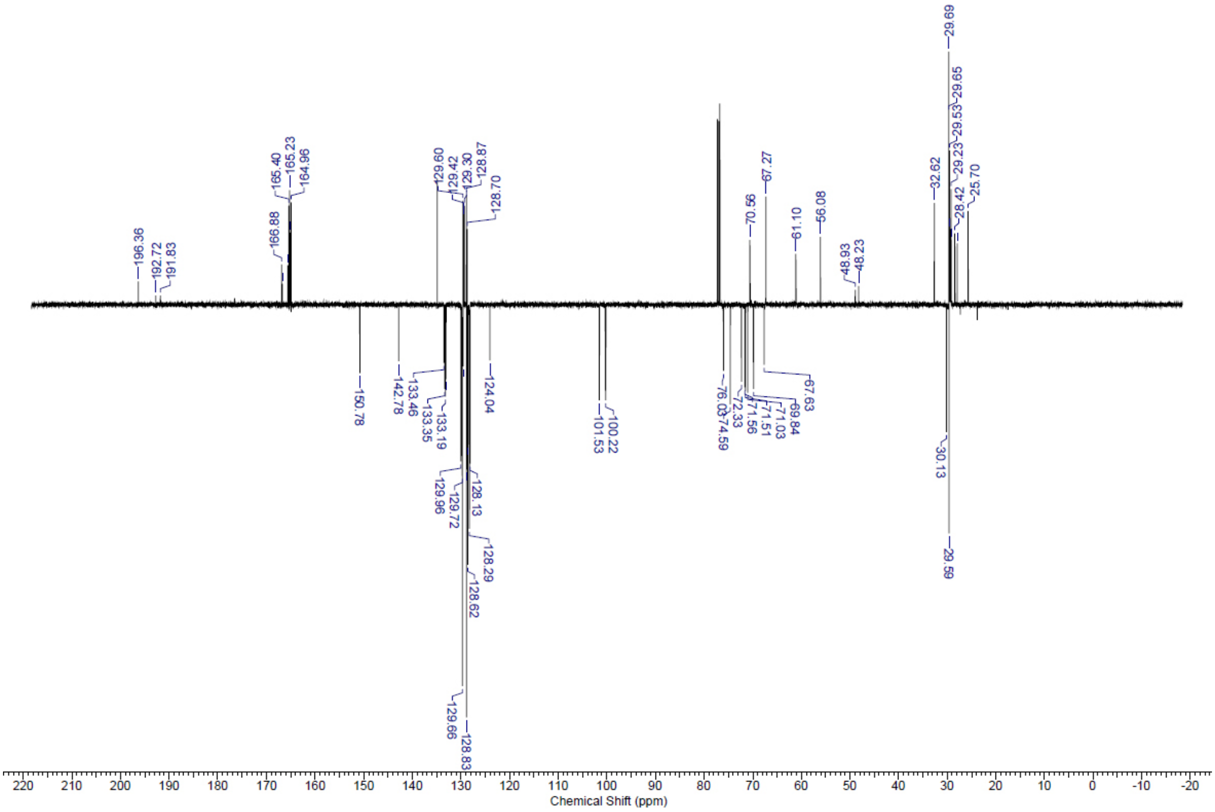

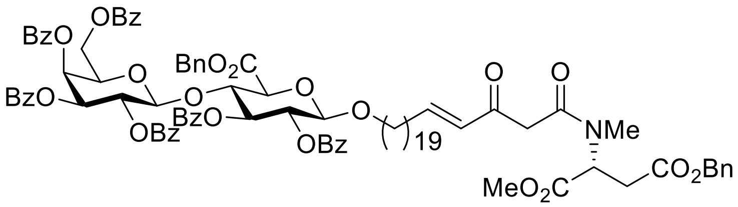

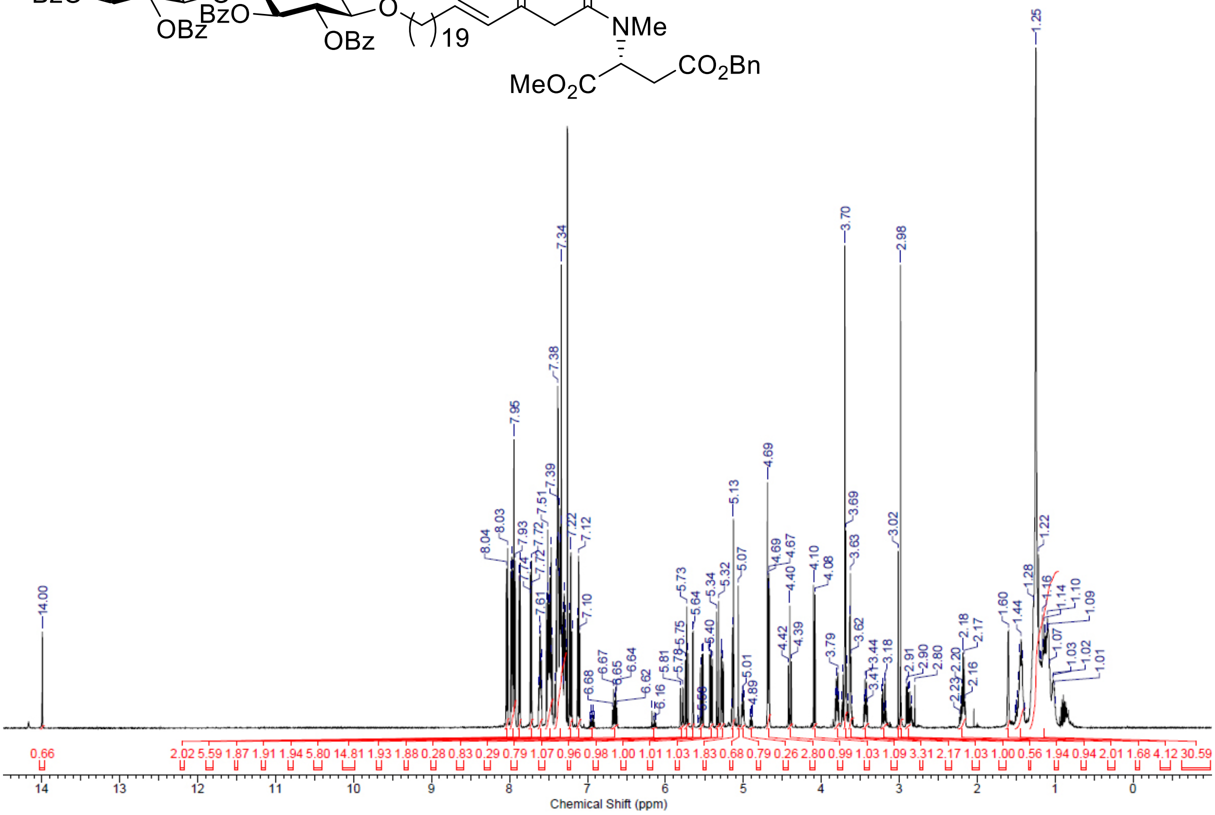
Chemical Shift (ppm)

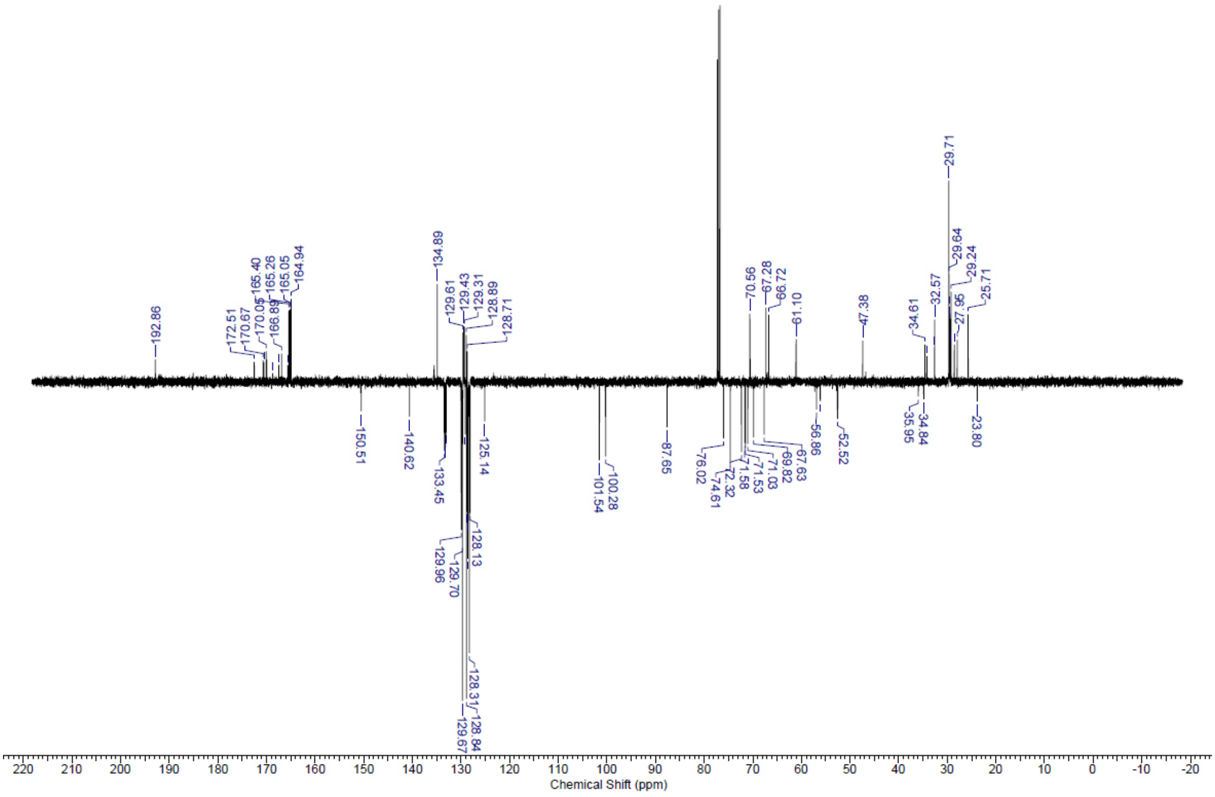
Chemical Shift (ppm)

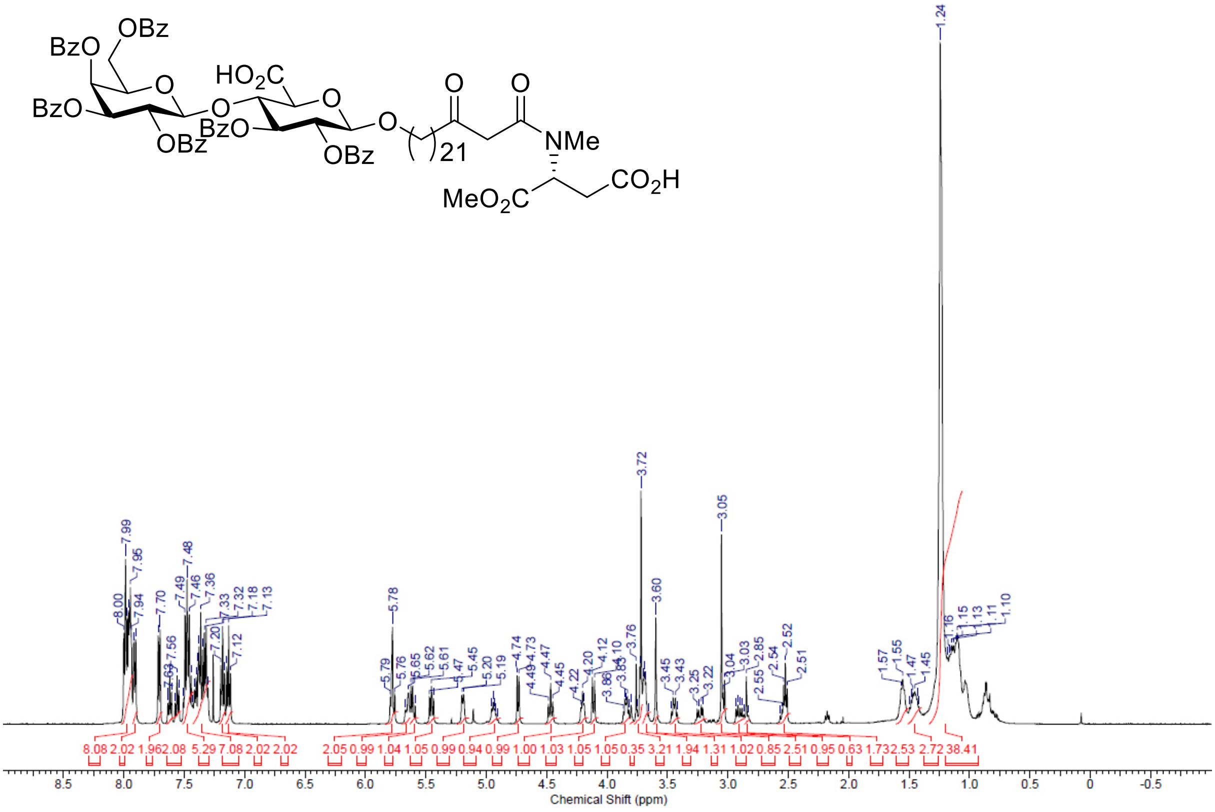
OBz
BzO
HO2C
BzO
OBz
BzO
OBz
O
O
21
NMe
CO2H
MeO2C
Chemical Shift (ppm)

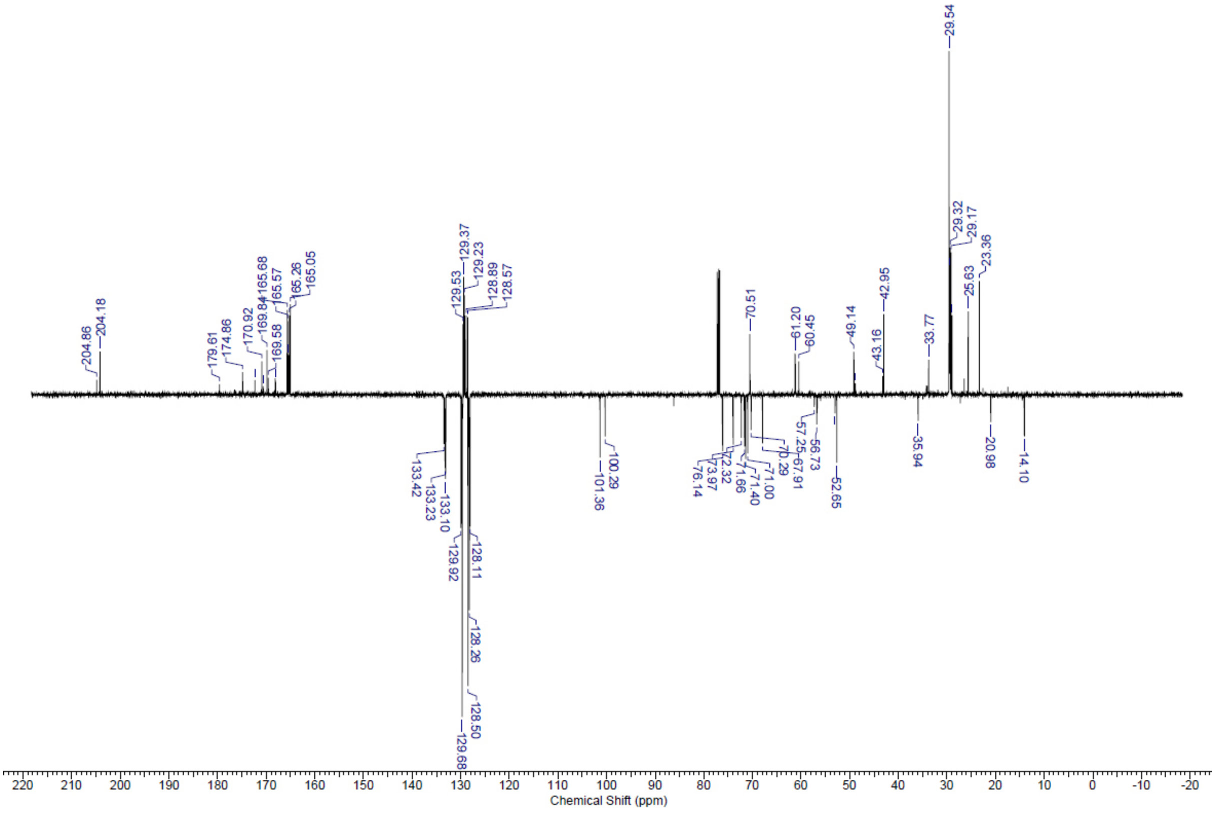
Chemical Shift (ppm)

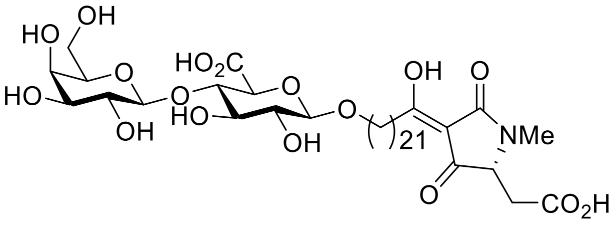
HO
OH
HO
O
O
HO2C
O
HO
OH
OH
O
OH
O
21
NMe
O
CO2H

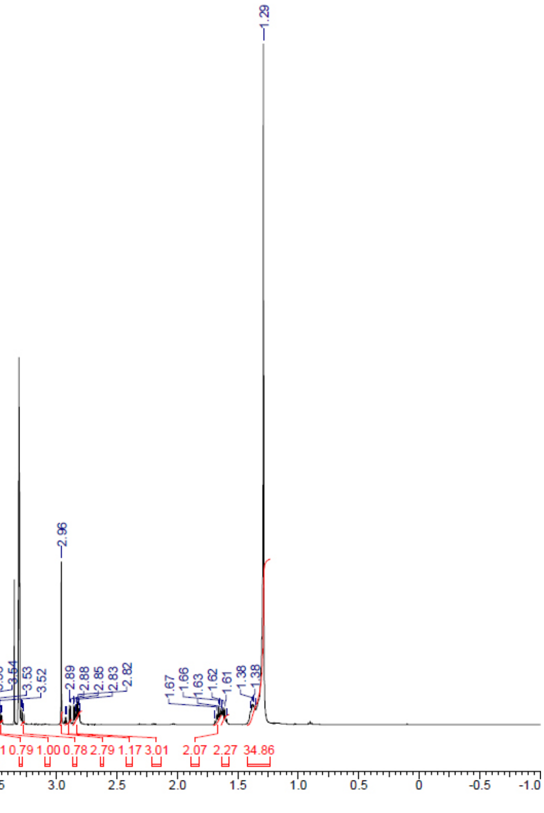
Chemical Shift (ppm)

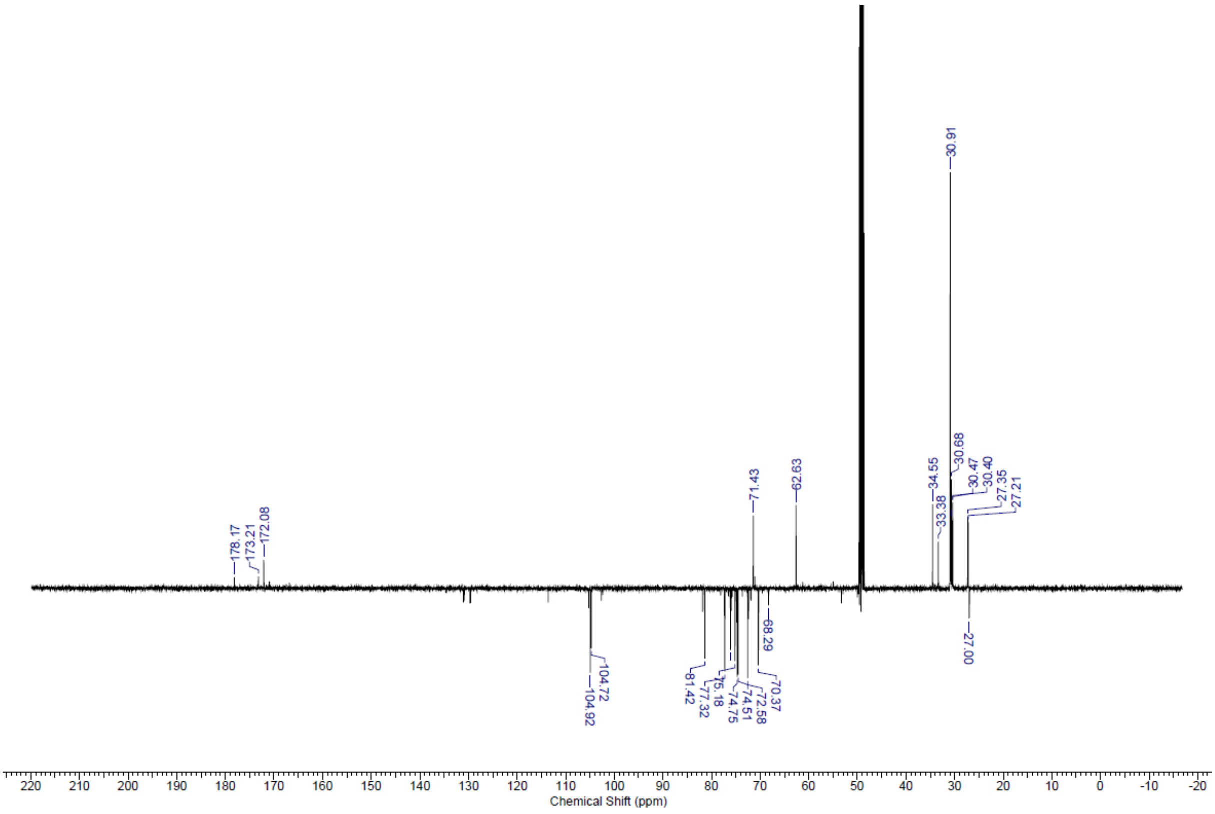
Chemical Shift (ppm)

COSY of **2**

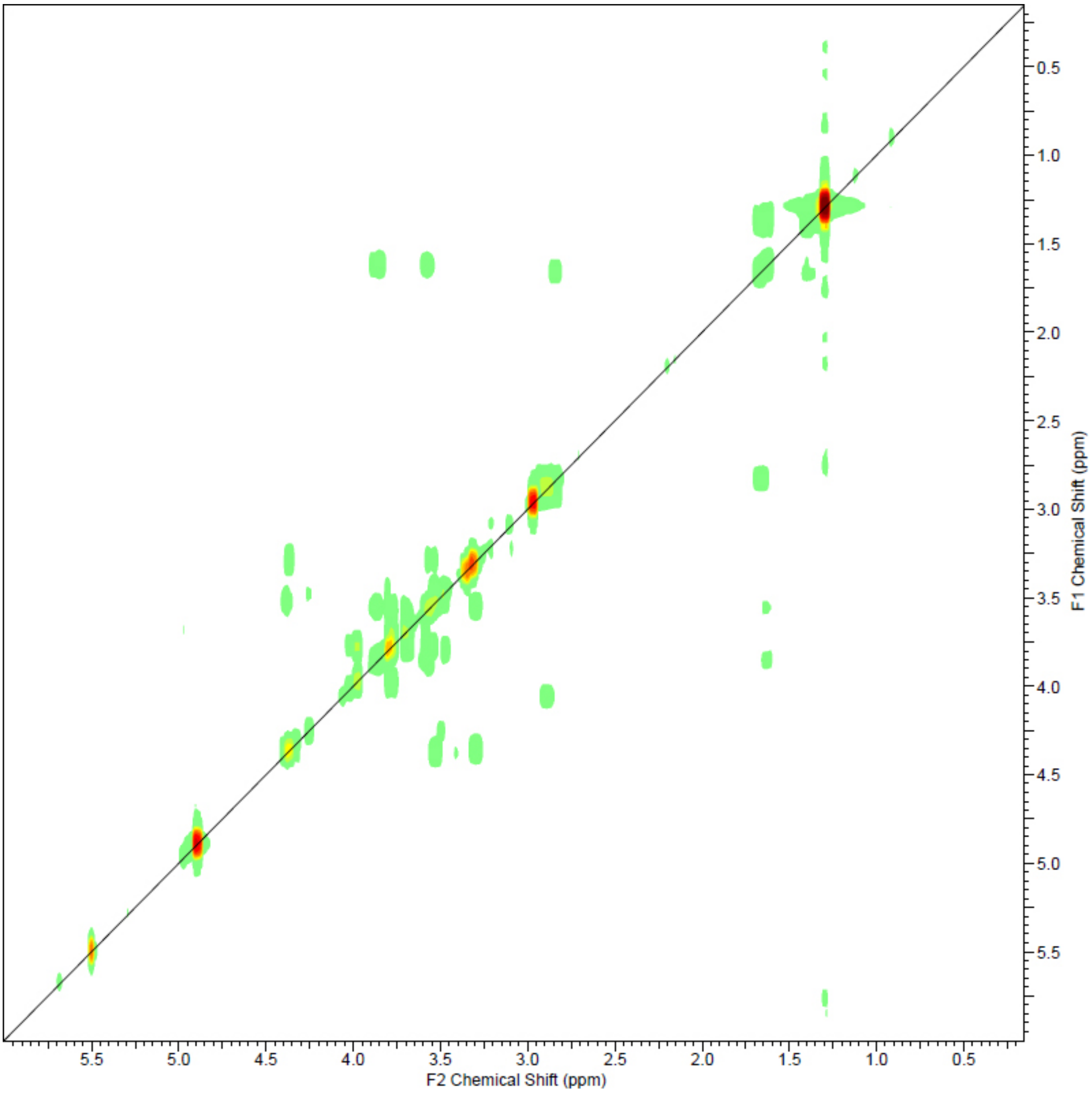

F2 Chemical Shift (ppm)
F1 Chemical Shift (ppm)
5.5
5.0
4.5
4.0
3.5
3.0
2.5
2.0
1.5
1.0
0.5

HSQC of **2**

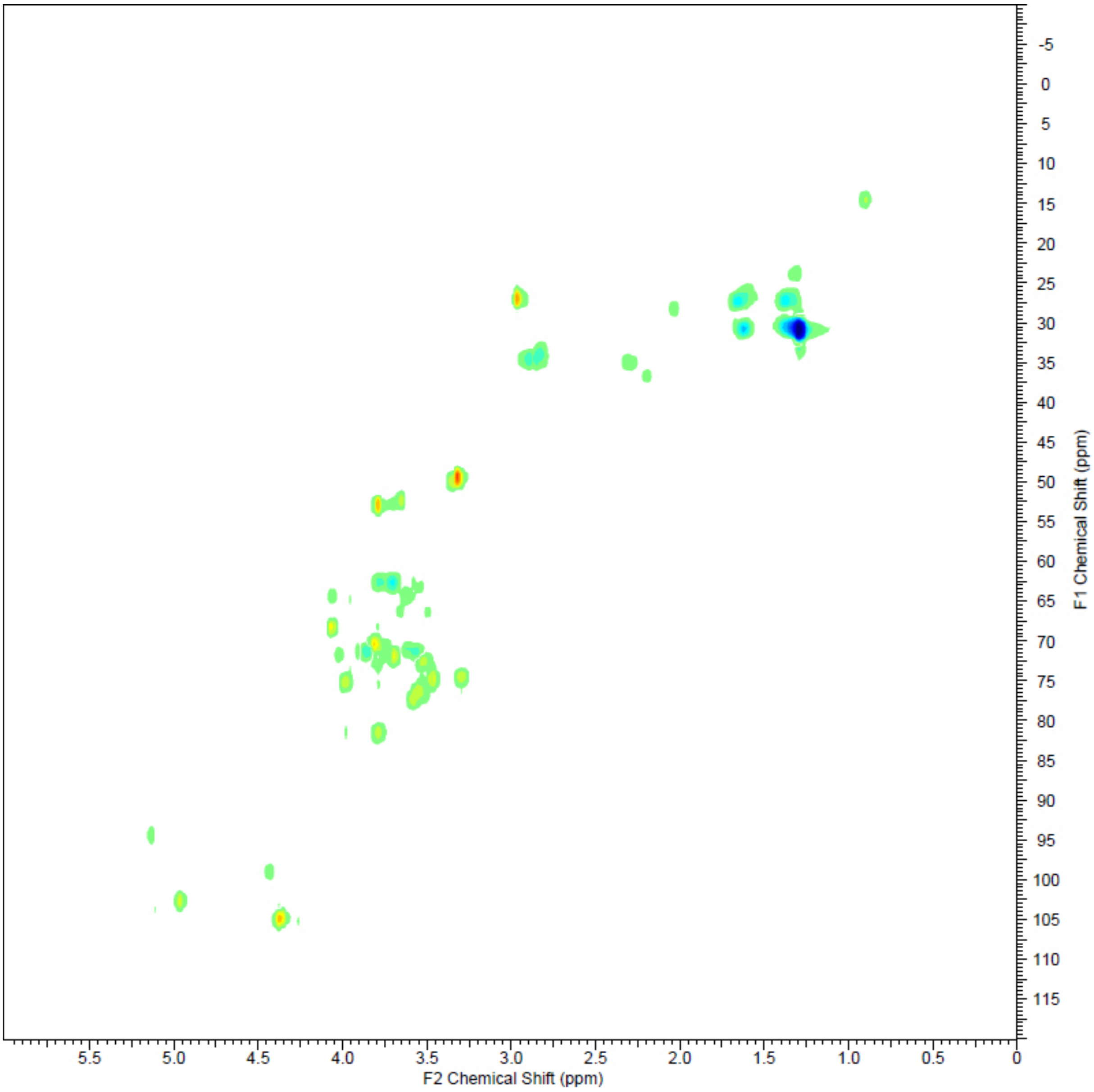

F2 Chemical Shift (ppm)
F1 Chemical Shift (ppm)
5.5
5.0
4.5
4.0
3.5
3.0
2.5
2.0
1.5
1.0
0.5
0
-5
0
5
10
15
20
25
30
35
40
45
50
55
60
65
70
75
80
85
90
95
100
105
110
115

HMBC of **2**

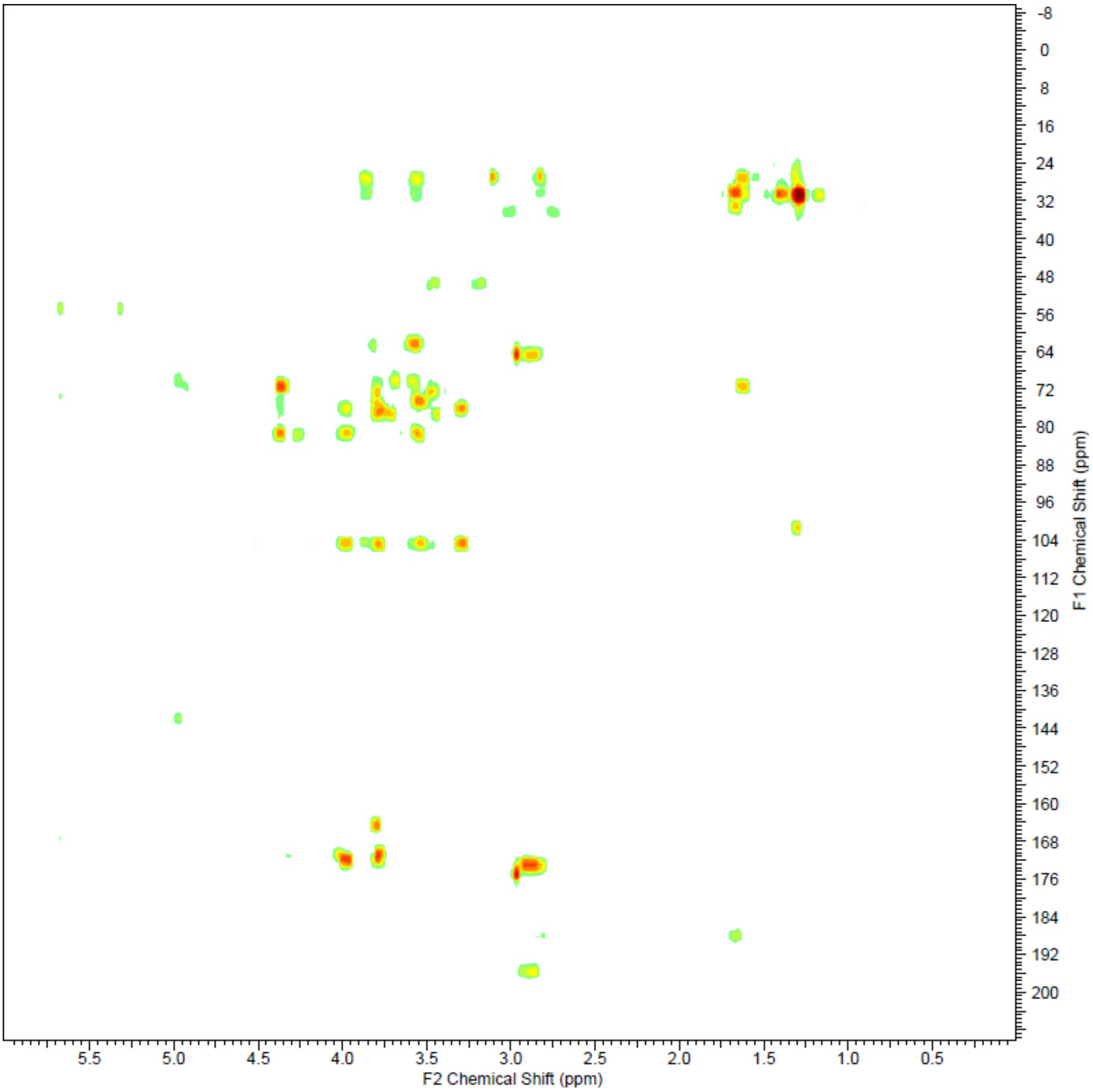

HPLC chromatogram of **2**

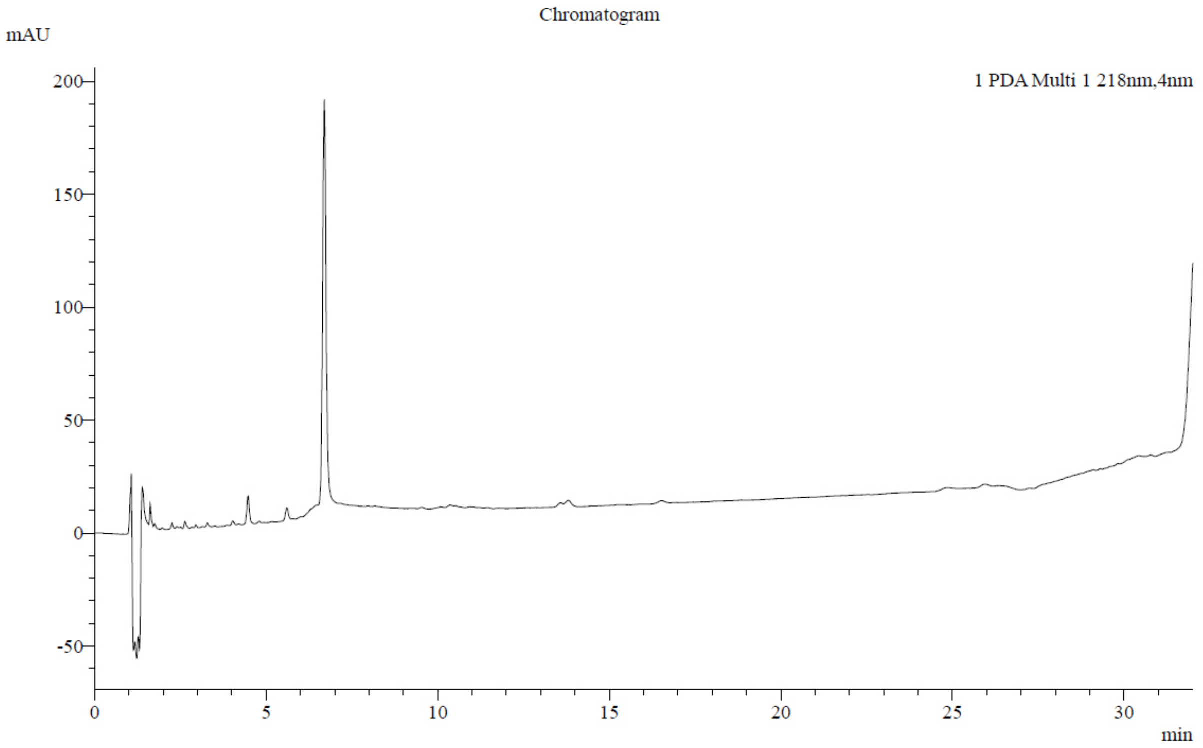

HPLC program: start at 70% MeCN in H2O with 0.1% HCOOH to 85% MeCN in 25 min for 5 min;
Flow rate 1.0 mL/min, column: Knauer Eurospher II 100-3 C18, 150 x 4 mm

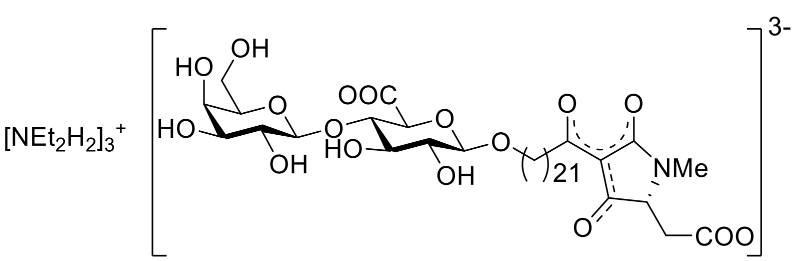
[NEt2H2]3+
3-
HO
OH
OOC
O
HO
OH
NMe
21
COO

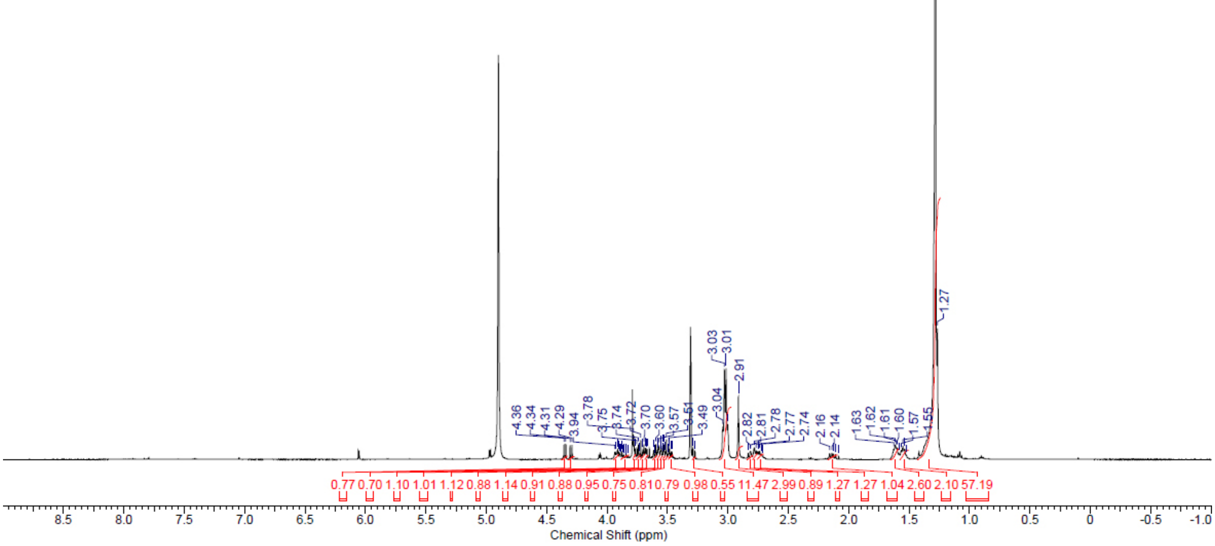
Chemical Shift (ppm)

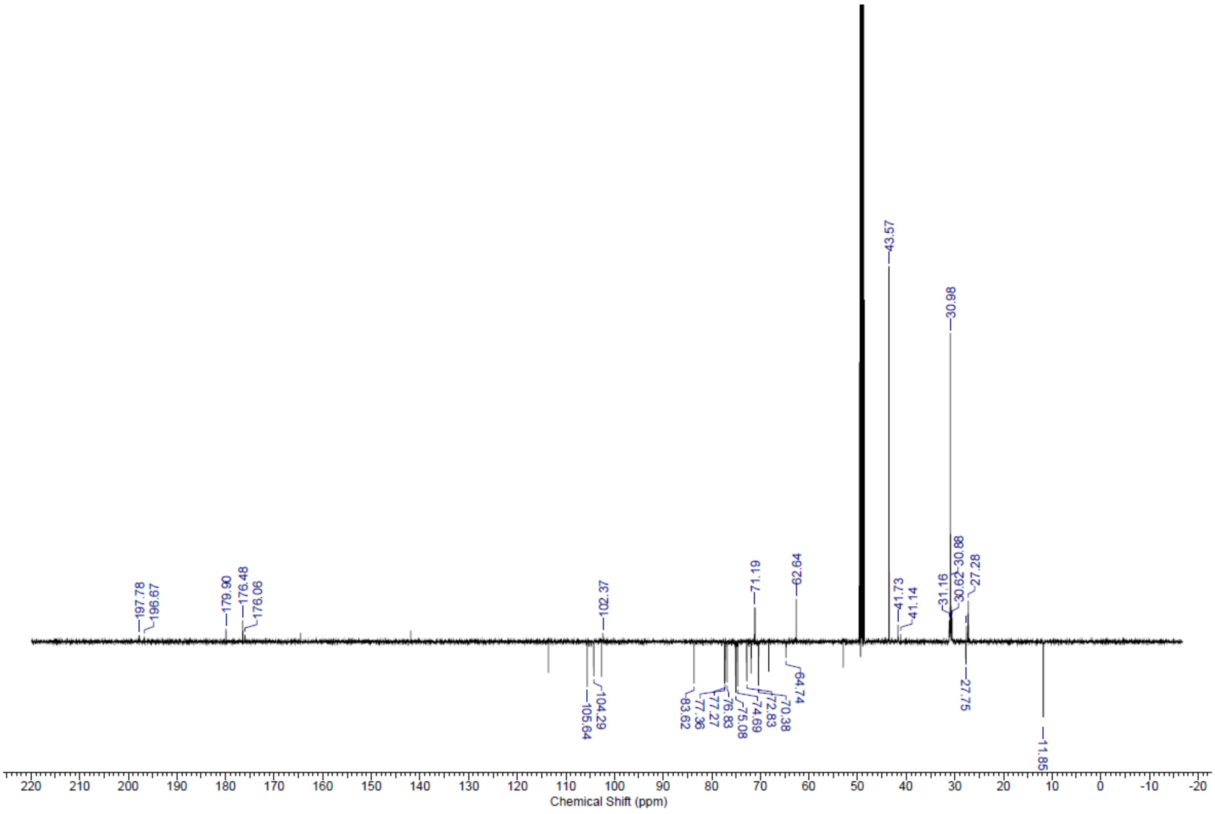
Chemical Shift (ppm)

COSY of $[NEt_2H_2]_3^+$ [**2**-3H]$^{3-}$

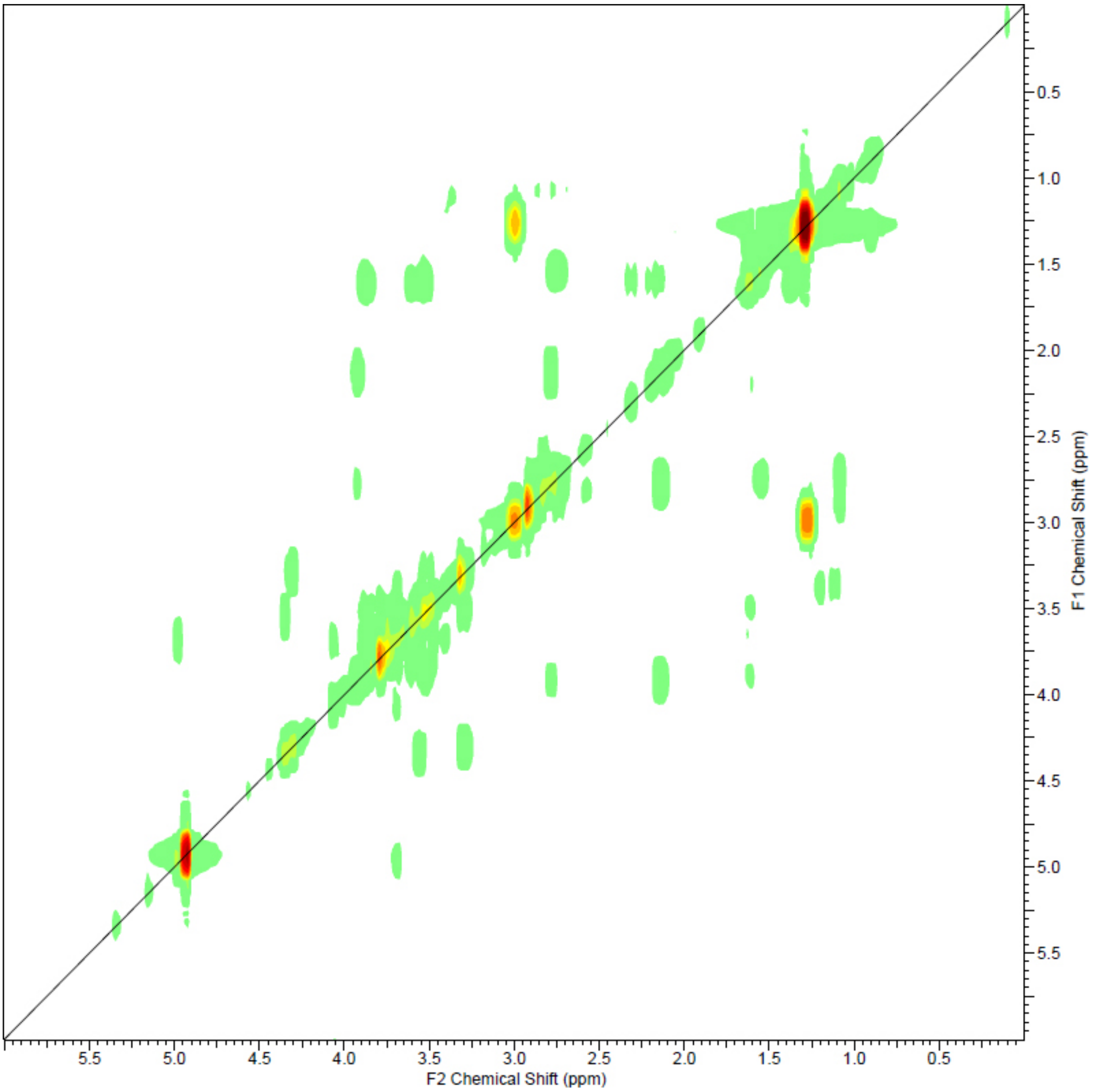

HSQC of $[NEt_2H_2]_3^+$ [**2**-3H]$^{3-}$

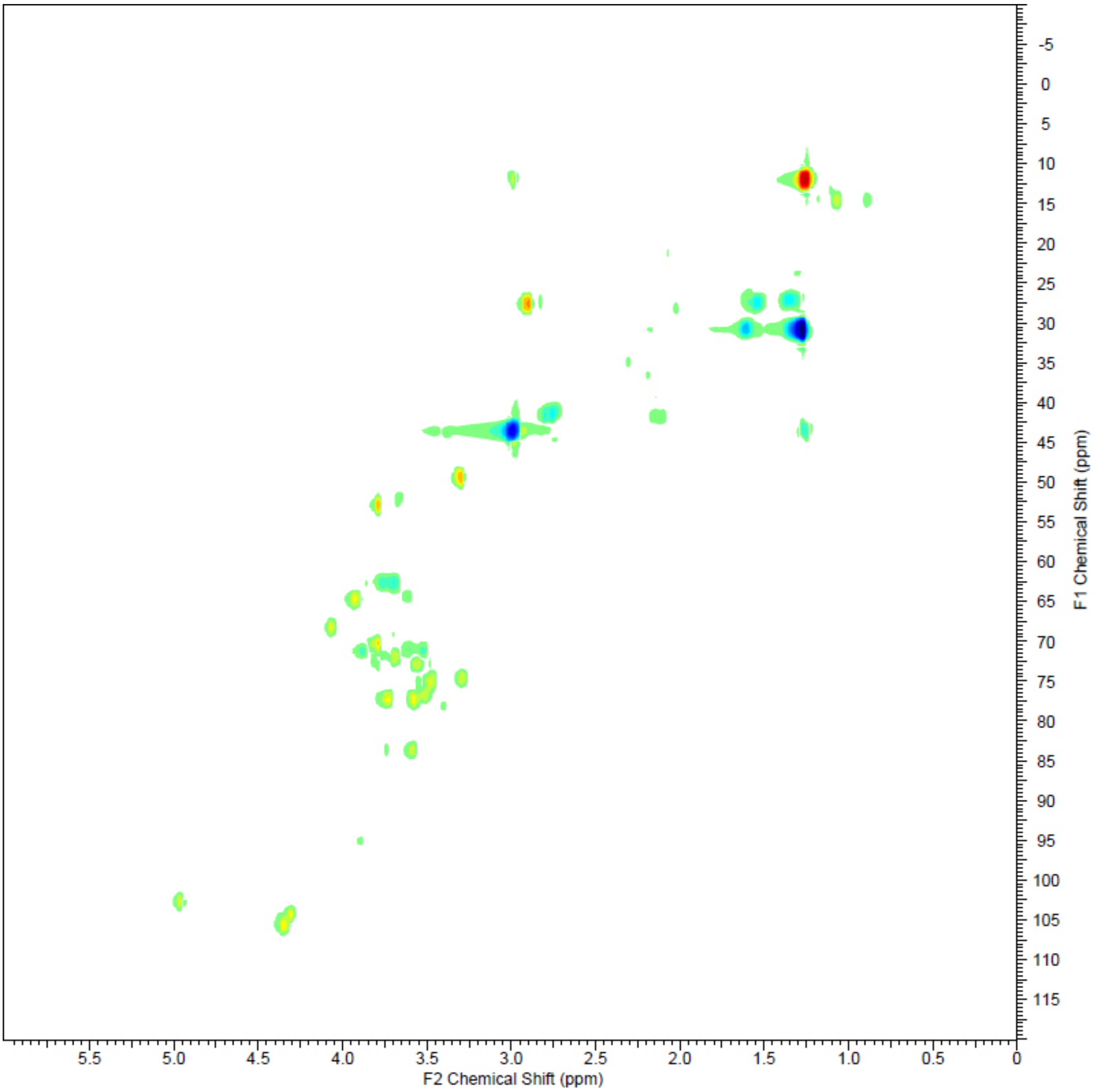

HMBC of $[NEt_2H_2]_3^+$ [**2**-3H]$^{3-}$

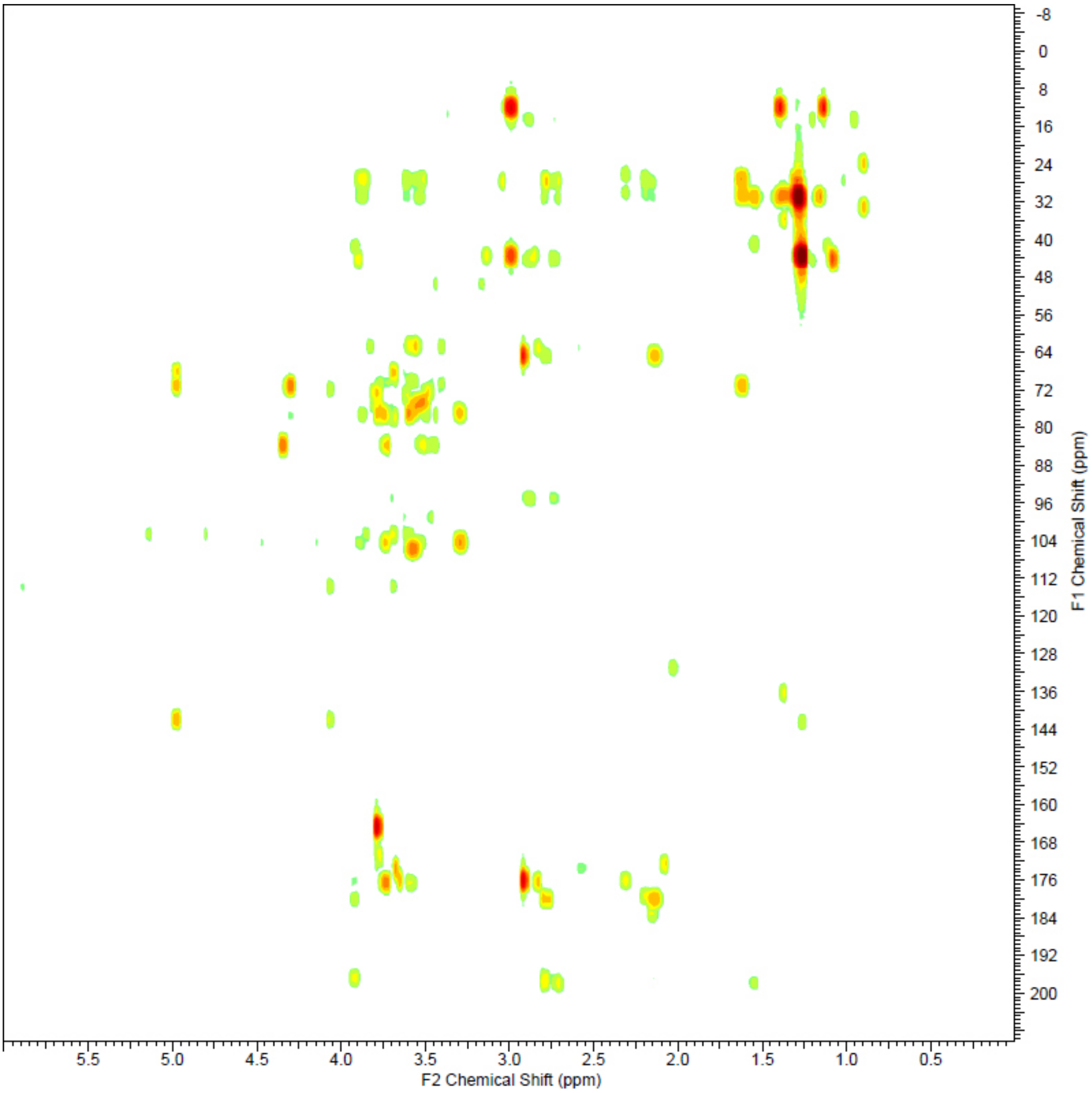

HPLC chromatogram of $[NEt_2H_2]_3^+$ [**2**-3H]$^{3-}$

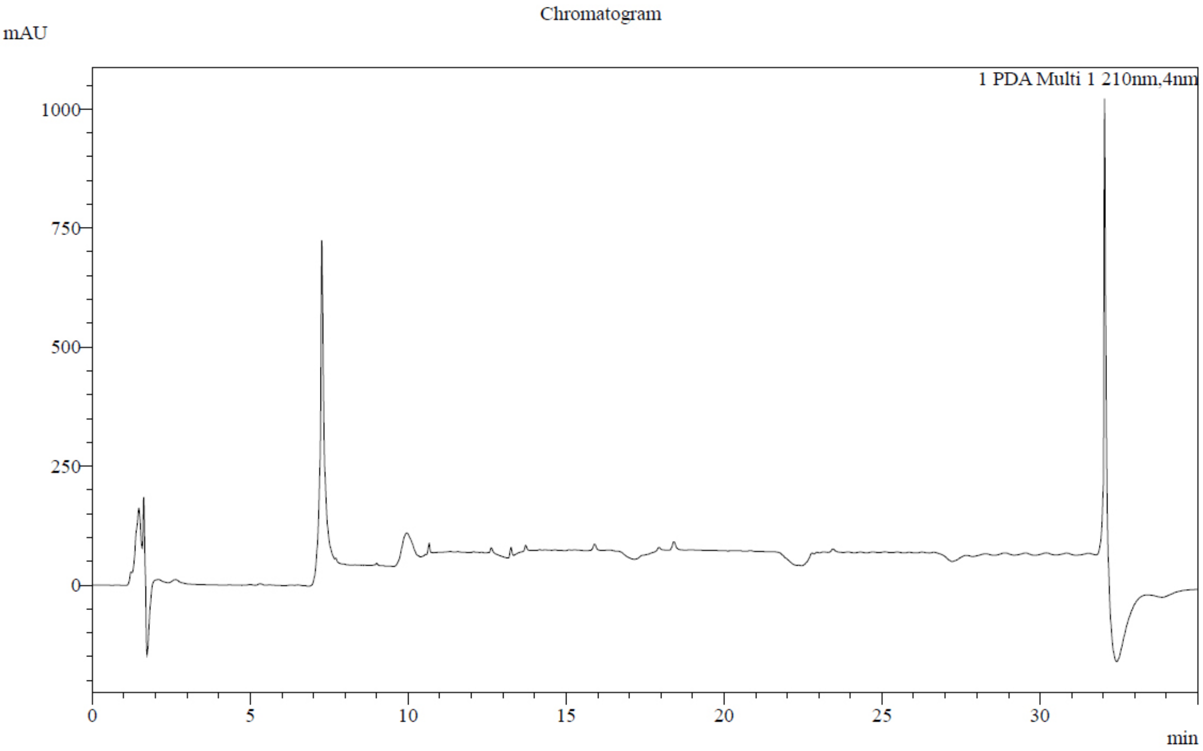

HPLC program: start at 40% MeCN in H2O to 95% MeCN in 25 min for 5 min;
Flow rate 1.0 mL/min, column: Knauer Eurospher II 100-3 C18, 150 x 4 mm

HRMS of ancorinoside B (2)

Analysis without HPLC-column, full scan mode 80-1200 amu, iso80: isocratic with 80% MeCN

ESI positive:

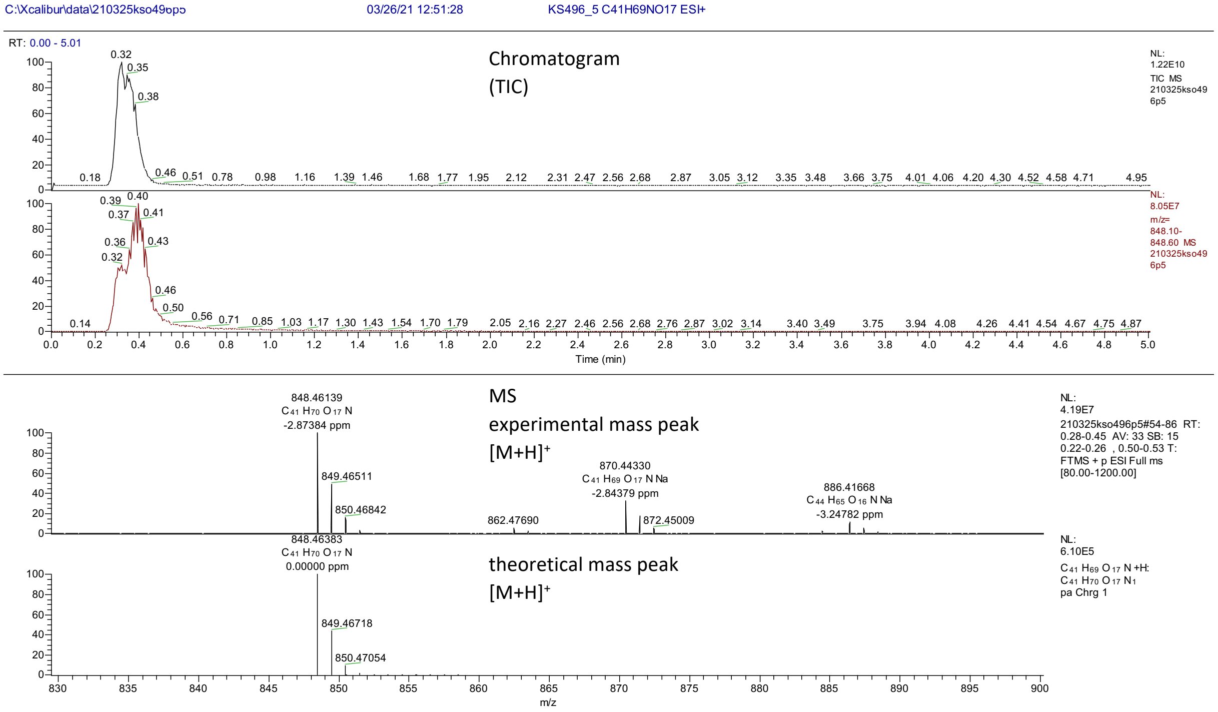

ESI negative:

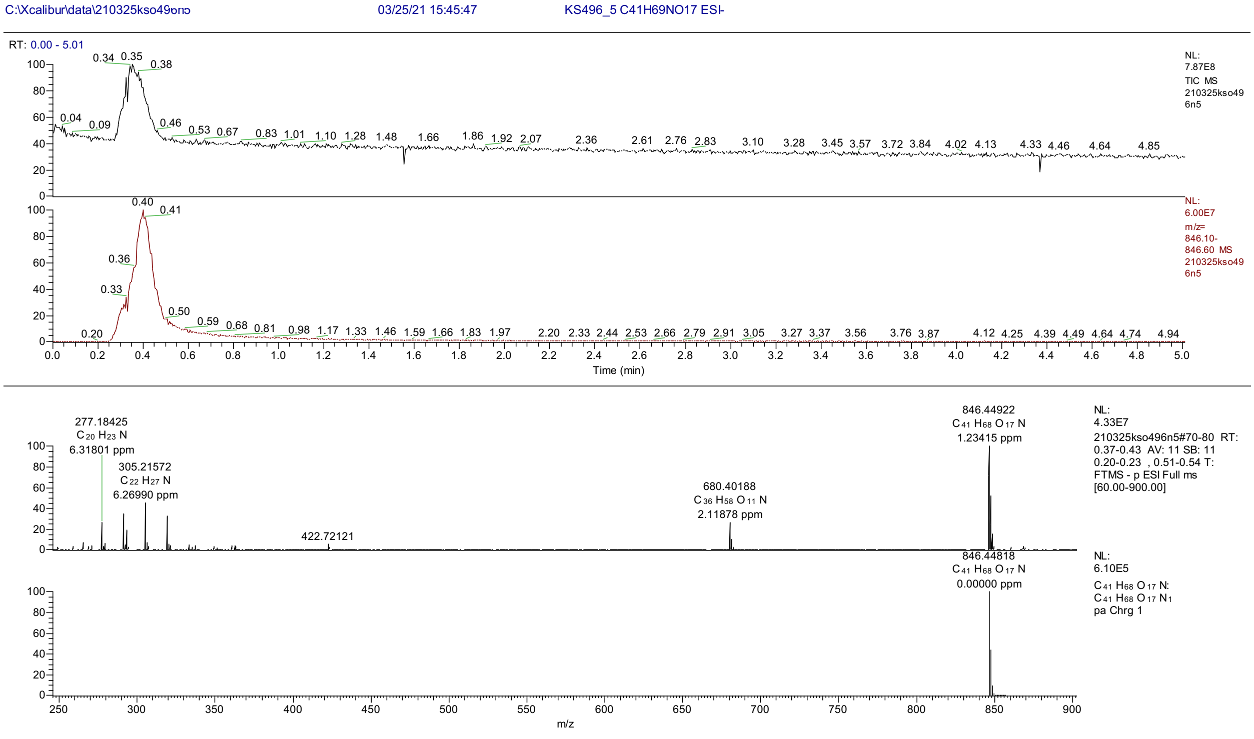

Analysis with HPLC-column (Accupore RP-MS 150 x 2.1 mm, 2.6 µm), full scan mode 100-1500 amu, gradient from 40% to 90% MeCN

ESI positive

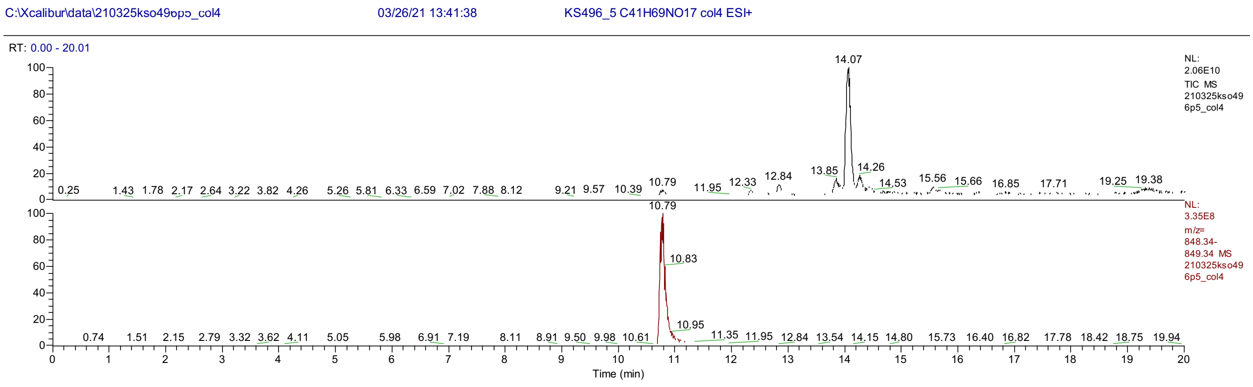

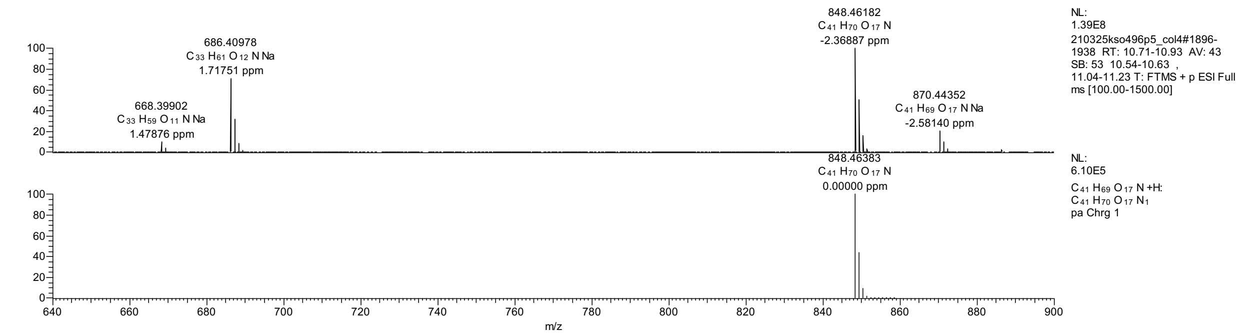

ESI negative

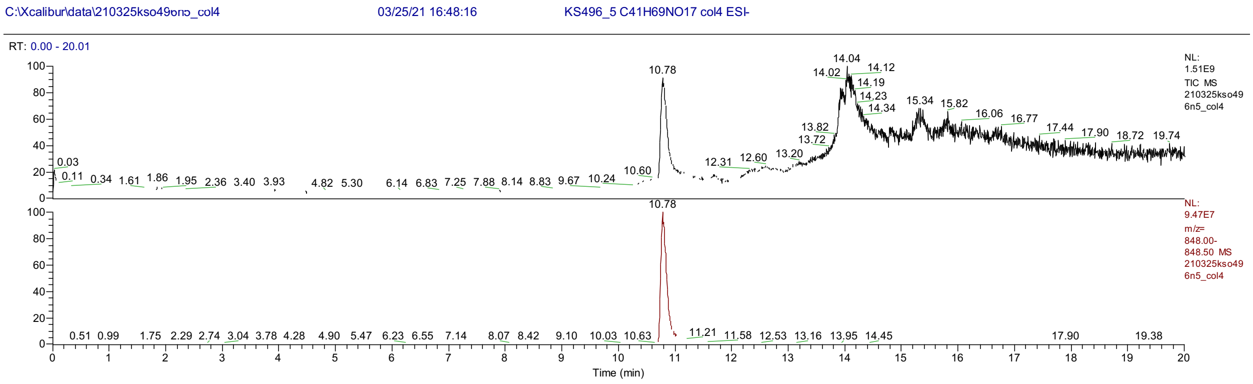

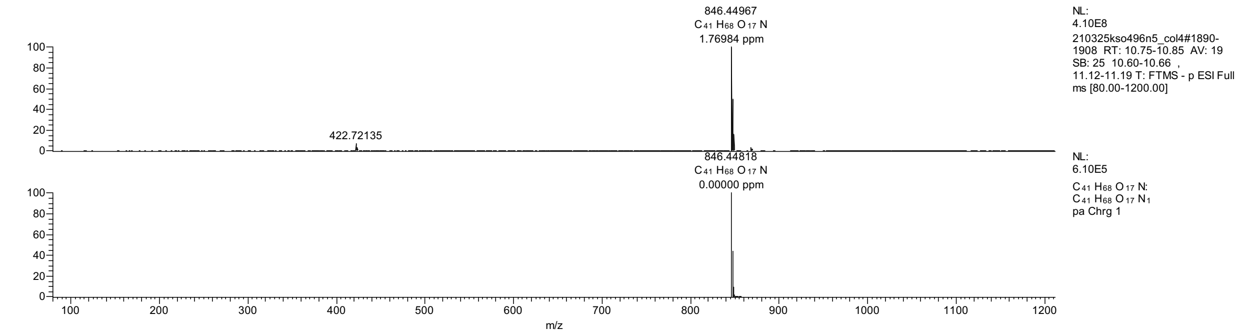

HRMS of 8

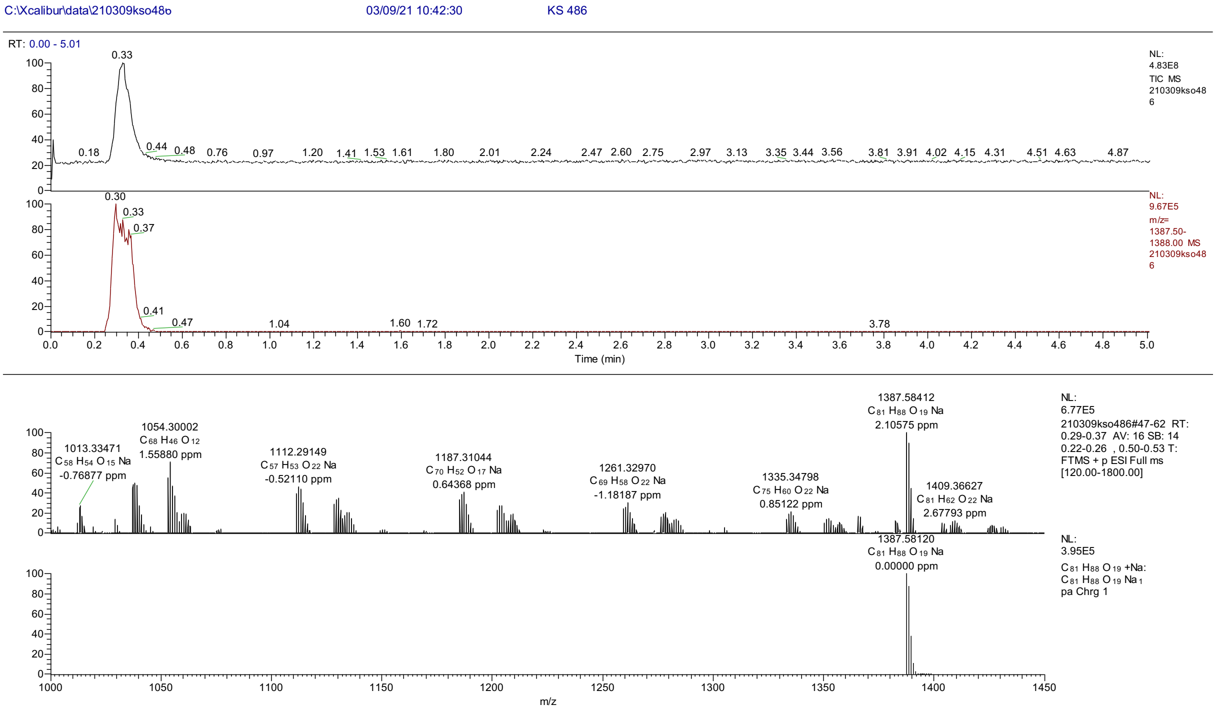

HRMS of 6

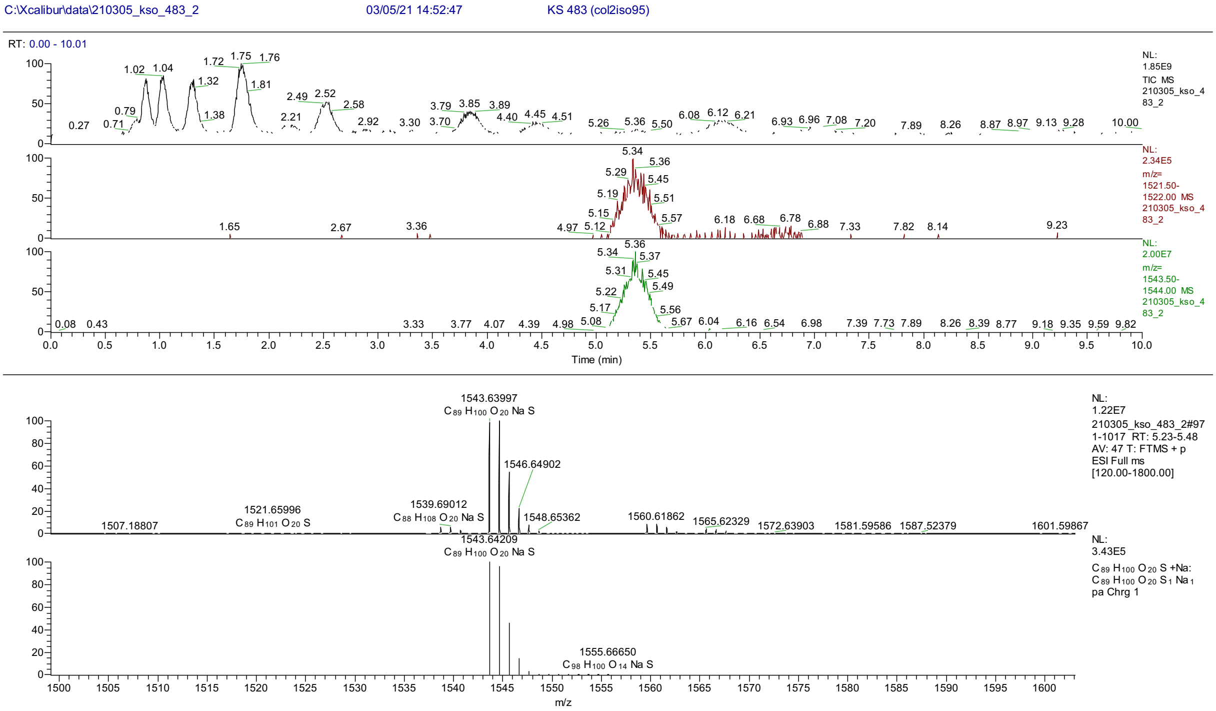

HRMS of 5

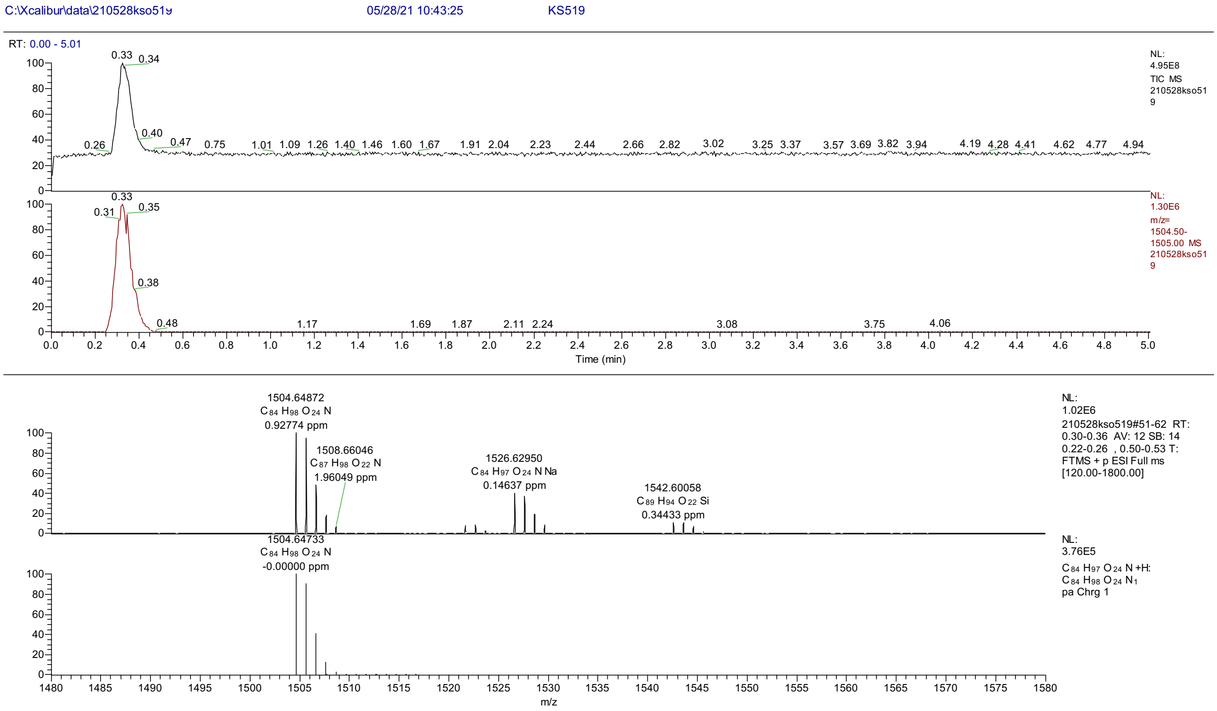

C:\Xcalibur\data\210528kso519
05/28/21 10:43:25
KS519
RT: 0.00 - 5.01
NL: 4.95E8 TIC MS 210528kso519
NL: 1.30E6 m/z= 1504.50-1505.00 MS 210528kso519
Time (min)
1504.64872
C84 H98 O24 N
0.92774 ppm
1508.66046
C87 H98 O22 N
1.96049 ppm
1526.62950
C84 H97 O24 N Na
0.14637 ppm
1542.60058
C89 H94 O22 Si
0.34433 ppm
NL: 1.02E6
210528kso519#51-62 RT: 0.30-0.36 AV: 12 SB: 14 0.22-0.26 , 0.50-0.53 T: FTMS + p ESI Full ms [120.00-1800.00]
1504.64733
C84 H98 O24 N
-0.00000 ppm
NL: 3.76E5
C84 H97 O24 N +H: C84 H98 O24 N1
pa Chrg 1
m/z

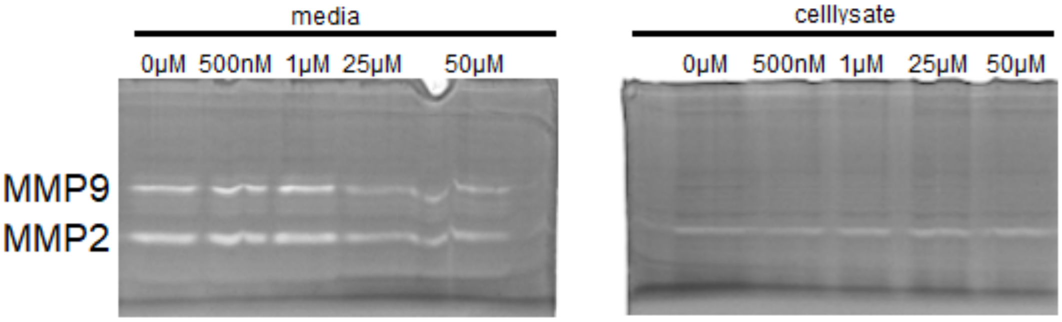

Figure S1. Effect of ancorinoside B (**2**) (500 nM, 1 µM, 25 µM, and 50 µM) on the cell-associated and secreted activities of MMP-2 and MMP-9 in 518A2 melanoma cells after 24 h exposure. Cell lysate and media samples were subjected to gelatin zymography. Control (DMSO)-treated probes are listed as 0 µM. Images are representative of two independent experiments.
